# Practical Synthesis of Phosphinic Dipeptides by Tandem Esterification of Aminophosphinic and Acrylic Acids under Silylating Conditions

**DOI:** 10.3390/molecules27041242

**Published:** 2022-02-12

**Authors:** Paraskevi Kokkala, Kostas Voreakos, Angelos Lelis, Konstantinos Patiniotis, Nikolaos Skoulikas, Laurent Devel, Angeliki Ziotopoulou, Eleni Kaloumenou, Dimitris Georgiadis

**Affiliations:** 1 Laboratory of Organic Chemistry, Department of Chemistry, National and Kapodistrian University of Athens, Panepistimiopolis, Zografou, 15784 Athens, Greece; pkokkala@chem.uoa.gr (P.K.); chemnite@chem.uoa.gr (K.V.); angeloslelis@gmail.com (A.L.); konstantinp@chem.uoa.gr (K.P.); nikolas.skoulikas@gmail.com (N.S.); kelisia1995@hotmail.com (A.Z.); lenakal17@gmail.com (E.K.); 2 Département Médicaments et Technologies pour la Santé (DMTS), CEA, INRAE, SIMoS, Université Paris-Saclay, 91191 Gif-sur-Yvette, France; laurent.devel@cea.fr

**Keywords:** phosphinic, pseudopeptides, *P*-Michael, silylation, hexamethyldisilazane, inhibitors, Zn-metalloproteases, acrylic acids, aminophosphinic, organophosphorus

## Abstract

In this report, a synthetic protocol for the preparation of phosphinic dipeptides of type **5** is presented. These compounds serve as valuable building blocks for the development of highly potent phosphinopeptidic inhibitors of medicinally relevant Zn-metalloproteases and aspartyl proteases. The proposed method is based on the tandem esterification of α-aminophosphinic and acrylic acids under silylating conditions in order to subsequently participate in a *P*-Michael reaction. The scope of the transformation was investigated by using a diverse set of readily available acrylic acids and (*R*)-α-aminophosphinic acids, and high yields were achieved in all cases. In most examples reported herein, the isolation of biologically relevant (*R*,*S*)-diastereoisomers became possible by simple crystallization from the crude products, thus enhancing the operational simplicity of the proposed method. Finally, functional groups corresponding to acidic or basic natural amino acids are also compatible with the reaction conditions. Based on the above, we expect that the practicality of the proposed protocol will facilitate the discovery of pharmacologically useful bioactive phosphinic peptides.

## 1. Introduction

Among the plethora of bioactive scaffolds with numerous applications in drug discovery, phosphinic peptides undoubtedly hold a prominent position [1,2,3,4,5,6,7,8,9]. By acting as transition state analogues of natural peptides during proteolysis, phosphinic peptides have provided a large number of potent inhibitors of zinc and aspartyl proteases over previous decades [2,5,6]. In addition, their propensity to bind tightly to their target has fueled efforts towards the development of enzymatic probes for imaging applications [10,11,12,13,14]. Their synthesis is typically based on building block strategies where side chains of the phosphinopeptidic scaffold are either preinstalled or introduced at a later stage of the synthetic plan by post-modification protocols [3,4]. Therefore, it becomes evident that synthetic methodologies aiming to the fast and reliable production of phosphinic dipeptide building blocks lie at the heart of every medicinal project involving this privileged class of bioactive compounds.

**Scheme 1:**
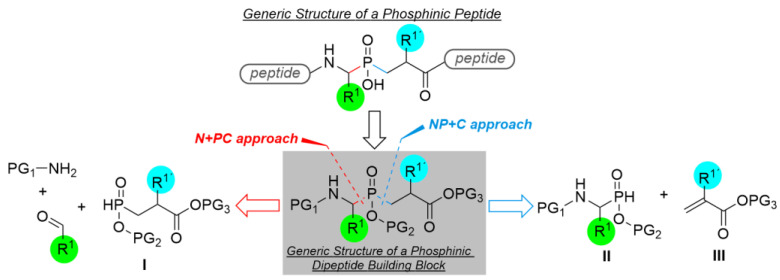
General retrosynthetic approaches for the synthesis of phosphinic peptides.

**Scheme 2:**
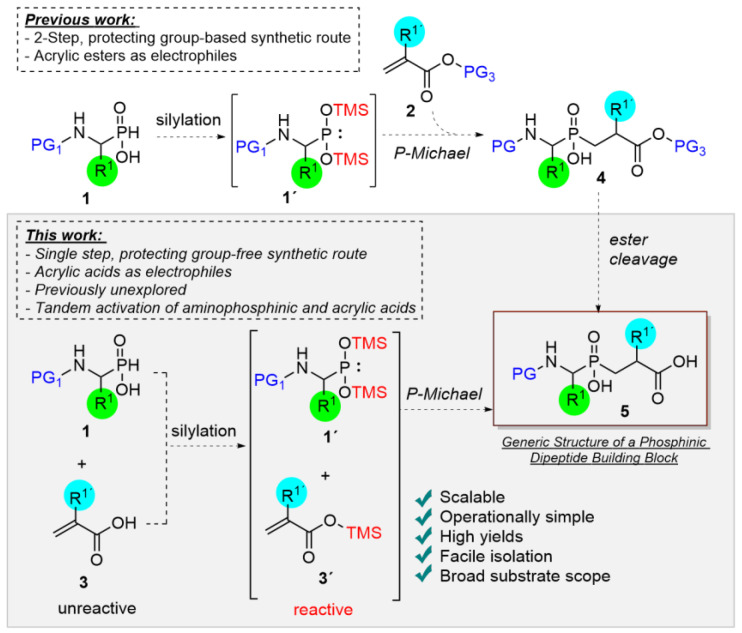
Schematic representation of previously described protocol towards the synthesis of building blocks of type **5** and proposed protocol in this study.

There are two possible synthetic approaches towards a phosphinodipeptidic building block: the “NP + C approach”, which relies on P-C bond-forming reactions between aminophosphinic derivatives and carbon electrophiles, and the less-commonly used “N + PC approach” which involves Mannich-type or amidoalkylation reactions with 3-phosphinoyl propionates of type **I** (Scheme 1) [3,15,16]. The preference of synthetic chemists for the NP + C approach mainly stems from the fact that optically pure α-aminophosphinic acids can be accessed by well-established methods [17,18,19], thus allowing stereochemical efficiency and scalability. In particular, diacids of type **5** (Scheme 2) have been successfully employed as starting materials in the discovery of potent and selective inhibitors of a wide range of proteases, such as ER aminopeptidases [20], neutral aminopeptidase (APN) [21,22], matrix metalloproteinases (MMPs) [23], neprilysin [22], angiotensin-converting enzymes (ACE) 1 and 2 [24,25], endothelin-converting enzyme (ECE) [24] and many more [22,26,27,28]. In most cases, such compounds are produced by the initial conversion of α-aminophosphinic acids of type **1** to the respective bis(trimethylsilyl)phosphonites **1**′, followed by the addition of α-substituted acrylates (**2**) to the resulting *P*-nucleophiles (Scheme 2) [3,29,30]. Since the reaction affords carboxylic esters (**4**), an additional cleavage step is required to furnish the final compounds of type **5** [20,23,31]. Interestingly, a more direct, protecting-group-free approach which involves the use of acrylic acids of type **3** as electrophiles (Scheme 2) has to our knowledge never been reported in the literature, probably because acrylic acids are intuitively excluded when different electrophile alternatives are considered.

Since *P*-Michael additions using phosphinic acids are mainly performed under silylating conditions, we assumed that acrylic acids could also be silylated towards the corresponding esters and react with the *P*-nucleophiles generated in situ (Scheme 2). By extending our literature search to different types of phosphinic acids, only scarce examples of relevant reactions were identified, either leading to moderate yields [32] or employing highly reactive electrophiles [33]. Given this striking gap in the literature, we decided to investigate the feasibility of such a transformation, aiming not only to a shorter synthetic route for the preparation of phosphinic building blocks of type **5**, but also to increased simplicity and scalability. Moreover, there are several examples in the literature where the biologically relevant, less soluble (*R*,*S*)-diastereoisomer of Cbz-protected diacids **5** can be easily separated from the (*R*,*R*)-isomer by crystallization [20,23,25,31,34], which implies that the proposed protocol may have the potential to lead directly to optically pure (*R*,*S*)-phosphinic dipeptides without the need of chromatographic separation. To this respect, in the present study we explore the synthesis of phosphinopeptidic building blocks of type **5** by tandem activation of α-aminophosphinic acids (**1**) and temporary esterification of acrylic acids (**3**) in a *P*-Michael reaction under silylating conditions, aiming to a protocol that will be characterized by high overall practicality and synthetic convenience.

## 2. Results and Discussion

### 2.1. Preparation of Acrylic Acids of Type ***3***

**Scheme 3:**
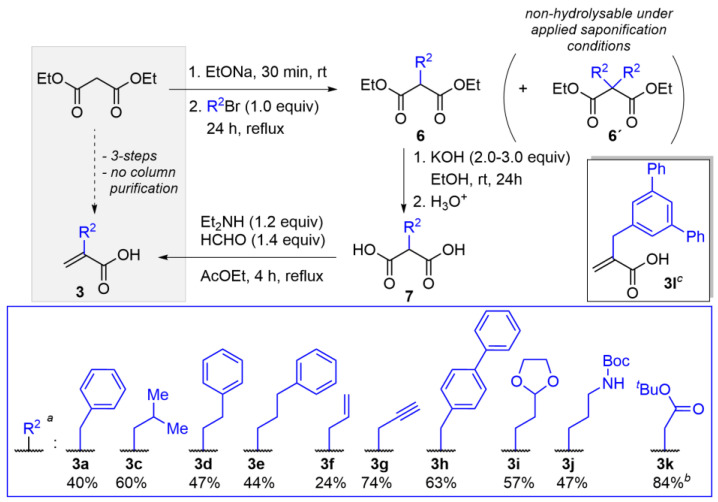
Synthesis of acrylic acids **3a** and **3c**–**k** used in this study (see Supplementary Material). *^a^* Isolated yields are given. *^b^* NaH was used for the alkylation step [35]. *^c^* Prepared according to ref. [20].

Minimization of chromatographic purification steps in a synthetic plan is a crucial parameter that needs to be taken into account for assessing the overall cost efficiency of a preparation, especially when upscaling is required. In this regard, as it was mentioned in the introduction part, simple α-aminophosphinic acids of type **1** can be accessed in multigram quantities and optically pure form by following the classical method of Baylis et al. [19]. On the other hand, α-substituted acrylic acids of type **3** can be accessed by a 3-step procedure starting from diethyl malonate and simple alkyl halides, as described in Scheme 3.

In the first step, the alkylation of diethyl malonate leads to malonic derivatives of type **6**, generally contaminated with small amounts of dialkylated by-products (**6**′) and unreacted materials. The removal of dialkylated derivatives **6**′ becomes possible at the next step by taking advantage of their low reactivity towards hydrolysis due to increased steric hindrance. This allows the facile isolation of the target malonic acids of type **7** by simple aqueous workup. At the final step of the procedure, a Doebner–Knoevenagel condensation with formaldehyde takes place, leading to adequately pure target acrylic acids of type **3** without the need for chromatographic purification and overall yields ranging from 24 to 84% for 3 steps. The structure of acrylic acids of type **3** prepared by the above method is shown in Scheme 3.

### 2.2. Optimization

Next, we proceeded to the evaluation of our proposed method by employing the Cbz-protected aminophosphinic acid **1a** and acrylic acid **3a** as starting materials in our initial experiments (Table 1).

Based on previous reports [32,33], we first tested the efficiency of BSA [*N*,*O*-bis(trimethylsilyl)acetamide] in promoting the *P*-Michael reaction between **1a** and **3a**. The reaction of a mixture of **1a** (1.0 equiv), **3a** (1.2 equiv) and BSA (4 equiv) for 48 h led to just a 53% conversion of the starting material and 41% formation of target compound **5a**, as it was judged by the ^31^P-NMR spectrum of the crude mixture. This was in accordance with the low efficiency observed in the literature for a similar reaction [32]. When unsubstituted acrylic acid (**3a**’) was used, the conversion to product **5a**’ increased to 90% under the same conditions, which implies that steric effects due to the presence of a substituent in 2-position of acrylic acids are partially responsible for the low reactivity of studied electrophiles. TMSCl as silylating agent in the presence of *Hünig*’s base proved more efficient for the formation of product **5a** (82%), based on ^31^P-NMR, leaving only 13% of unreacted **1a**. Despite the improved reaction profile, long reaction times associated with these conditions prompted us to explore hexamethyldisilazane (HMDS) as an alternative, which is known to cause silylation of acidic functionalities upon heating [29]. Indeed, heating of a mixture of **1a**, **3a** and 6 equiv of HMDS to 100 °C during 2 h under inert conditions led to quantitative formation of the desired product **5a**. Lowering the temperature to 70 °C had a negative effect to the conversion of phosphinic acid **1a**, presumably due to incomplete activation of the reactants.

### 2.3. Substrate Scope

**Scheme 4:**
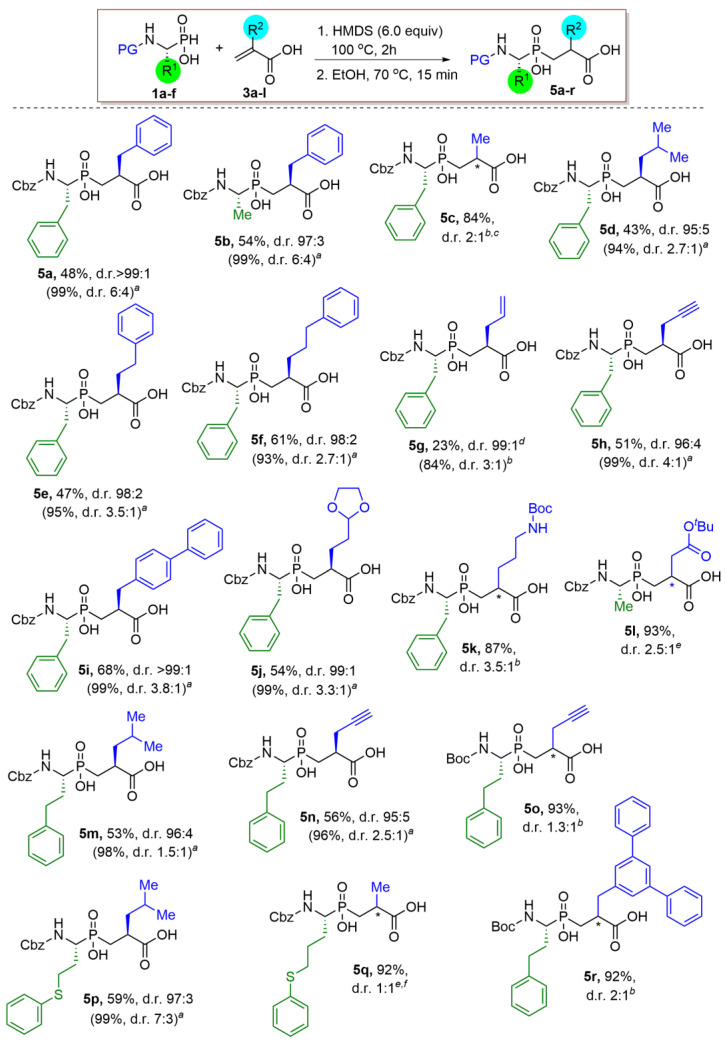
Substrate scope for the proposed *P*-Michael reaction of aminophosphinic (**1**) and acrylic (**3**) acids by tandem esterification under silylating conditions. Isolated yields are provided in all examples. *^a^* outside parentheses: isolated yields to **5** and d.r. values after separation of (*R*,*S*)-isomer by crystallization; inside parentheses: conversions to **5** and d.r. values from crude products, as estimated by integration of their ^31^P-NMR spectra; *^b^* isolated yield after column purification; *^c^* d.r. was estimated by integration of ^31^P-NMR spectrum in CD_3_OD; *^d^* recrystallized by CHCl_3_; *^e^* isolated yield after treatment with Et_2_O; *^f^* d.r. was estimated by integration of the ^31^C-NMR spectrum of isolated product **5q**.

After identifying the optimal conditions for the *P*-Michael addition of α-aminophoshinic acids to acrylic acids through their tandem esterification by silylation, we proceeded to the application of our protocol in a wide range of substrates, as shown in Scheme 4.

Since (*R*)-α-aminophosphinic acids were used, a mixture of two diastereoisomers [(*R*,*S*) and (*R*,*R*)] was obtained in all cases. However, as mentioned above, the (*R*,*S*)-diastereoisomer of Cbz-protected diacids of type **5** has a higher tendency to crystallize compared to (*R*,*R*) isomers. This was observed in 12 out of 16 examples of Cbz-protected diacids prepared in this study, allowing the isolation of target compounds of type **5** with high diastereoisomeric purity, as determined by ^31^P-NMR, *without the need of chromatographic purification*. Considering that starting materials were also obtained without column chromatography, it becomes evident that the proposed protocol is characterized by high practicality and scalability. Boc-protected α-aminophosphinic acids are also compatible with this protocol leading to compounds **5o** and **5r** in high yields. However, in these cases the presence of Boc group hampered diastereoisomeric separation. Furthermore, acrylic acids with functional groups corresponding to the side chains of acidic (**5l**) or basic (**5k**) aminoacids were also compatible with the proposed method.

## 3. Materials and Methods

### 3.1. General Information

All *P*-Michael reactions were carried out under argon atmosphere. All solvents were used without further purification. Reagents were purchased at the highest commercial quality from Aldrich (St. Louis, MI, USA), Acros (Belgium), Fluka and Fluorochem and were used without further purification. Reactions were monitored by thin-layer chromatography (TLC) carried out on 0.25 mm silica gel plates (E. Merck silica gel 60F_254_) and components were visualized by the following methods: UV light absorbance, and/or charring after spraying with a solution of NH_4_HSO_4_ or an aqueous solution of cerium molybdate/H_2_SO_4_ (“Blue Stain”), or an aqueous solution of KMnO_4_ and heating. Purification of compounds by column chromatography was carried out on silica gel (Merck (Darmstadt, Germany), 70–230 mesh) and the indicated solvents. Melting points (measured on an Electrothermal apparatus) are uncorrected. ^1^H, ^13^C and ^31^P NMR spectra were recorded on a Varian 200 MHz Mercury spectrometer and on a Bruker Avance Neo 400 MHz at 25 °C. ^1^H and ^13^C spectra are referenced according to the residual peak of the solvent based on literature data [36]. ^31^P NMR chemical shifts are reported in ppm downfield from 85% H_3_PO_4_ (external standard). ^13^C and ^31^P NMR spectra are fully proton-decoupled. Phosphinic diacids of type **5** tend to aggregate, causing peak broadening in the NMR spectra. In all products of type **5**, small signals observed upfield from the main peaks corresponded to rotamers observed in this type of compound, as it has been reported in the literature [37]. Signals marked with an asterisk (*) in the NMR assignment correspond to minor isomers. Optical rotation data were obtained in an Optical Activity instrument. High-resolution mass spectra were obtained on a Bruker Maxis Impact QTOF spectrometer or an AB Sciex 4600 Triple TOF mass spectrometer.

### 3.2. General Procedure for the HMDS-Mediated P-C Bond-Forming Reaction between Phosphinic and Acrylic Acids

Phosphinic acid of type **1** (1.0 equiv) and acrylic acid of type **3** (1.2 equiv) were added to a round-bottomed flask. Then, HMDS (6.0 equiv) was slowly added and the flask was firmly closed with a septum and thoroughly purged with argon. Then, the mixture was slowly heated to 100 °C, ensuring that gas NH_3_ was released (by periodically piercing the septum with a needle, taking care to always maintain a positive pressure inside the flask). Production of gas NH_3_ starts at ~60 °C and was completed at 100 °C. The mixture was heated at 100 °C during 2 h and then cooled at 70 °C. At this temperature, EtOH (0.2 mL/mmol of HMDS) was slowly added under a gentle flow of argon, and stirring was continued for 15 min. The resulting mixture was diluted with HCl 2M (15 mL/mmol of **1**) and AcOEt (20 mL/mmol of **1**). Isolation of the final product of type **5** is described separately for each example given below.

*(2S)-2-Benzyl-3-{[(R)-1-(benzyloxycarbonylamino)-2-henylethyl](hydroxy)phosphoryl}propanoic acid* (**5a**): Phosphinic acid **1a** (100 mg, 0.31 mmol) and acrylic acid **3a** (61 mg, 0.37 mmol) were subjected to the general HMDS-mediated P-C bond-forming reaction protocol. After the addition of HCl 2 M and AcOEt, a solid was precipitated that was collected by filtration. The solid product was recrystallized by AcOEt to afford compound **5a** (d.r. >99:1) as a white solid in 48% yield (72 mg, 0.15 mmol). m.p. 190–195 °C; TLC *R*_f_ (CHCl_3_/MeOH/AcOH: 7/2/1) = 0.58. ^1^H NMR (500 MHz, d_6_-DMSO + 4%TFA) *δ* 7.68 (d, *J* = 9.5 Hz, 1H), 7.37–6.94 (m, 15H), 4.95 (d, *J* = 13 Hz, 1H), 4.88 (d, *J* = 13 Hz, 1H), 3.14–2.84 (m, 4H), 2.78–2.68 (m, 1H), 2.10–1.92 (m, 1H), 1.84–1.74 (m, 1H); ^13^C NMR (50 MHz, d_6_-DMSO) *δ* 176.5 (d, ^3^*J*_PC_ = 7.6 Hz), 156.1 (d, ^3^*J*_PC_ = 3.8 Hz), 138.9, 138.6, 138.4, 137.3, 129.2, 128.4, 127.7, 127.1, 126.4, 65.3, 52.7 (d, ^1^*J*_PC_ = 104 Hz), 40.5, 38.5, 32.8, 27.6 (d, ^1^*J*_PC_ = 89 Hz); ^31^P NMR (81 MHz, d_6_-DMSO + 4%TFA) *δ* 46.4; HRMS (*m/z*): [M + Na]^+^ calcd. for C_26_H_28_NNaO_6_P^+^, 504.1547 found, 504.1565.

*(2S)-2-Benzyl-3-{[(R)-1-(benzyloxycarbonylamino)ethyl](hydroxy)phosphoryl}propanoic acid* (**5b**): Phosphinic acid **1b** (100 mg, 0.41 mmol) and acrylic acid **3a** (80 mg, 0.49 mmol) were subjected to the general HMDS-mediated P-C bond-forming reaction protocol. After the addition of HCl 2M and AcOEt, a solid was precipitated that was collected by filtration. The solid product was recrystallized by AcOEt to afford compound **5b** (d.r. >97:3) as a white solid in 54% yield (90 mg, 0.22 mmol). m.p. 180–185 °C; TLC *R*_f_ (CHCl_3_/MeOH/AcOH: 7/2/1) = 0.33. ^1^H NMR (200 MHz, d_6_-DMSO + 4.5%TFA) *δ* 7.52 (d, *J* = 9.0 Hz, 1H), 7.39–7.13 (m, 10H), 5.11–4.96 (m, 2H), 3.85–3.72 (m, 1H), 3.00–2.79 (m, 3H), 2.04–1.94 (m, 1H), 1.78–1.61 (m, 1H), 1.20 (dd, *J* = 14.2, 7.3 Hz, 3H); ^13^C NMR (50 MHz, d_6_-DMSO + 4.5%TFA) *δ* 175.4 (d, ^3^*J*_PC_ = 9.4 Hz), 155.9 (d, ^3^*J*_PC_ = 4.1 Hz), 138.9, 137.1, 129.1, 128.5, 128.3, 127.9, 127.8, 126.4, 65.7, 46.2 (d, ^1^*J*_PC_ = 105 Hz), 40.53, 40.45, 38.5, 27.3 (d, ^1^*J*_PC_ = 88 Hz), 13.9; ^31^P NMR (81 MHz, d_6_-DMSO + 4.5%TFA) *δ* 47.6; HRMS (*m/z*): [M + H]^+^ calcd. for C_20_H_25_NO_6_P^+^, 406.1414 found, 406.1424.

*3-{[(R)-1-(Benzyloxycarbonylamino)-2-phenylethyl](hydroxy)phosphoryl}-2-methylpropanoic acid* (**5c**): Phosphinic acid **1a** (250 mg, 0.78 mmol) and methacrylic acid (**3b**) (81 mg, 0.94 mmol) were subjected to the general HMDS-mediated P-C bond-forming reaction protocol. After the addition of HCl 2M and AcOEt, the two phases were separated and the organic layer was dried with Na_2_SO_4_ and evaporated in vacuo. The crude product was purified by silica gel column chromatography using CHCl_3_/MeOH/AcOH 7:0.01:0:01 → 7:0.4:0:4 as eluent solvent system. Compound **5c** (d.r. 2:1) was isolated as a white solid in 84% yield (266 mg, 0.65 mmol). TLC *R*_f_ (CHCl_3_/MeOH/AcOH: 7/2/1) = 0.39. ^1^H NMR (200 MHz, d_6_-DMSO) *δ* 7.72 & 7.71 (2 × d, *J* = 9.5 Hz, 1H), 7.36–7.07 (m, 10H), 4.92 (s, 2H), 3.97–3.73 (m, 1H), 3.18–2.94 (m, 1H), 2.84–2.57 (m, 2H), 2.19–1.93 (m, 1H), 1.73–1.45 (m, 1H), 1.19 & 1.17 (2 × d, *J* = 7.0 Hz, 3H); ^13^C NMR (50 MHz, d_6_-DMSO) *δ* 176.90, 176.86, 176.7, 156.1, 156.0, 138.8, 138.7, 138.5, 138.44, 137.37, 137.3, 129.1, 128.4, 128.2, 127.6, 127.1, 127.0, 126.2, 65.1, 56.6 (d, ^1^*J*_PC_ = 104 Hz), 52.2* (d, ^1^*J*_PC_ = 105 Hz), 33.4, 33.35, 33.28, 33.2, 32.7, 30.6, 30.4, 28.88, 28.68, 19.0 (d, ^1^*J*_PC_ = 7.4 Hz), 18.7 (d, ^1^*J*_PC_ = 6.0 Hz); ^31^P NMR (81 MHz, d_6_-DMSO + 5%TFA) *δ* 46.5; HRMS (*m/z*): [M − H]^−^ calcd. for C_20_H_23_NO_6_P^−^, 404.1268 found, 404.1264.

*(2S)-2-({[(R)-1-(Benzyloxycarbonylamino)-2-phenylethyl](hydroxy)phosphoryl}methyl)-4-methylpentanoic acid* (**5d**): Phosphinic acid **1a** (100 mg, 0.31 mmol) and acrylic acid **3c** (48 mg, 0.37 mmol) were subjected to the general HMDS-mediated P-C bond-forming reaction protocol. After the addition of HCl 2M and AcOEt, the two phases are separated and the organic layer was dried with Na_2_SO_4_ and evaporated in vacuo. The residue was recrystallized twice with AcOEt to afford compound **5d** (d.r. 95:5) as a white solid in 43% yield (60 mg, 0.13 mmol). m.p. 153–156 °C; [a]_D_^25^ = −32 (c = 0.31, DMSO); TLC *R*_f_ (CHCl_3_/MeOH/AcOH: 7/2/1) = 0.6. ^1^H NMR (200 MHz, d_6_-DMSO) *δ* 7.66 (d, *J* = 9.5 Hz, 1H), 7.39–6.88 (m, 10H), 5.02–4.78 (m, 2H), 4.02–3.73 (m, 1H), 3.17–2.97 (m, 1H), 2.82–2.57 (m, 2H), 2.11–1.86 (m, 1H), 1.78–1.26 (m, 4H), 0.86 (app t, *J* = 6.3 Hz, 6H); ^13^C NMR (50 MHz, d_6_-DMSO) *δ* 176.5 (d, ^3^*J*_PC_ = 7.6 Hz), 156.1 (d, ^3^*J*_PC_ = 3.8 Hz), 138.6, 138.4, 137.3, 129.1, 128.4, 128.2, 127.6, 127.1, 126.3, 65.1, 52.8 (d, ^1^*J*_PC_ = 104 Hz), 42.7 (d, *J*_PC_ = 9.0 Hz), 36.8 (d, *J*_PC_ = 4.0 Hz), 32.8, 28.9 (d, ^1^*J*_PC_ = 88 Hz), 25.60, 23.01, 21.87; ^31^P NMR (81 MHz, d_6_-DMSO) *δ* 45.7*, 45.4; HRMS (m/z): [M − H]^−^ calcd. for C_23_H_29_NO_6_P^−^, 446.1738 found, 446.1739.

*(2S)-2-({[(R)-1-(Benzyloxycarbonylamino)-2-phenylethyl](hydroxy)phosphoryl}methyl)-4-phenylbutanoic acid* (**5e**): Phosphinic acid **1a** (200 mg, 0.63 mmol) and acrylic acid **3d** (133 mg, 0.76 mmol) were subjected to the general HMDS-mediated P-C bond-forming reaction protocol. After the addition of HCl 2M and AcOEt, a solid was precipitated that was collected by filtration. The solid product was recrystallized by AcOEt to afford compound **5e** (d.r. 98:2) as a white solid in 47% yield (147 mg, 0.30 mmol). m.p. 196–199 °C; [a]_D_^25^ = −31.2 (c = 0.9, DMSO); TLC *R*_f_ (CHCl_3_/MeOH/AcOH: 7/2/1) = 0.62. ^1^H NMR (200 MHz, d_6_-DMSO) *δ* 7.71 (d, *J* = 9.7 Hz, 1H), 7.40–6.90 (m, 15H), 4.92 (s, 2H), 3.98–3.73 (m, 1H), 3.20–2.95 (m, 1H), 2.82–2.41 (m, 4H, overlapped by d_6_-DMSO), 2.17–1.62 (m, 4H); ^13^C NMR (50 MHz, d_6_-DMSO) *δ* 175.9 (d, ^3^*J*_PC_ = 9.0 Hz), 156.1 (d, ^3^*J*_PC_ = 3.7 Hz), 141.5, 138.6, 138.4, 137.3, 129.1, 128.4, 128.3, 128.2, 127.6, 127.1, 126.3, 125.9, 65.1, 52.7 (d, ^1^*J*_PC_ = 104 Hz), 38.4 (d, *J*_PC_ = 4.2 Hz), 34.8 (d, *J*_PC_ = 8.0 Hz), 32.8, 32.5, 28.1 (d, ^1^*J*_PC_ = 89 Hz); ^31^P NMR (81 MHz, d_6_-DMSO + 2% TFA) *δ* 46.2; HRMS (*m/z*): [M − H]^−^ calcd. for C_24_H_29_NO_6_P^−^, 494.1738 found, 494.1744.

*(2S)-2-({[(R)-1-(Benzyloxycarbonylamino)-2-phenylethyl](hydroxy)phosphoryl}methyl)-5-phenylpentanoic acid* (**5f**): Phosphinic acid **1a** (200 mg, 0.63 mmol) and acrylic acid **3e** (143 mg, 0.75 mmol) were subjected to the general HMDS-mediated P-C bond-forming reaction protocol. After the addition of HCl 2M and AcOEt, the two phases were separated and the organic layer was dried with Na_2_SO_4_ and evaporated in vacuo. The residue was recrystallized twice with AcOEt to afford compound **5f** (d.r. 98:2) as a white solid in 61% yield (196 mg, 0.38 mmol). m.p. 183–186 °C; [a]_D_^25^ = −22.0 (c = 1, AcOH); TLC *R*_f_ (CHCl_3_/MeOH/AcOH: 7/2/1) = 0.60. ^1^H NMR (200 MHz, d_6_-DMSO) *δ* 7.71 (d, *J* = 9.0 Hz, 1H), 7.44–6.96 (m, 15H), 4.98–4.72 (m, 2H), 3.95–3.72 (m, 1H), 3.14–2.96 (m, 1H), 2.80–2.33 (m, 4H, overlapped by d_6_-DMSO), 2.10–1.81 (m, 1H), 1.79–1.35 (m, 5H); ^13^C NMR (50 MHz, d_6_-DMSO) *δ* 176.0 (d, ^3^*J*_PC_ = 7.3 Hz), 156.0 (d, ^3^*J*_PC_ = 3.6 Hz), 142.0, 138.7, 138.4, 137.3, 129.0, 128.3, 128.2, 127.5, 126.9, 126.2, 125.7, 65.0, 52.8 (d, ^1^*J*_PC_ = 104 Hz), 35.0, 33.0, 32.8, 28.6, 28.5 (d, ^1^*J*_PC_ = 104 Hz); ^31^P NMR (81 MHz, d_6_-DMSO) *δ* 45.0; HRMS (*m/z*): [M − H]^−^ calcd. for C_2__8_H_31_NO_6_P^−^, 508.1894 found, 508.1889.

*(2S)-2-({[(R)-1-(Benzyloxycarbonylamino)-2-phenylethyl](hydroxy)phosphoryl}methyl)pent-4-enoic acid* (**5g**): Phosphinic acid **1a** (200 mg, 0.63 mmol) and acrylic acid **3f** (84 mg, 0.75 mmol) were subjected to the general HMDS-mediated P-C bond-forming reaction protocol. After the addition of HCl 2M and AcOEt, the two phases were separated and the organic layer was dried with Na_2_SO_4_ and evaporated in vacuo. The crude product was purified by silica gel column chromatography using CHCl_3_/MeOH/AcOH 7:0.01:0:01 → 7:0.4:0:4 as eluent solvent system. Compound **5g** was isolated as an off-white solid in 94% yield (255 mg, 0.59 mmol). Isolation of diastereoisomer (*R*,*S*)-**5g** (d.r. 99:1) was achieved after 3 recrystallizations with CHCl_3_ in 23% yield (62 mg, 0.14 mmol). m.p. 131–133 °C; [a]_D_^25^ = −32.8 (c = 1, MeOH); TLC *R*_f_ (CHCl_3_/MeOH/AcOH: 7/2/1) = 0.44. ^1^H NMR (200 MHz, d_6_-DMSO) *δ* 7.70 (d, *J* = 9.2 Hz, 1H), 7.39–7.03 (m, 10H), 5.81–5.55 (m, 1H), 5.11–4.96 (m, 2H), 4.92 (s, 2H), 3.98–3.74 (m, 1H), 3.15–2.94 (m, 1H), 2.86–2.59 (m, 2H), 2.42–2.23 (m, 2H), 2.10–1.87 (m, 1H), 1.83–1.59 (m, 1H); ^13^C NMR (50 MHz, d_6_-DMSO) *δ* 175.4 (d, ^3^*J*_PC_ = 8.9 Hz), 156.1 (d, ^3^*J*_PC_ = 3.8 Hz), 138.6, 138.4, 137.3, 135.1, 129.0, 128.3, 128.2, 127.6, 127.1, 126.2, 117.5, 52.6 (d, ^1^*J*_PC_ = 105 Hz), 36.8 (d, *J*_PC_ = 7.7 Hz), 32.7, 27.3 (d, ^1^*J*_PC_ = 89 Hz); ^31^P NMR (81 MHz, d_6_-DMSO) *δ* 45.7; HRMS (*m/z*): [M − H]^−^ calcd. for C_23_H_2__5_NO_6_P^−^, 430.1425 found, 430.1437.

*(2S)-2-({[(R)-1-(Benzyloxycarbonylamino)-2-phenylethyl](hydroxy)phosphoryl}methyl)pent-4-ynoic acid* (**5h**): Phosphinic acid **1a** (410 g, 1.3 mmol) and acrylic acid **3g** (170 mg, 1.54 mmol) were subjected to the general HMDS-mediated P-C bond-forming reaction protocol. After the addition of HCl 2M and AcOEt, a solid was precipitated that was collected by filtration. The solid product was refluxed with AcOEt and the hot suspension is filtrated. The procedure is repeated to afford compound **5h** (d.r. 96:4) as a white solid in 51% yield (284 mg, 0.66 mmol). m.p. 171–174 °C; [a]_D_^25^ = −43 (c = 1, DMSO); TLC *R*_f_ (CHCl_3_/MeOH/AcOH: 7/2/1) = 0.59. ^1^H NMR (400 MHz, d_6_-DMSO) *δ* 7.70 (d, *J* = 9.4 Hz, 1H), 7.36–6.94 (m, 10H), 4.95 (d, *J* = 13 Hz, 1H), 4.90 (d, *J* = 13 Hz, 1H), 4.01–3.84 (m, 1H), 3.17–3.02 (m, 2H), 2.92–2.51 (m, 5H), 2.20–2.04 (m, 1H), 1.96–1.81 (m, 1H); ^13^C NMR (101 MHz, d_6_-DMSO) *δ* 174.3 (d, ^3^*J*_PC_ = 11.7 Hz), 156.1 (d, ^3^*J*_PC_ = 3.5 Hz), 138.5, 138.3, 137.2, 129.0, 128.3, 128.2, 127.6, 127.1, 126.7, 126.2, 81.4, 72.9, 65.2, 52.3 (d, ^1^*J*_PC_ = 105 Hz), 37.8, 32.7, 26.7 (d, ^1^*J*_PC_ = 89 Hz), 21.5 (d, ^1^*J*_PC_ = 4.9 Hz); ^31^P NMR (162 MHz, d_6_-DMSO) *δ* 45.0; HRMS (*m/z*): [M − H]^−^ calcd. for C_23_H_2__3_NO_6_P^−^, 428.1268 found, 428.1262.

*(2S)-3-{[1,1′-Biphenyl]-4-yl}-2-({[(R)-1-(benzyloxycarbonylamino)-2-phenylethyl](hydroxy)phosphoryl}methyl)propanoic acid* (**5i**): Phosphinic acid **1a** (200 mg, 0.63 mmol) and acrylic acid **3h** (180 mg, 0.76 mmol) were subjected to the general HMDS-mediated P-C bond-forming reaction protocol. After the addition of HCl 2M and AcOEt, a solid was precipitated that was collected by filtration. The solid product was refluxed with AcOEt and the hot suspension is filtrated. The procedure was repeated to afford compound **5i** (d.r. >99:1) as a white solid in 68% yield (239 g, 0.43 mmol). m.p. 208–210 °C; [a]_D_^25^ = −23.8 (c = 0.95, DMSO); ^1^H NMR (200 MHz, d_6_-DMSO + 4%TFA) *δ* 7.77–6.95 (m, 20H), 5.02–4.74 (m, 2H), 4.03–3.77 (m, 1H), 3.16–2.82 (m, 4H), 2.81–2.59 (m, 1H), 2.19–1.93 (m, 1H), 1.92–1.67 (m, 1H); ^13^C NMR (50 MHz, d_6_-DMSO) *δ* 175.3 (d, ^3^*J*_PC_ = 9.6 Hz), 156.0 (d, ^3^*J*_PC_ = 3.8 Hz), 140.0, 138.6, 138.3, 138.2, 137.2, 129.7, 129.0, 129.0, 128.3, 128.2, 127.6, 127.3, 127.0, 126.6, 126.2, 65.1, 52.6, (d, ^1^*J*_PC_ = 105 Hz), 32.71, 27.7 (d, ^1^*J*_PC_ = 89 Hz); ^31^P NMR (81 MHz, d_6_-DMSO + 4%TFA) *δ* 46.6; HRMS (*m/z*): [M + Na]^+^ calcd. for C_32_H_31_NO_6_P^−^, 556.1894 found, 556.1896.

*(2S)-2-({[(R)-1-(Benzyloxycarbonylamino)-2-phenylethyl](hydroxy)phosphoryl}methyl)-4-(1,3-dioxolan-2-yl)butanoic acid* (**5j**): Phosphinic acid **1a** (200 mg, 0.63 mmol) and acrylic acid **3i** (130 mg, 0.76 mmol) were subjected to the general HMDS-mediated P-C bond-forming reaction protocol. After the addition of HCl 2M and AcOEt, a solid was precipitated that was collected by filtration. The solid product was recrystallized by AcOEt to afford compound **5j** (d.r. 99:1) as a white solid in 54% yield (167 mg, 0.34 mmol). m.p. 143–146 °C; [a]_D_^25^ = −31.0 (c = 0.9, MeOH); TLC *R*_f_ (CHCl_3_/MeOH/AcOH: 7/2/1) = 0.34. ^1^H NMR (200 MHz, d_6_-DMSO) *δ* 7.69 (d, *J* = 9.4 Hz, 1H), 7.39–6.92 (m, 10H), 4.92 (s, 2H), 4.79–4.70 (m, 1H), 3.96–3.67 (m, 5H), 3.14–2.97 (m, 1H), 2.81–2.56 (m, 2H), 2.12–1.88 (m, 1H), 1.81–1.43 (m, 5H); ^13^C NMR (50 MHz, d_6_-DMSO) *δ* 175.9 (d, ^3^*J*_PC_ = 8.3 Hz), 156.1 (d, ^3^*J*_PC_ = 3.7 Hz), 138.6, 138.4, 137.3, 129.0, 128.3, 128.2, 127.6, 127.1, 126.3, 103.3, 65.1, 64.3, 52.7 (d, ^1^*J*_PC_ = 105 Hz), 38.4 (d, *J*_PC_ = 3.3 Hz), 32.8, 30.9, 28.2 (d, ^1^*J*_PC_ = 88 Hz), 27.5 (d, *J*_PC_ = 7.5 Hz); ^31^P NMR (81 MHz, d_6_-DMSO) *δ* 45.6; HRMS (m/z): [M − H]^−^ calcd. for C_24_H_29_NO_6_P^−^, 490.1636 found, 490.1638.

*(2S)-2-({[(R)-1-(Benzyloxycarbonylamino)-2-phenylethyl](hydroxy)phosphoryl}methyl)-5-[(tert-butoxycarbonyl)amino]pentanoic acid* (**5k**): Phosphinic acid **1a** (300 mg, 0.94 mmol) and acrylic acid **3j** (259 mg, 1.12 mmol) were subjected to the general HMDS-mediated P-C bond-forming reaction protocol. After the addition of HCl 2M and AcOEt, the two phases were separated and the organic layer was dried with Na_2_SO_4_ and evaporated in vacuo. The crude product was purified by silica gel column chromatography using CHCl_3_/MeOH/AcOH 7:0.01:0:01 → 7:0.4:0:4 as eluent solvent system. Compound **5k** (d.r. 3.5:1) was isolated as a white foam in 87% yield (449 mg, 0.82 mmol). TLC R_f_ (CHCl_3_/MeOH/AcOH: 7/2/1) = 0.72. ^1^H NMR (200 MHz, d_6_-DMSO) δ 7.63 (d, J = 9.5 Hz, 1H), 7.35–6.70 (m, 10H), 4.93 (d, J = 13 Hz, 1H), 4.84 (d, J = 13 Hz, 1H), 3.93–3.69 (m, 1H), 3.12–2.93 (m, 1H), 2.93–2.52 (m, 5H, overlapped by d_6_-DMSO), 2.15–1.82 (m, 1H), 1.74–1.14 (m, 13H); ^13^C NMR (50 MHz, d_6_-DMSO/CDCl_3_ 1:1) δ 176.0 (d, ^3^J_PC_ = 8.1 Hz), 156.0 (d, ^3^J_PC_ = 3.6 Hz), 155.6, 138.6, 138.3, 137.2, 129.0, 128.3, 128.1, 127.5, 127.0, 126.1, 65.1, 52.6 (d, ^1^J_PC_ = 105 Hz), 32.8, 30.6, 30.5, 29.1, 28.8*, 28.7*, 28.3, 28.0*, 27.2; ^31^P NMR (81 MHz, d_6_-DMSO) δ 46.3*, 45.9; HRMS (*m/z*): [M − H]^−^ calcd. for C_2__7_H_36_N_2_O_8_P^−^, 547.2215 found, 547.2217.

*2-({[(R)-1-(Benzyloxycarbonylamino)ethyl](hydroxy)phosphoryl}methyl)-4-(tert-butoxy)-4-oxo-2λ^3^-butanoic acid* (**5l**): Phosphinic acid **1b** (500 mg, 1.57 mmol) and acrylic acid **3k** (48 mg, 0.37 mmol) were subjected to the general HMDS-mediated P-C bond-forming reaction protocol. After the addition of HCl 2M and AcOEt, the two phases were separated and the organic layer was dried with Na_2_SO_4_ and evaporated in vacuo. The residue was treated with Et_2_O, filtrated and washed with Et_2_O. Compound **5l** (d.r. 2.5:1) was isolated as a white solid in 93% yield (129 mg, 0.29 mmol). TLC *R*_f_ (CHCl_3_/MeOH/AcOH: 7/2/1) = 0.28. ^1^H NMR (200 MHz, d_6_-DMSO) *δ* {[7.58 (d, *J* = 9.0 Hz) & 7.56* (d, *J* = 9.0 Hz)], 1H}, 7.41–7.25 (m, 5H), 5.11–4.95 (m, 2H), 3.87–3.62 (m, 1H), 3.04–2.81 (m, 1H), 2.80–2.63 (m, 1H), 2.58–2.38 (m, 1H, overlapped by d_6_-DMSO), 2.13–1.92 (m, 1H), 1.83–1.60 (m, 1H), 1.37 (s, 9H), 1.20 (dd, *J* = 14.4, 7.4 Hz, 3H); ^13^C NMR (50 MHz, d_6_-DMSO) *δ* 175.3 (d, ^3^*J*_PC_ = 13.5 Hz), 170.7, 156.0 (d, ^3^*J*_PC_ = 4.9 Hz), 128.5, 128.0, 127.84, 127.79, 80.0, 65.8, 46.3 (d, ^1^*J*_PC_ = 105 Hz)*, 46.1 (d, ^1^*J*_PC_ = 107 Hz), 37.18*, 37.16*, 36.93, 36.87, 35.0*, 34.9*, 35.2, 35.1, 27.8, 26.0, 13.9; ^31^P NMR (81 MHz, d_6_-DMSO) *δ* 47.3, 46.9; HRMS (*m/z*): [M − H]^−^ calcd. for C_19_H_27_NO_8_P^−^, 428.1480 found, 428.1467.

*(2S)-2-((((R)-1-(((Benzyloxy)carbonyl)amino)-3-phenylpropyl)(hydroxy)phosphoryl)methyl)-4-methylpentanoic acid* (**5m**): Phosphinic acid **1c** (1.0 g, 3.0 mmol) and acrylic acid **3c** (461 mg, 3.6 mmol) were subjected to the general HMDS-mediated P-C bond-forming reaction protocol. After the addition of HCl 2M and AcOEt, a solid was precipitated that was collected by filtration. The solid product was refluxed with AcOEt and the hot suspension was filtrated to afford compound **5m** (d.r. 96:4) as a white solid in 53% yield (733 mg, 1.59 mmol). m.p. 184–186 °C; [a]_D_^25^ = −19 (c = 1, DMSO); TLC *R*_f_ (CHCl_3_/MeOH/AcOH: 7/2/1) = 0.7. ^1^H NMR (400 MHz, d_6_-DMSO + 20%CD_3_OD) *δ* 7.57 (d, *J* = 9.2 Hz, 0.2H partially exchanged), 7.41–7.08 (m, 10H), 5.08 (s, 2H), 3.68–3.54 (m, 1H), 2.77–2.57 (m, 2H), 2.55–2.42 (m, 1H, overlapped by d_6_-DMSO), 2.03–1.86 (m, 2H), 1.85–1.72 (m, 1H), 1.67–1.56 (m, 1H), 1.55–1.28 (m, 3H), 0.85 & 0.82 (2 × d, *J* = 6.4 Hz, 6H); ^13^C NMR (101 MHz, d_6_-DMSO + 20%CD_3_OD) *δ* 176.4 (d, ^3^*J*_PC_ = 7.8 Hz), 156.4 (d, ^3^*J*_PC_ = 3.8 Hz), 141.3, 137.3, 128.5, 128.4, 128.3, 127.9, 127.7, 125.9, 65.6, 50.3 (d, ^1^*J*_PC_ = 105 Hz), 42.6 (d, *J*_PC_ = 8.5 Hz), 36.7 (d, *J*_PC_ = 3.9 Hz), 31.7 (d, *J*_PC_ = 12 Hz), 29.3, 28.8 (d, ^1^*J*_PC_ = 89 Hz), 25.6, 22.9, 21.8; ^31^P NMR (162 MHz, d_6_-DMSO + 20%CD_3_OD) *δ* 45.0; HRMS (*m/z*): [M + H]^+^ calcd. for C_2__4_H_33_NO_6_P^+^, 462.2040 found, 462.2046.

*(2S)-2-({[(R)-1-(Benzyloxycarbonylamino)-3-phenylpropyl](hydroxy)phosphoryl}methyl)pent-4-ynoic acid* (**5n**): Phosphinic acid **1c** (1.5 g, 4.5 mmol) and acrylic acid **3g** (594 mg, 5.4 mmol) were subjected to the general HMDS-mediated P-C bond-forming reaction protocol. After the addition of HCl 2M and AcOEt, a solid was precipitated that was collected by filtration. The solid product was refluxed with AcOEt and the hot suspension was filtrated. The procedure was repeated thrice to afford compound **5n** (d.r. 95:5) as a white solid in 56% yield (1.1 g, 2.5 mmol). m.p. 162–164 °C; [a]_D_^25^ = −22 (c = 1, DMSO); TLC *R*_f_ (CHCl_3_/MeOH/AcOH: 7/0.5/0.5) = 0.16. ^1^H NMR (400 MHz, d_6_-DMSO + 5% TFA) *δ* 7.63 (d, *J* = 9.3 Hz, 1H), 7.42–7.08 (m, 10H), 5.06 (s, 2H), 3.73–3.57 (m, 1H), 2.84–2.61 (m, 2H), 2.62–2.43 (m, 2H, overlapped by d_6_-DMSO), 2.10–1.92 (m, 2H), 1.87–1.72 (m, 2H); ^13^C NMR (50 MHz, d_6_-DMSO) *δ* 174.6 (d, ^3^*J*_PC_ = 11.8 Hz), 156.7 (d, ^3^*J*_PC_ = 3.7 Hz), 141.5, 137.4, 128.7, 128.7, 128.2, 128.0, 126.2, 81.6, 73.1, 66.0, 50.3 (d, ^1^*J*_PC_ = 106 Hz), 37.9, 31.9 (d, *J*_PC_ = 12 Hz), 29.4, 26.7 (d, ^1^*J*_PC_ = 87 Hz), 21.8; ^31^P NMR (162 MHz, d_6_-DMSO + 5% TFA) *δ* 45.7; HRMS (*m/z*): [M + Na]^+^ calcd. for C_23_H_26_NNaO_6_P^+^, 466.1390 found, 456.1368.

*2-[({(R)-1-[(tert-Butoxycarbonyl)amino]-3-phenylpropyl}(hydroxy)phosphoryl)methyl]pent-4-ynoic acid* (**5o**): Phosphinic acid **1d** (450 mg, 1.50 mmol) and acrylic acid **3g** (198 mg, 1.80 mmol) were subjected to the general HMDS-mediated P-C bond-forming reaction protocol. After the addition of HCl 2M and AcOEt, the two phases were separated and the organic layer was dried with Na_2_SO_4_ and evaporated in vacuo. The crude product was purified by silica gel column chromatography using CHCl_3_/MeOH/AcOH 7:0.01:0:01 → 7:0.4:0:4 as eluent solvent system. Compound **5o** (d.r. 1.3:1) was isolated as an off-white solid in 89% yield (547 mg, 1.34 mmol). TLC *R*_f_ (CHCl_3_/MeOH/AcOH: 7/0.5/0.5) = 0.16. ^1^H NMR (400 MHz, d_6_-DMSO) *δ* 7.35–7.07 (m, 6H), 3.62–3.52 (m, 1H), 2.80–2.65 (m, 3H), 2.61–2.50 (m, 2H, overlapped by d_6_-DMSO), 2.06–1.89 (m, 2H), 1.84–1.68 (m, 2H), 1.41 & 1.37 (2 × s, 9H); ^13^C NMR (101 MHz, d_6_-DMSO) *δ* 174.2 (d, ^3^*J*_PC_ = 12 Hz), 155.7, 155.64, 155.61, 141.39, 141.36, 128.5, 128.3, 125.8, 81.4, 78.3*, 78.2, 72.7, 48.2 (d, ^1^*J*_PC_ = 107 Hz), 37.8* (d, *J*_PC_ = 2.7 Hz), 37.6 (d, *J*_PC_ = 2.6 Hz), 31.7 (d, *J*_PC_ = 12 Hz), 31.74, 31.62, 29.0, 28.2, 27.9, 26.4* (d, ^1^*J*_PC_ = 89 Hz), 26.5 (d, ^1^*J*_PC_ = 89 Hz), 21.8* (d, *J*_PC_ = 6.3 Hz), 21.4 (d, *J*_PC_ = 5.7 Hz); ^31^P NMR (162 MHz, d_6_-DMSO) *δ* 45.7, 45.4; HRMS (*m/z*): [M + Na]^+^ calcd. for C_20_H_28_NNaO_6_P^+^, 432.1546 found, 432.1555.

*(2S)-2-({[(R)-1-(Benzyloxycarbonylamino)-3-(phenylthio)propyl](hydroxy)phosphoryl}methyl)-4-methylpentanoic acid* (**5p**): Phosphinic acid **1e** (2.0 g, 5.5 mmol) and acrylic acid **3c** (840 mg, 6.6 mmol) were subjected to the general HMDS-mediated P-C bond-forming reaction protocol. After the addition of HCl 2M and AcOEt, a solid was precipitated that was collected by filtration. The solid product was refluxed with AcOEt and the hot suspension was filtrated. The procedure was repeated to afford compound **5p** (d.r. 97:3) as a white solid in 59% yield (1.6 g, 2.5 mmol). m.p. 130–135 °C; [a]_D_^25^ = −14.2 (c = 1, DMSO); TLC *R*_f_ (CHCl_3_/MeOH/AcOH: 7/2/1) = 0.65. ^1^H NMR (500 MHz, d_6_-DMSO + 3%TFA) *δ* 7.65 (d, *J* = 9.5 Hz, 1H), 7.42–7.08 (m, 10H), 5.06 (s, 2H), 3.97–3.74 (m, 1H), 3.16–2.78 (m, 2H), 2.74–2.54 (m, 1H, overlapped by d_6_-DMSO), 2.01–1.74 (m, 3H), 1.72–1.23 (m, 4H), 0.83 [app t, *J* = 6.3 Hz, 6H]; ^13^C NMR (50 MHz, d_6_-DMSO) *δ* 176.4 (d, ^3^*J*_PC_ = 7.8 Hz), 156.4 (d, ^3^*J*_PC_ = 4.1 Hz), 137.2, 135.9, 129.2, 128.4, 128.1, 127.9, 127.6, 125.7, 65.6, 49.9 (d, ^1^*J*_PC_ = 104 Hz), 42.6 (d, *J*_PC_ = 8.9 Hz), 36.8 (d, *J*_PC_ = 4.0 Hz), 29.0 (d, *J*_PC_ = 12 Hz), 28.9 (d, ^1^*J*_PC_ = 89 Hz), 25.6, 23.0, 21.8; ^31^P NMR (81 MHz, d_6_-DMSO + 3%TFA) *δ* 46.0; HRMS (m/z): [M + H]^+^ calcd. for C_24_H_33_NO_6_PS^+^, 494.1761 found, 494.1760.

*3-{[(R)-1-(Benzyloxycarbonylamino)-4-(phenylthio)butyl](hydroxy)phosphoryl}-2-methylpropanoic acid* (**5q**): Phosphinic acid **1f** (937 mg, 2.47 mmol) and methacrylic acid (**3b**) (255 mg, 2.97 mmol) were subjected to the general HMDS-mediated P-C bond-forming reaction protocol. After the addition of HCl 2M and AcOEt, the two phases were separated and the organic layer was dried with Na_2_SO_4_ and evaporated in vacuo. The residue was solidified on standing, treated with Et_2_O and filtrated. Compound **5q** (d.r. 1:1) was isolated as a white solid in 92% yield (1.06 g, 2.27 mmol). TLC *R*_f_ (CHCl_3_/MeOH/AcOH: 7/2/1) = 0.61. ^1^H NMR (400 MHz, d_6_-DMSO) *δ* 7.50 & 7.49 (2 × d, *J* = 9.6 Hz, 1H), 7.26 & 7.21–7.13 (m, 10H), 5.07 (d, *J* = 13 Hz, 1H), 5.02 (dd, *J* = 13, 2 Hz, 1H), 3.70–3.57 (m, 1H), 3.03–2.85 (m, 2H), 2.73–2.58 (m, 1H), 2.09–1.97 (m, 1H), 1.94–1.81 (m, 1H), 1.76–1.48 (m, 4H), 1.17 & 1.16 (2 × d, *J* = 7.1 Hz, 3H), 5.07 (d, *J* = 13 Hz, 1H); ^13^C NMR (101 MHz, d_6_-DMSO) *δ* 176.7, 176.63, 176.56, 156.3 (d, ^3^*J*_PC_ = 4.5 Hz), 137.1, 136.3, 129.0, 128.3, 127.8, 127.7, 127.5, 127.4, 125.5, 65.5, 50.3 (d, ^3^*J*_PC_ = 104 Hz), 50.0 (d, ^3^*J*_PC_ = 105 Hz), 33.2, 33.1 (d, *J*_PC_ = 3.4 Hz), 31.4, 29.5 (d, ^1^*J*_PC_ = 89 Hz), 29.3 (d, ^1^*J*_PC_ = 89 Hz), 26.3, 25.5, 25.4, 18.8 (d, *J*_PC_ = 7.4 Hz), 18.5 (d, *J*_PC_ = 5.8 Hz). ^31^P NMR (162 MHz, d_6_-DMSO) *δ* 45.1; HRMS (*m/z*): [M + Na]^+^ calcd. for C_22_H_28_NNaO_6_P^+^, 456.1551 found, 456.1556.

*([1,1′:3′,1′′-Terphenyl]-5′-yl)-2-[({(R)-1-[(tert-butoxycarbonyl)amino]-3-phenylpropyl}(hydroxy)phosphoryl)methyl]propanoic acid* (**5r**): Phosphinic acid **1d** (100 mg, 0.33 mmol) and acrylic acid **3l** [20] (126 mg, 0.40 mmol) were subjected to the general HMDS-mediated P-C bond-forming reaction protocol. After the addition of HCl 2M and AcOEt, the two phases were separated and the organic layer was dried with Na_2_SO_4_ and evaporated in vacuo. The crude product was purified by silica gel column chromatography using CHCl_3_/MeOH/AcOH 7:0.01:0:01 → 7:0.4:0:4 as eluent solvent system. Compound **5r** (d.r. 2:1) was isolated as a white foam in 92% yield (193 mg, 2.27 mmol). TLC R_f_ (CHCl_3_/MeOH/AcOH: 7/0.5/0.5) = 0.43. ^1^H NMR (200 MHz, d_6_-DMSO) δ 7.83–7.06 (m, 18H), 3.73–3.47 (m, 1H), 3.20–2.92 (m, 3H), 2.80–2.58 (m, 1H), 2.58–2.37 (m, 1H, overlapped by d_6_-DMSO), 2.08–1.58 (m, 4H), 1.34 & 1.41 (2 × s, 9H); ^13^C NMR (50 MHz, d_6_-DMSO) δ 175.6, 175.5, 175.4, 155.9, 155.82, 155.76, 141.5, 141.4, 141.0, 140.4, 140.3, 140.2, 129.0, 128.8, 128.6, 128.4, 127.7, 127.1, 126.9, 125.9, 123.5, 78.4, 78.3, 49.5 (d, ^1^*J*_PC_ = 107 Hz), 49.1 (d, ^1^*J*_PC_ = 106 Hz), 31.9, 31.7, 29.2, 29.0, 28.3, 28.0, 26.5; ^31^P NMR (81 MHz, d_6_-DMSO) δ 47.0, 46.7; HRMS (m/z): [M + H]^+^ calcd. for C_36_H_41_NO_6_P^+^, 614.2666 found, 614.2666.

## 4. Conclusions

In this work, a useful method for the synthesis of phosphinic dipeptides of type **5** is presented. These compounds are valuable synthetic intermediates in the field of phosphinic peptides and protocols that facilitate their preparation are of great interest, especially when large quantities are required for medicinal chemistry purposes. Based on the observation that acrylic acids are neglected electrophile alternatives for the key *P*-Michael addition between α-aminophosphinic acids and acrylic acceptors, we propose that an unreactive mixture of an aminophosphinic and an acrylic acid can be activated under silylating conditions by acting simultaneously on both reactants, to afford in a single step the target compounds of type **5**. A great advantage of the presented method is that in several cases, bioactive (*R*,*S*)-isomers of type **5** can be directly isolated from the crude products by means of selective crystallization. This tendency is well-described in the literature and has been used in the past for the synthesis of several dipeptides of type **5**; for example, **5h** and **5m**, which are also described in this report and were found spectroscopically identical to previously reported compounds [20,23]. In particular, compounds **5h** [20] and **5m** [23] have been converted to tripeptides that were stereochemically characterized either by NMR [23] or by X-ray crystallographic analysis of their complexes with Zn-metalloproteases [38,39]. Taking together the availability of starting acids and the practicality of the proposed P-C bond-forming reaction, we believe that the reported protocol will greatly contribute to the simplification of synthetic procedures targeting this important class of compounds.

## Figures and Tables

**Table 1:**
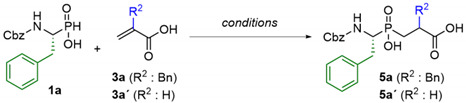
Optimization of reaction conditions.

Entry	Acrylic Acid (Equiv)	Reaction Conditions ^1^	Conversion of 1a, % (yield to 5a, %) ^2^
1	**3a** (1.3)	BSA (4 equiv), CH_2_Cl_2_, rt, 48 h	53 (41)
2	**3a′** (1.3)	BSA (4 equiv), CH_2_Cl_2_, rt, 48 h	90 (89) ^3^
3	**3a** (1.3)	TMSCl (7 equiv), *^i^* Pr_2_EtN (7 equiv), CH_2_Cl_2_, rt, 48 h	86 (81)
4	**3a** (1.2)	HMDS (6 equiv), 100 °C, 2 h	100 (99)
5	**3a** (1.2)	HMDS (6 equiv), 70 °C, 2 h	72 (70)

^1^ All reactions were performed in a 0.3 mmol scale of **1a** under inert conditions. ^2^ Conversions and yields were determined by integration of the ^31^P-NMR spectra of crude products. ^3^ Conversion to 5a′.

## Data Availability

Not applicable.

## Supplementary Materials

Synthetic protocol and characterization for compounds **3a** and **3c**–**k**. Copies of ^1^H, ^13^C and ^31^P NMR spectra for compounds **3a**, **3c**–**k** and **5a**–**r**.

## Sample Availability

Samples of the compounds are not available from the authors.

## Abbreviations

HMDS	1,1,1,3,3,3-hexamethyldisilazane
BSA	*N*,*O*-bis(trimethylsilyl)acetamide
TMSCl	chlorotrimethylsilane
