# Supplementary material for: Practical Synthesis of Phosphinic Dipeptides by Tandem Esterification of Aminophosphinic and Acrylic Acids under Silylating Conditions
Source: Molecules. 2022 Feb 12;27(4):1242. doi: 10.3390/molecules27041242 (PMC8876710; doi:10.3390/molecules27041242)
Supplement: Supplementary file 1 [file molecules-27-01242-s001.zip › molecules-1586221-supplementary.pdf]

PRACTICAL SYNTHESIS OF PHOSPHINIC DIPEPTIDES BY TANDEM ACTIVATION OF AMINOPHOSPHINIC AND ACRYLIC ACIDS UNDER SILYLATING CONDITIONS

Paraskevi Kokkala,[1] Kostas Voreakos,[1] Angelos Lelis,[1] Konstantinos Patiniotis,[1] Nikolaos Skoulikas,[1] Laurent Devel,[2] Angeliki Ziotopoulou,[1] Eleni Kaloumenou,[1] Dimitris Georgiadis*,[1].

[1]Department of Chemistry, Laboratory of Organic Chemistry, National and Kapodistrian University of Athens, Panepistimiopolis, Zografou, 15784, Athens, Greece. [2] Université Paris-Saclay, CEA, INRAE, Département Médicaments et Technologies pour la Santé (DMTS), SIMoS, 91191 Gif-sur-Yvette, France.

dgeorgia@chem.uoa.gr

SUPPORTING INFORMATION

TABLE OF CONTENTS

Experimental Section S-2

1. Synthesis and Characterization of Acrylic Acids of Type **3** S-2

2. Copies of NMR Spectra of Compounds of Type **3** and **5** S-9

Experimental Section

1. Synthesis and Characterization of Acrylic Acids of Type 3

General procedure for the synthesis of acrylic acids of type 3

Sodium (1.0 equiv) is slowly added and dissolved in abs. ethanol (1.4mL/mmol). Then, diethyl malonate (1.0 equiv) is added dropwise and the resulting mixture is stirred during 30 min at room temperature. Then, the respective bromide is added dropwise at room temperature and the resulting mixture is refluxed during 24 h. After removal of volatiles, H_2O is added and extractions with Et_2O (×3) are performed. The combined organic layers are dried with Na_2SO_4 and evaporated to afford the crude product which is assumed to contain only the target monoalkylated diethyl ester of type **6**. The residue is dissolved in EtOH (0.5 mL/mmol of **6**) and cooled at 0°C. At the same temperature, a solution of KOH (2.0 equiv) in EtOH (1.5 mL/mmol of **6**) is slowly added. The mixture is stirred at rt and the progress of the reaction is followed by TLC, until diethylester **6** is fully consumed (if needed, up to 1.0 equiv of KOH is gradually added). Typically, reaction time is 24h. The mixture is concentrated and the residue is diluted H_2O and Et_2O. The aqueous phase is separated, washed with Et_2O (×2), acidified with HCl 2M to pH ~1 and extracted with AcOEt (×3). The combined organic layers are dried over Na_2SO_4 and evaporated to afford diacid of type **7**. The latter is dissolved/suspended to AcOEt (1.8 mL/mmol of **7**) and Et_2NH (1.2 equiv) is added slowly at 0 °C. The temperature is raised at rt, HCHO (1.4 equiv) is added and the mixture is refluxed for 4 h. Then, the mixture is concentrated, treated with aq. $NaHCO_3$ 5% and washed with Et_2O (×3). The aqueous phase is acidified with HCl 2M to pH ~1 and extracted with AcOEt (×3). The combined

organic layers are dried over Na_2SO_4 and evaporated to afford acrylic acid of type **3** in adequately pure form to be used in the *P*-Michael reaction without further purification.

2-Benzylacrylic acid (3a)

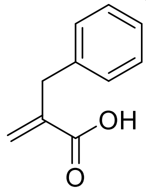

3a

Compound **3a** was prepared according to the general procedure for the synthesis of acrylic acids in a 18.7 mmol scale of diethyl malonate. The final product **3a** was obtained as a white solid in 40% (1.21 g) yield for 3 steps. M.p. 64–68 °C; ^{1}H NMR (200 MHz, $CDCl_3$) δ 7.65 – 7.30 (m. 5H), 6.66 (s, 1H), 5.78 (s, 1H), 3.89 (s, 2H); ^{13}C NMR (50 MHz, $CDCl_3$) δ 172.9, 139.6, 138.4, 129.1, 128.8, 128.5, 126.5, 37.5; HRMS (m/z): [M - H]$^-$ calcd. for $C_{10}H_9O_2^-$, 161.0608 found, 161.0603.

4-Methyl-2-methylenepentanoic acid (3c)

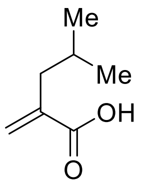

3c

Compound **3c** was prepared according to the general procedure for the synthesis of acrylic acids in a 13.9 mmol scale of diethyl malonate. The final product **3c** was obtained as a light yellow oil in 60% (1.06 g) yield for 3 steps. ^{1}H NMR (200 MHz, $CDCl_3$) δ 6.32 (d, J = 1.2 Hz, 1H), 5.61 (d, J = 1.0 Hz, 1H), 2.17 (d, J = 7.1 Hz, 2H), 1.82 (m, 1H), 0.89

(d, J = 6.6 Hz, 6H); ^{13}C NMR (50 MHz, $CDCl_3$) δ 173.2, 139.2, 128.4, 41.0, 27.2, 22.4; HRMS (m/z): $[M - H]^-$ calcd. for $C_7H_{11}O_2^-$, 127.0765 found, 127.0766.

2-Methylene-4-phenylbutanoic acid (3d)

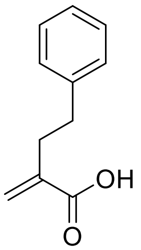

3d

Compound **3d** was prepared according to the general procedure for the synthesis of acrylic acids in a 4.2 mmol scale of diethyl malonate. The final product **3d** was obtained as a white solid in 47% (0.35 g) yield for 3 steps. ^{1}H NMR (200 MHz, $CDCl_3$) δ 7.40 – 7.13 (m. 5H), 6.37 (s, 1H), 5.65 (s, 1H), 2.92 – 2.79 (m, 2H), 2.74 – 2.60 (m, 2H); ^{13}C NMR (50 MHz, $CDCl_3$) δ 172.9, 141.4, 139.3, 128.6, 128.6, 128.5, 128.2, 126.2, 34.9, 33.7; HRMS (m/z): $[M - H]^-$ calcd. for $C_{11}H_{11}O_2^-$, 175.0765 found, 175.0765.

2-Methylene-5-phenylpentanoic acid (3e)

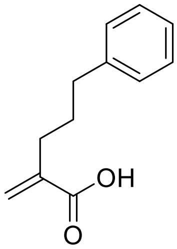

3e

Compound **3e** was prepared according to the general procedure for the synthesis of acrylic acids in a 7.5 mmol scale of diethyl malonate. The final product **3e** was obtained

as a white solid in 44% (0.63 g) yield for 3 steps. ^{1}H NMR (200 MHz, $CDCl_3$) δ 7.51 – 7.22 (m. 5H), 6.48 (d, J = 0.8 Hz, 1H), 5.78 (d, J = 1.2 Hz, 1H), 2.29 (t, J = 7.7 Hz, 2H), 2.50 (t, J = 7.7 Hz, 2H), 2.07 – 1.88 (m, 2H); ^{13}C NMR (50 MHz, $CDCl_3$) δ 173.2, 142.0, 140.0, 128.5, 128.4, 127.4, 125.9, 35.4, 31.1, 30.0; HRMS (m/z): [M - H]$^-$ calcd. for $C_{12}H_{13}O_2^-$, 189.0921 found, 189.0916.

2-Methylenepent-4-enoic acid (3f)

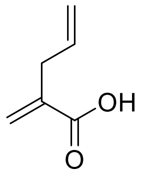

3f

Compound **3f** was prepared according to the general procedure for the synthesis of acrylic acids in a 31 mmol scale of diethyl malonate. The final product **3f** was obtained as a light yellow oil in 24% (0.85 g) yield for 3 steps. ^{1}H NMR (200 MHz, $CDCl_3$) δ 6.36 (q, J = 1.0 Hz, 1H), 5.98 – 5.75 (m, 1H), 5.70 (dd, J = 2.5, 1.1 Hz, 1H), 5.17 – 5.11 (m, 1H), 5.09 – 5.04 (m, 1H), 3.06 (d, J = 6.6 Hz, 2H); ^{13}C NMR (50 MHz, $CDCl_3$) δ 172.9, 138.5, 134.9, 128.2, 117.2, 35.5; HRMS (m/z): [M - H]$^-$ calcd. for $C_6H_7O_2^-$, 111.0452 found, 111.0449.

2-Methylenepent-4-ynoic acid (3g)

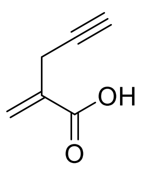

3g

Compound **3g** was prepared according to the general procedure for the synthesis of acrylic acids in a 23 mmol scale of diethyl malonate. The final product **3g** was obtained as a light yellow thick oil in 74% (1.84 g) yield for 3 steps. ^{1}H NMR (200 MHz, $CDCl_3$) δ 6.50 (d, J = 0.9 Hz, 1H), 6.19 (d, J = 0.9 Hz, 1H), 3.20 - 3.29 (m, 2H), 2.64 (t, J = 2.6 Hz, 1H); ^{13}C NMR (50 MHz, $CDCl_3$) δ 171.8, 134.5, 129.2, 79.9, 72.4, 21.3; HRMS (m/z): $[M - H]^-$ calcd. for $C_6H_5O_2^-$, 109.0295 found, 109.0292.

2-([1,1'-Biphenyl]-4-ylmethyl)acrylic acid (3h)

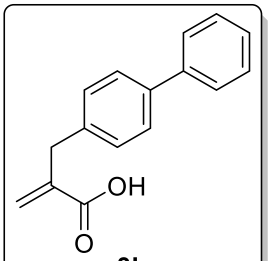

3h

Compound **3h** was prepared according to the general procedure for the synthesis of acrylic acids in a 4.4 mmol scale of diethyl malonate. The final product **3h** was obtained as a white solid in 63% (0.66 g) yield for 3 steps. ^{1}H NMR (200 MHz, $CDCl_3$) δ 7.57 – 7.02 (m, 11H), 6.34 (s, 1H), 5.57 (s, 1H), 3.59 (s, 2H); ^{13}C NMR (50 MHz, $CDCl_3$) δ 172.5, 141.0, 139.5, 137.7, 129.6, 129.0, 128.9, 127.4, 127.3, 127.2, 37.4; HRMS (m/z): $[M - H]^-$ calcd. for $C_{16}H_{14}O_2^-$, 237.0921 found, 237.0923.

4-(1,3-Dioxolan-2-yl)-2-methylenebutanoic acid (3i)

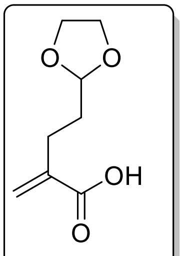

3i

Compound **3i** was prepared according to the general procedure for the synthesis of acrylic acids in a 5 mmol scale of diethyl malonate. The final product **3i** was obtained as a white solid in 57% (0.49 g) yield for 3 steps. ^{1}H NMR (200 MHz, $CDCl_3$) δ 6.30 (s, 1H), 5.70 (q, J = 1.3 Hz, 1H), 4.91 (t, J = 4.6 Hz, 1H), 4.03 – 3.81 (m, 4H), 2.51 – 2.36 (m, 2H), 1.94 – 1.77 (m, 2H); ^{13}C NMR (50 MHz, $CDCl_3$) δ 172.4, 139.5, 127.5, 103.9, 65.1, 32.5, 26.1; HRMS (m/z): [M - H]$^-$ calcd. for $C_8H_{11}O_2^-$, 171.0663 found, 171.0659.

5-[(*tert*-Butoxycarbonyl)amino]-2-methylenepentanoic acid (3j)

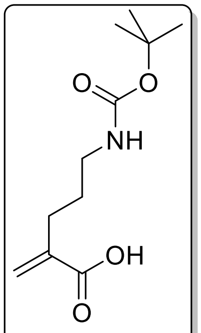

3j

Compound **3j** was prepared according to the general procedure for the synthesis of acrylic acids in a 7.5 mmol scale of diethyl malonate. The final product **3j** was obtained as a white solid in 47% (0.80 g) yield for 3 steps. ^{1}H NMR (200 MHz, $CDCl_3$) δ 6.27 (s, 1H), 5.64 (s, 1H), 3.11 (t, J = 6.8 Hz, 2H), 2.31 (t, J = 7.8 Hz, 2H), 1.66 (quint, J = 7.1 Hz, 2H), 1.42 (s, 9H); ^{13}C NMR (50 MHz, $CDCl_3$) δ 172.0, 156.3, 139.7, 127.4, 79.5,

41.2, 40.2, 29.0, 28.6; HRMS (m/z): $[M - H]^-$ calcd. for $C_{11}H_{18}NO_4^-$, 228.1241 found, 228.1237.

4-(*tert*-Butoxy)-2-methylene-4-oxobutanoic acid (3k)

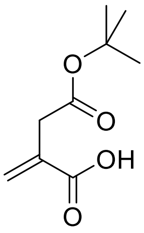

3k

Compound **3k** was prepared according to the general procedure for the synthesis of acrylic acids in a 15 mmol scale of diethyl malonate. NaH in THF was used for the alkylation of diethylmalonate [1]. The final product **3k** was obtained as a white solid in 84% (2.34 g) yield for 3 steps. ^{1}H NMR (200 MHz, $CDCl_3$) δ 6.42 (d, J = 1.1 Hz, 1H), 5.78 (q, J = 1.1 Hz, 1H), 3.25 (d, J = 0.7 Hz, 2H), 1.44 (s, 9H); ^{13}C NMR (50 MHz, $CDCl_3$) δ 171.7, 170.0, 134.0, 130.0, 81.3, 38.6, 27.9.; HRMS (m/z): $[M + H]^+$ calcd. for $C_9H_{15}O_4^+$, 209.0784 found, 209.0787.

Copies of NMR spectra of compounds of type **3** and **5**

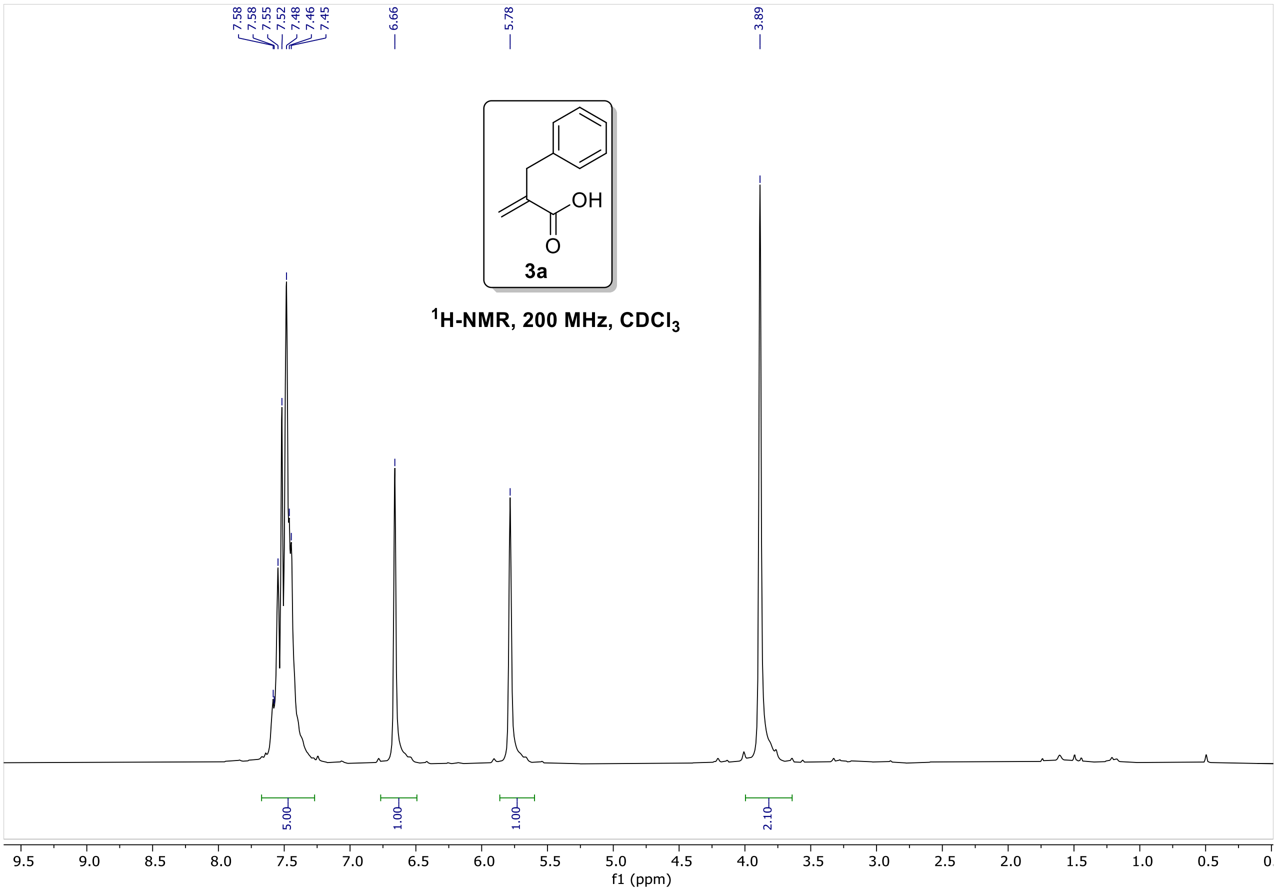
7.58
7.58
7.55
7.52
7.48
7.46
7.45
6.66
5.78
3.89
OH
O
3a
1H-NMR, 200 MHz, CDCl3
5.00
1.00
1.00
2.10
9.5
9.0
8.5
8.0
7.5
7.0
6.5
6.0
5.5
5.0
4.5
4.0
3.5
3.0
2.5
2.0
1.5
1.0
0.5
f1 (ppm)

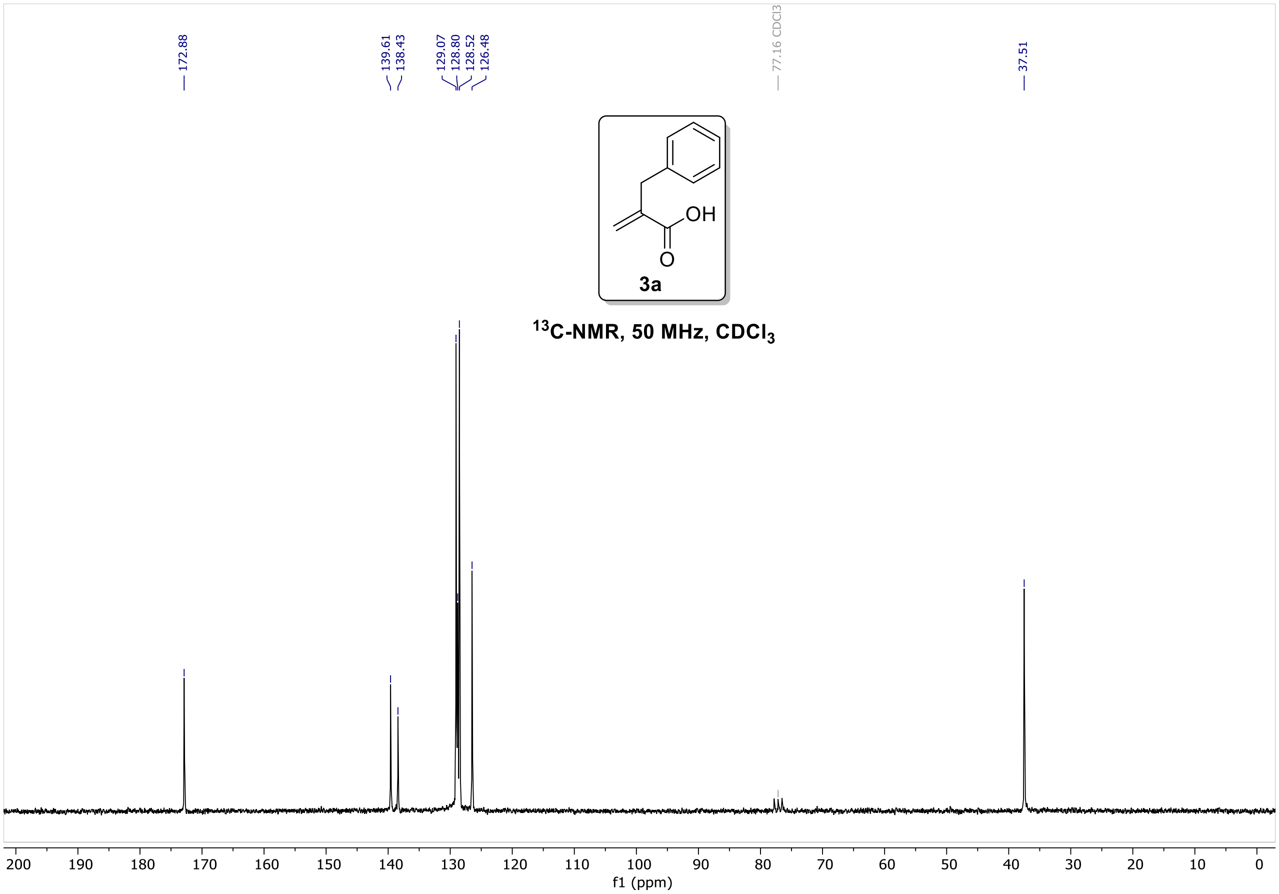
172.88
139.61
138.43
129.07
128.80
128.52
126.48
77.16 CDCl3
37.51
OH
O
3a
13C-NMR, 50 MHz, CDCl3
200
190
180
170
160
150
140
130
120
110
100
90
80
70
60
50
40
30
20
10
0
f1 (ppm)

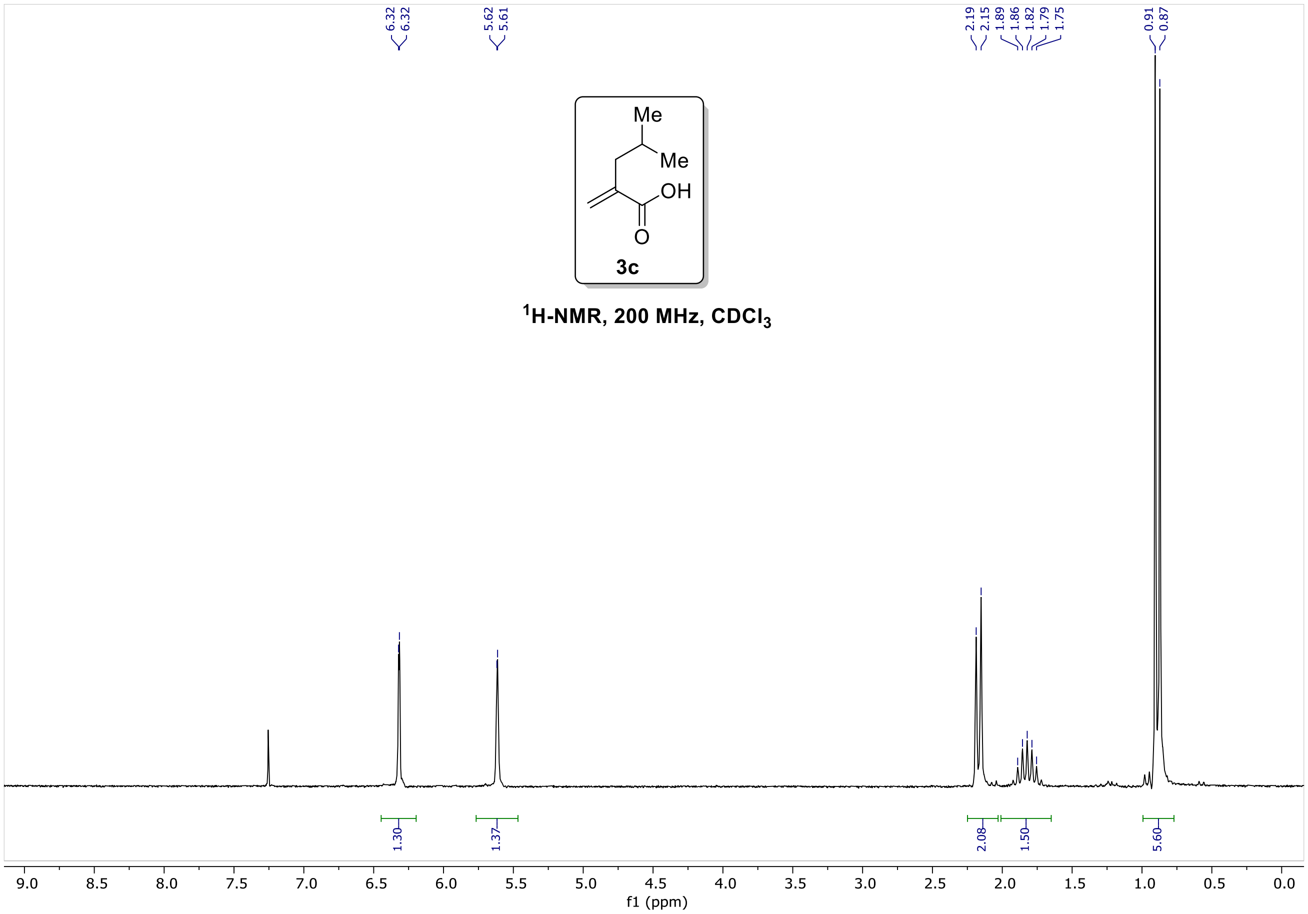
6.32
6.32
5.62
5.61
2.19
2.15
1.89
1.86
1.82
1.79
1.75
0.91
0.87
Me
Me
OH
O
3c
^{1}H-NMR, 200 MHz, $CDCl_3$
1.30
1.37
2.08
1.50
5.60
9.0 8.5 8.0 7.5 7.0 6.5 6.0 5.5 5.0 4.5 4.0 3.5 3.0 2.5 2.0 1.5 1.0 0.5 0.0
f1 (ppm)

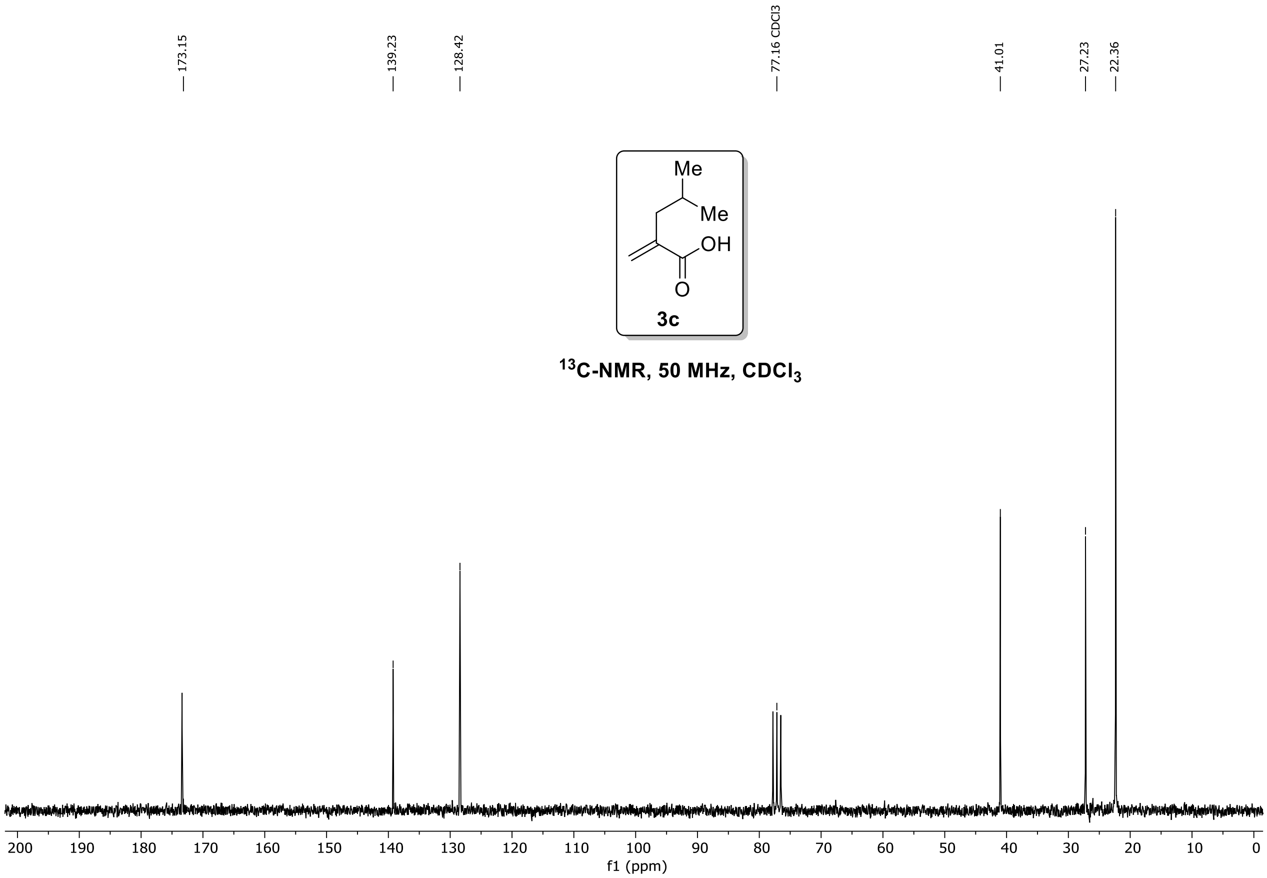
173.15
139.23
128.42
77.16 CDCl3
41.01
27.23
22.36
Me
Me
OH
O
3c
^{13}C-NMR, 50 MHz, $CDCl_3$
200 190 180 170 160 150 140 130 120 110 100 90 80 70 60 50 40 30 20 10 0
f1 (ppm)

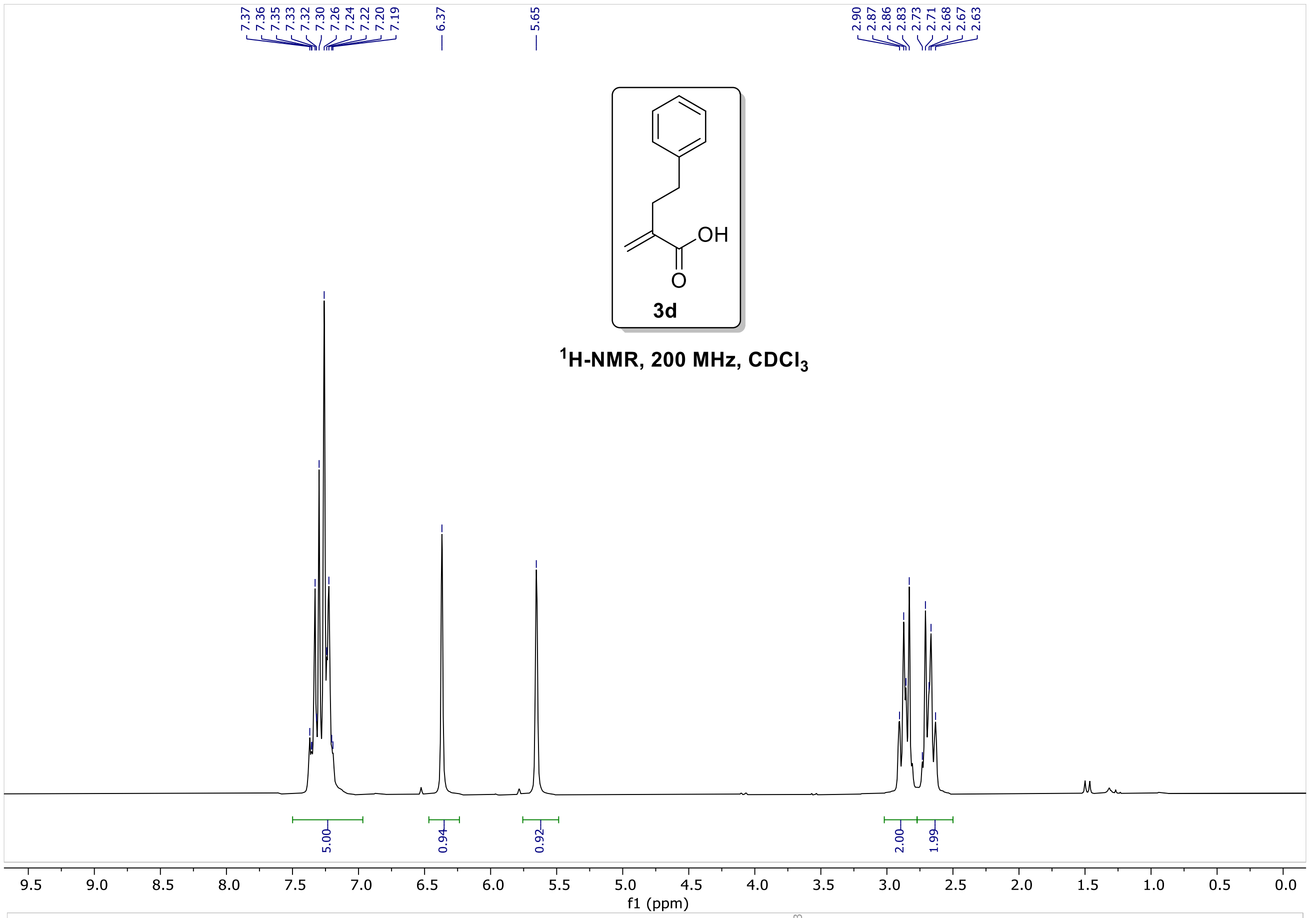
7.37
7.36
7.35
7.33
7.32
7.30
7.26
7.24
7.22
7.20
7.19
6.37
5.65
2.90
2.87
2.86
2.83
2.73
2.71
2.68
2.67
2.63
OH
O
3d
1H-NMR, 200 MHz, CDCl3
5.00
0.94
0.92
2.00
1.99
f1 (ppm)

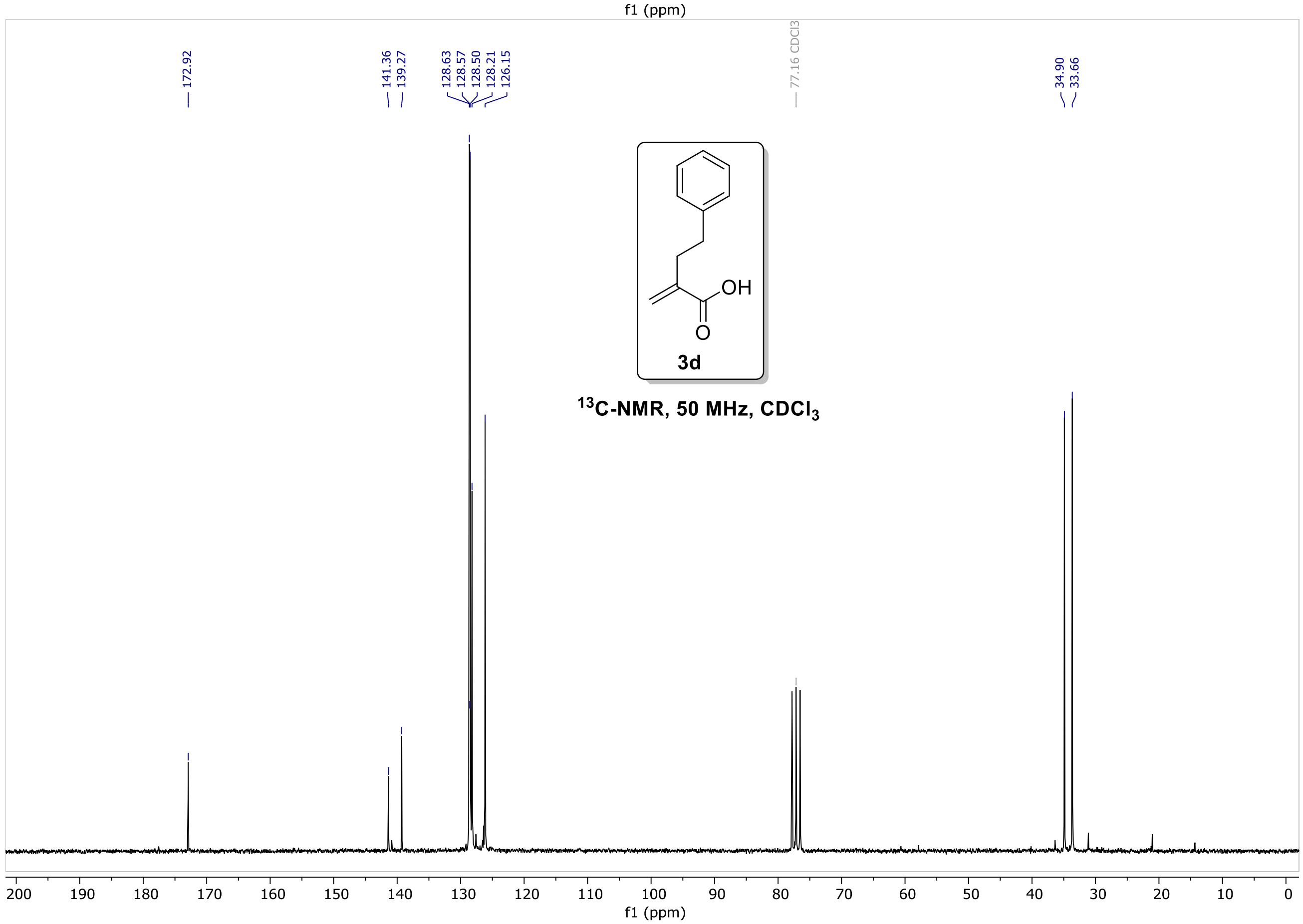
172.92
141.36
139.27
128.63
128.57
128.50
128.21
126.15
77.16 CDCl3
34.90
33.66
OH
O
3d
13C-NMR, 50 MHz, CDCl3
f1 (ppm)

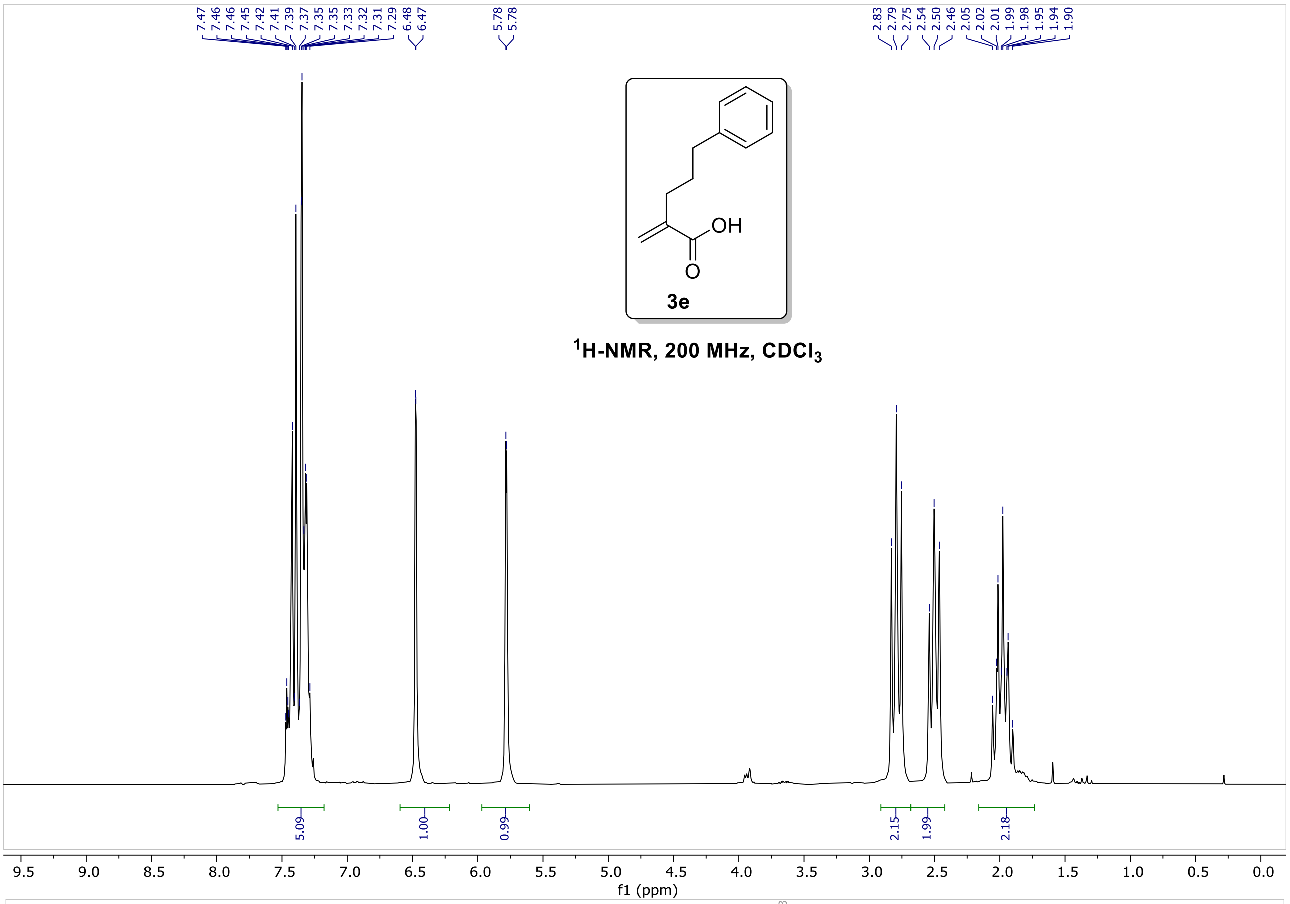

^{1}H-NMR, 200 MHz, $CDCl_3$

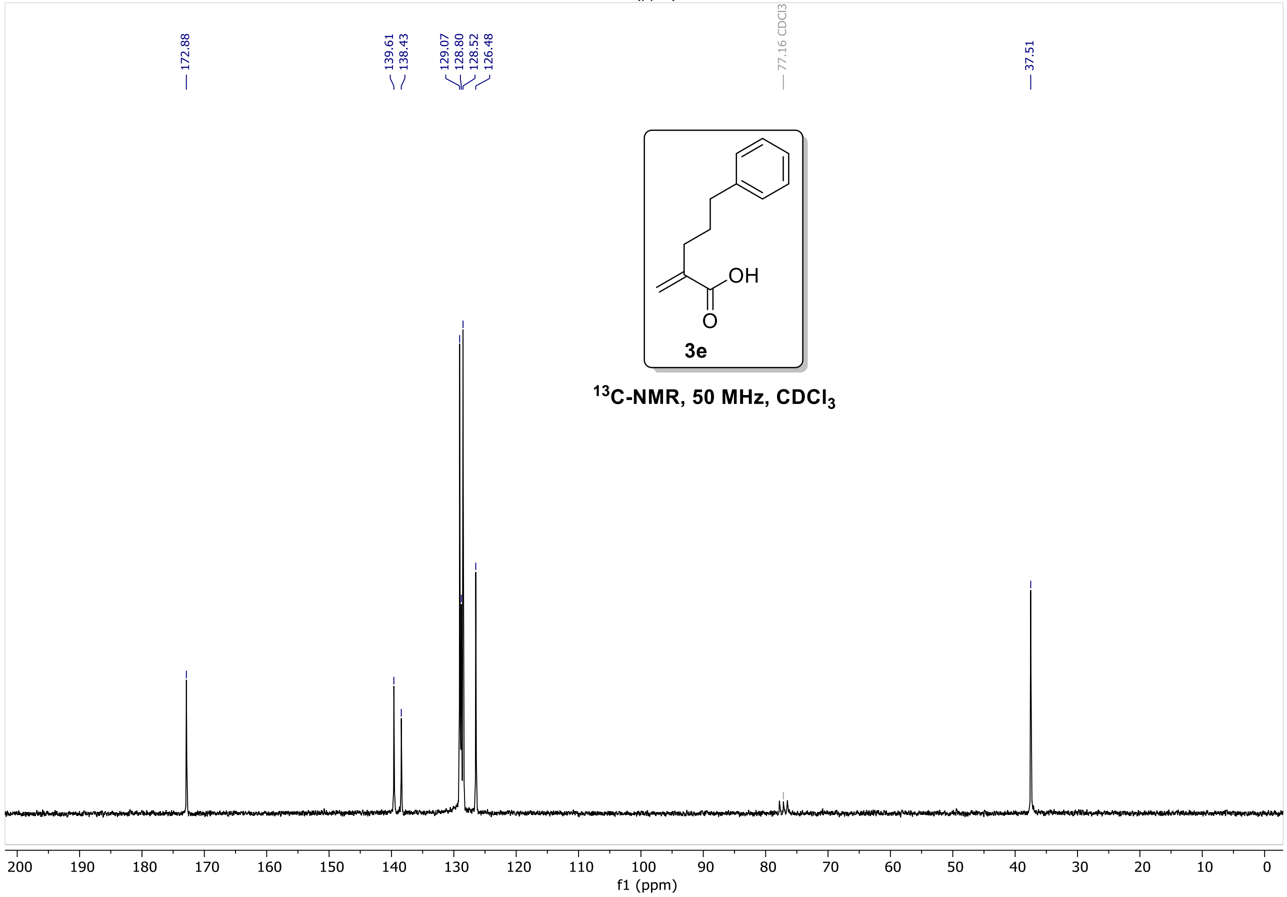

^{13}C-NMR, 50 MHz, $CDCl_3$

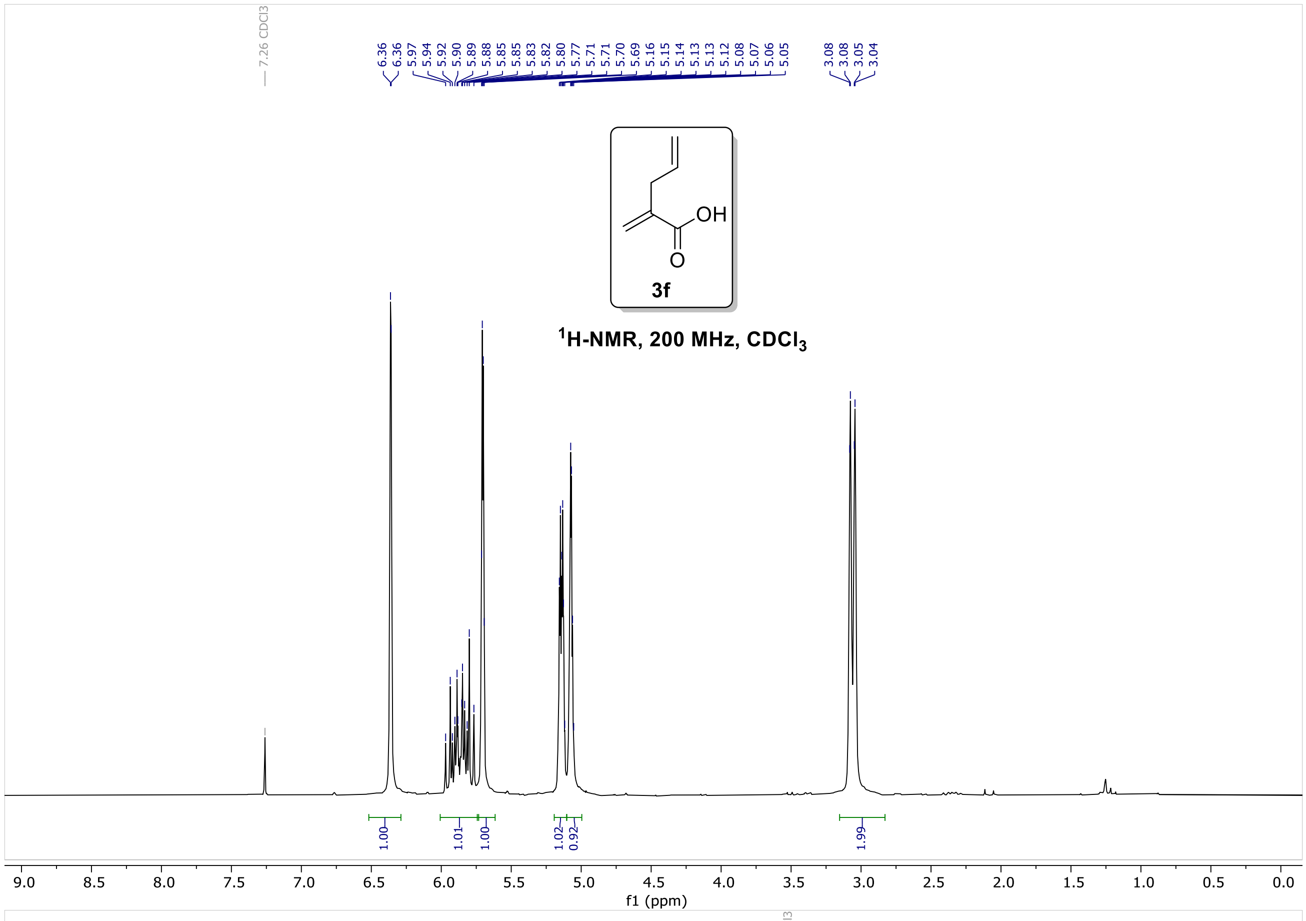
7.26 CDCl3
6.36
6.36
5.97
5.94
5.92
5.90
5.89
5.88
5.85
5.85
5.83
5.82
5.80
5.77
5.71
5.71
5.70
5.69
5.16
5.15
5.14
5.13
5.13
5.12
5.08
5.07
5.06
5.05
3.08
3.08
3.05
3.04
OH
O
3f
^{1}H-NMR, 200 MHz, CDCl$_3$
1.00
1.01
1.00
1.02
0.92
1.99
9.0
8.5
8.0
7.5
7.0
6.5
6.0
5.5
5.0
4.5
4.0
3.5
3.0
2.5
2.0
1.5
1.0
0.5
0.0
f1 (ppm)

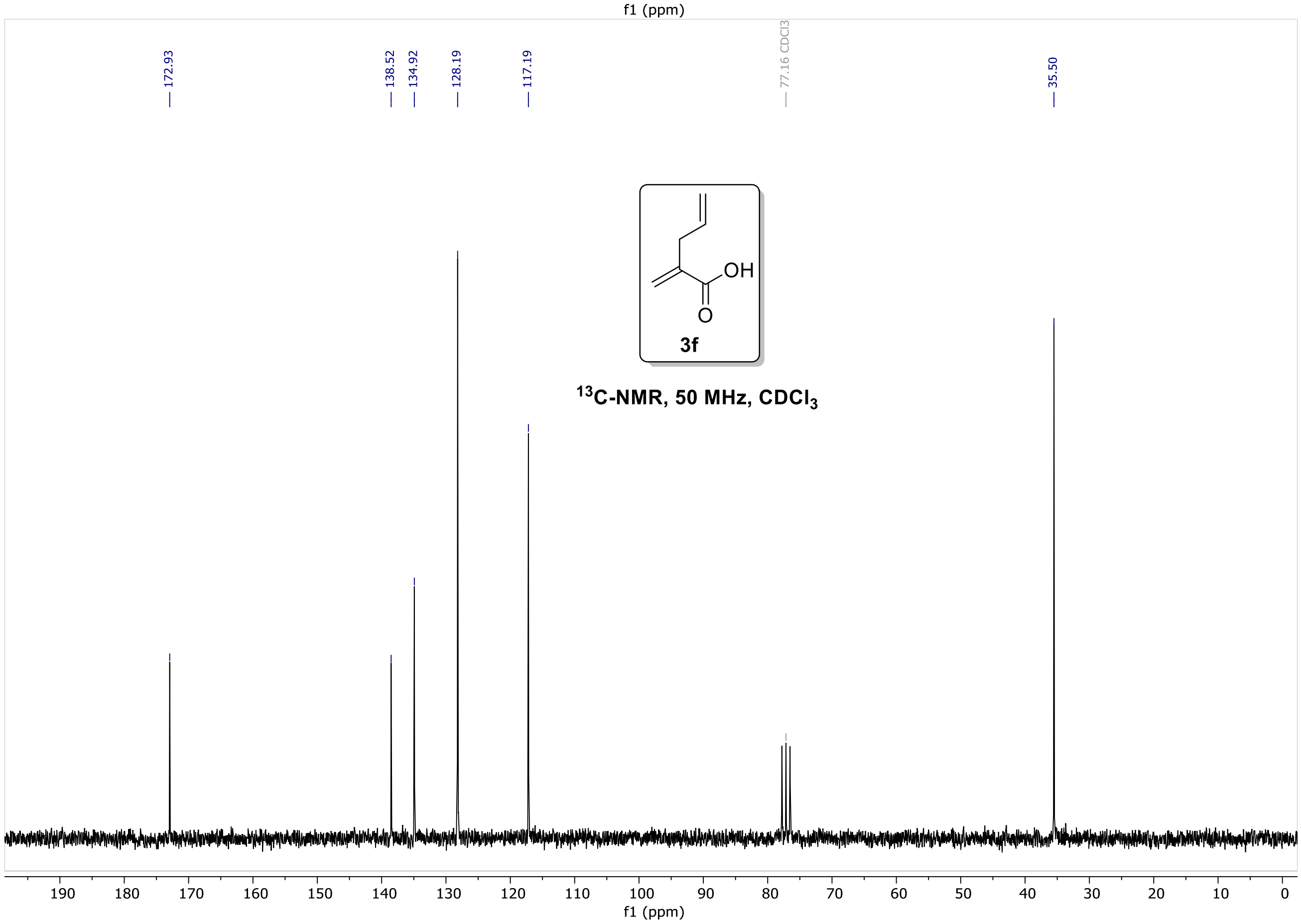
172.93
138.52
134.92
128.19
117.19
77.16 CDCl3
35.50
OH
O
3f
^{13}C-NMR, 50 MHz, CDCl$_3$
190
180
170
160
150
140
130
120
110
100
90
80
70
60
50
40
30
20
10
0
f1 (ppm)

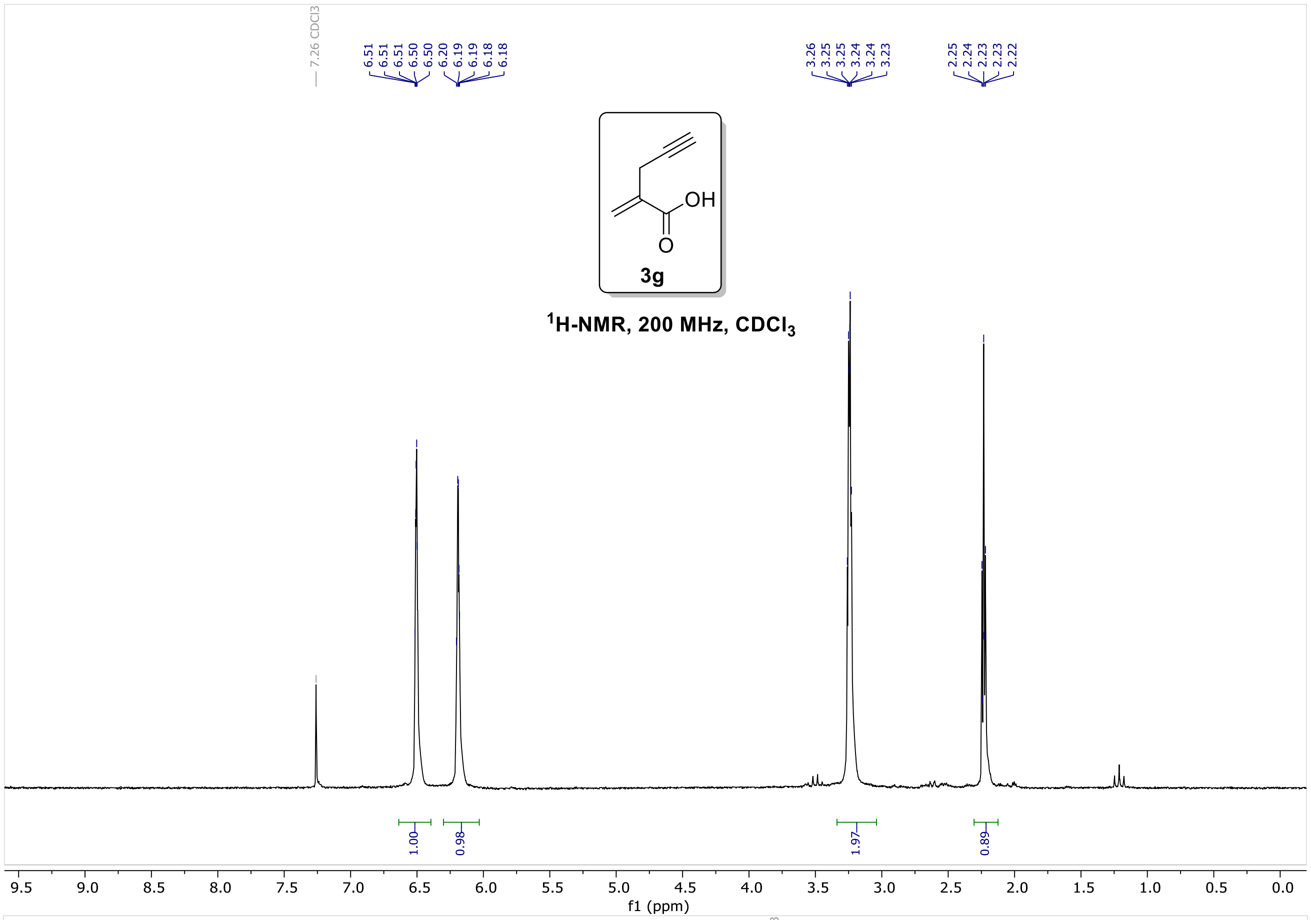
7.26 CDCl3
6.51
6.51
6.51
6.50
6.50
6.20
6.19
6.19
6.18
6.18
3.26
3.25
3.25
3.24
3.24
3.23
2.25
2.24
2.23
2.23
2.22
OH
O
3g
1H-NMR, 200 MHz, CDCl3
1.00
0.98
1.97
0.89
f1 (ppm)

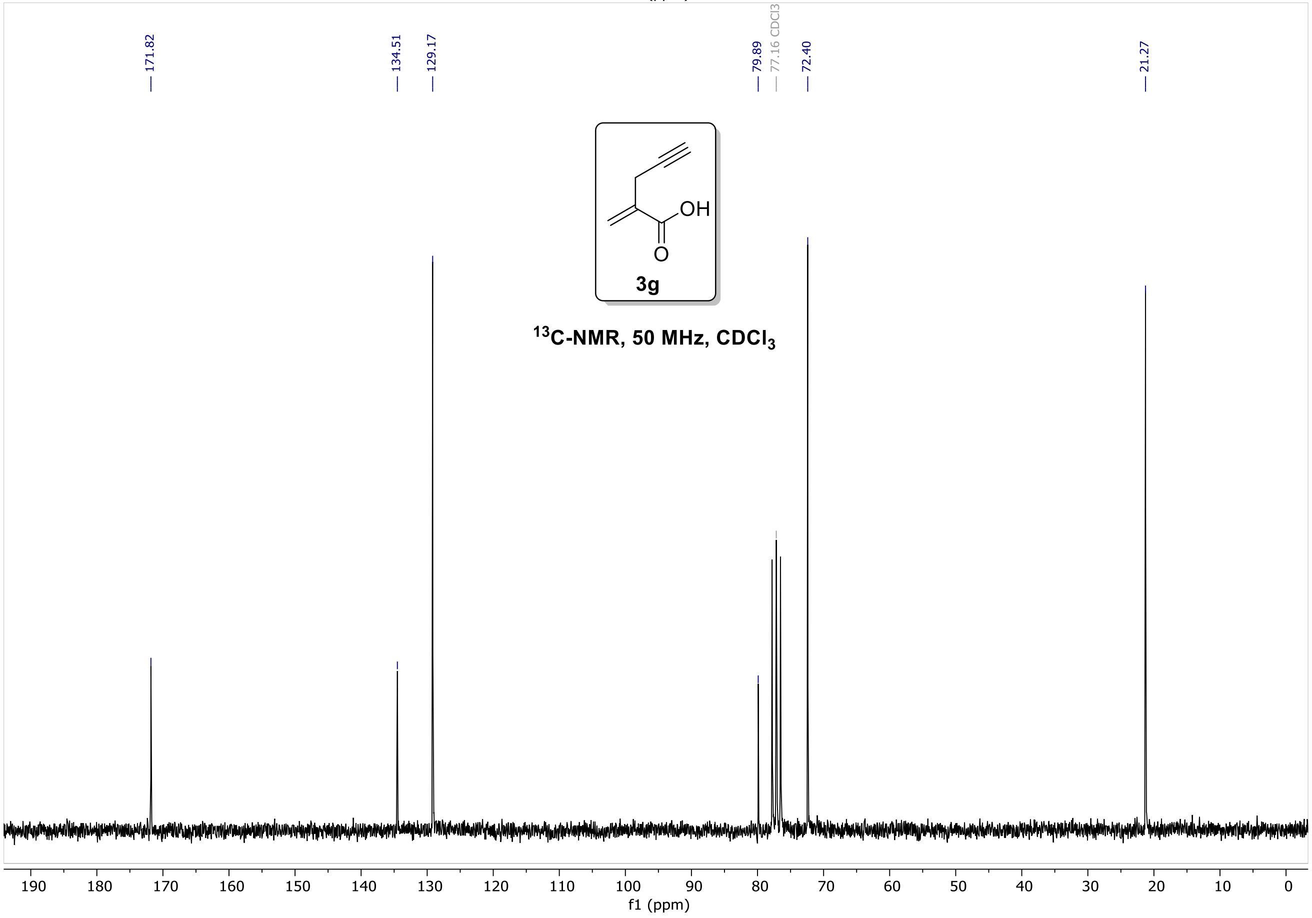
171.82
134.51
129.17
79.89
77.16 CDCl3
72.40
21.27
OH
O
3g
13C-NMR, 50 MHz, CDCl3
f1 (ppm)

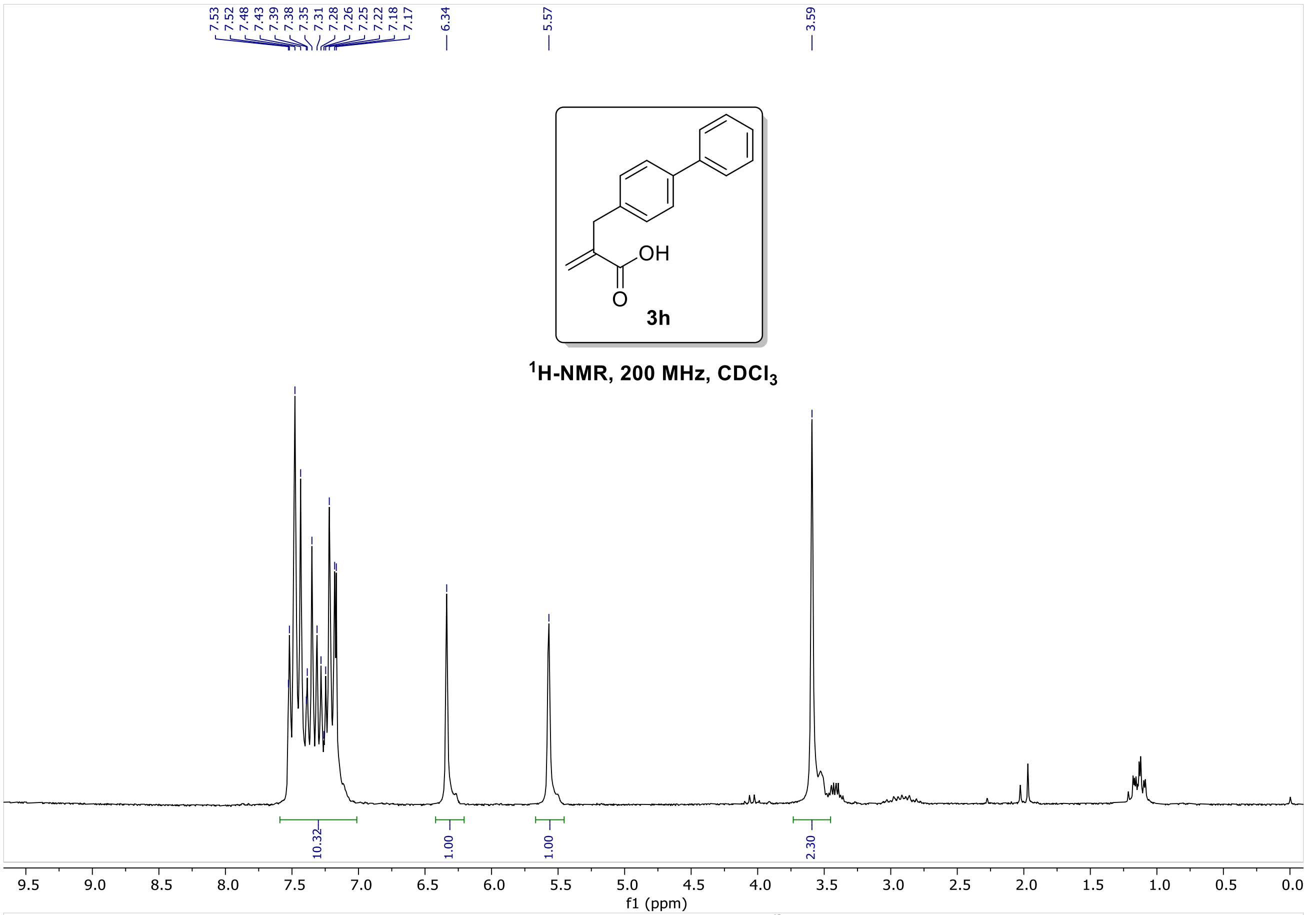
7.53
7.52
7.48
7.43
7.39
7.38
7.35
7.31
7.28
7.26
7.25
7.22
7.18
7.17
6.34
5.57
3.59
OH
O
3h
^{1}H-NMR, 200 MHz, CDCl$_3$
10.32
1.00
1.00
2.30
9.5 9.0 8.5 8.0 7.5 7.0 6.5 6.0 5.5 5.0 4.5 4.0 3.5 3.0 2.5 2.0 1.5 1.0 0.5 0.0
f1 (ppm)

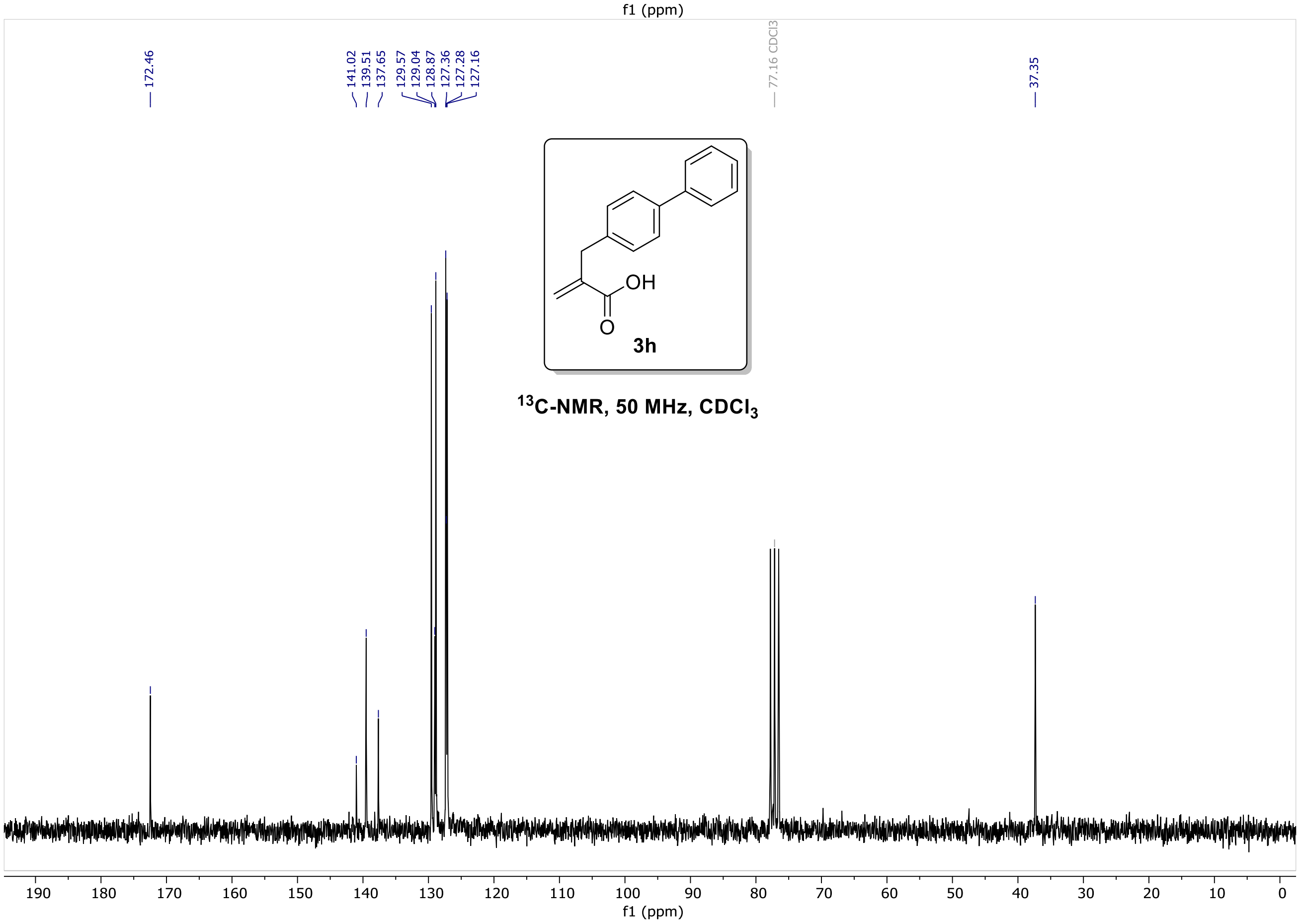
172.46
141.02
139.51
137.65
129.57
129.04
128.87
127.36
127.28
127.16
77.16 CDCl3
37.35
OH
O
3h
^{13}C-NMR, 50 MHz, CDCl$_3$
190 180 170 160 150 140 130 120 110 100 90 80 70 60 50 40 30 20 10 0
f1 (ppm)

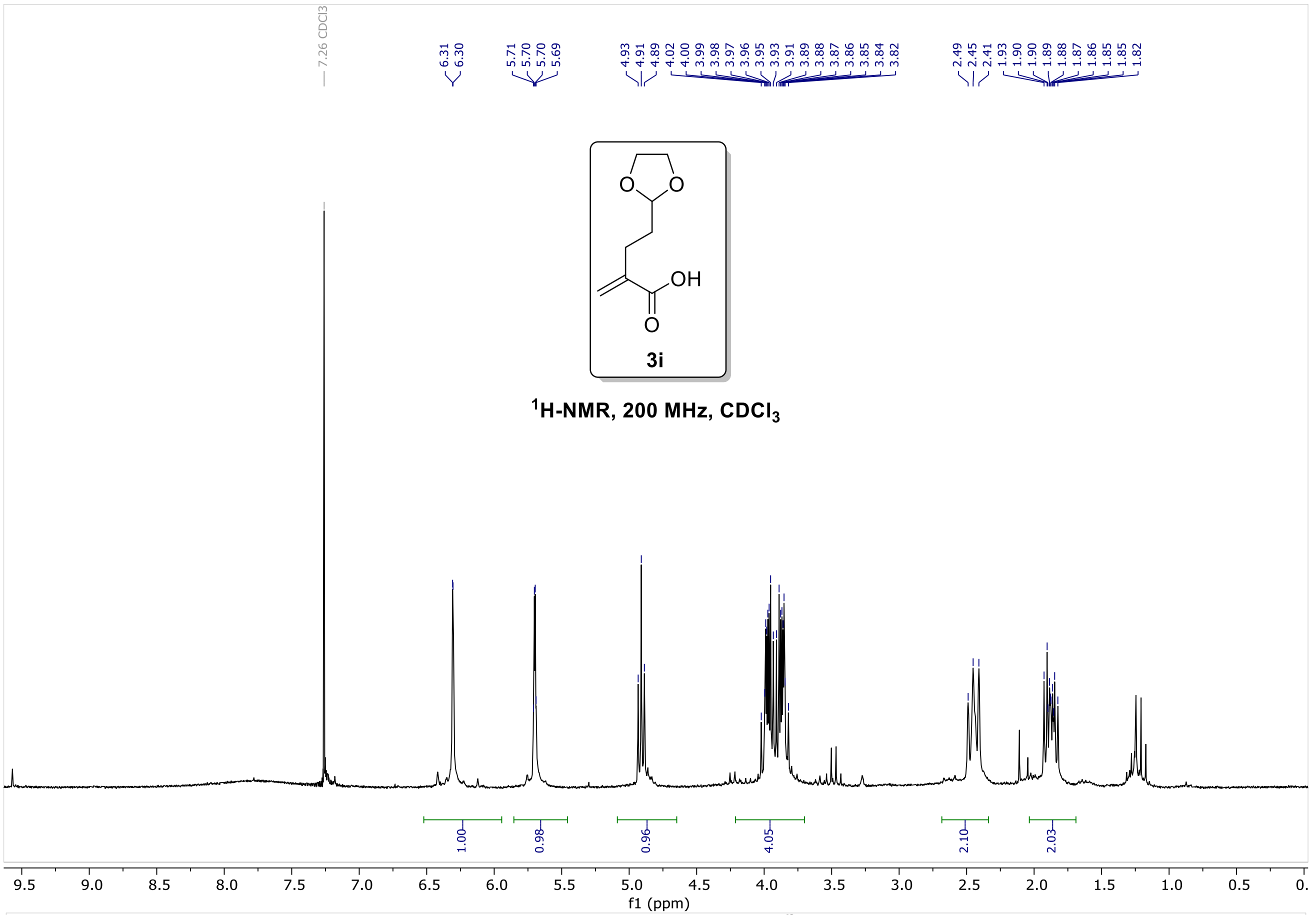
7.26 CDCl3
6.31
6.30
5.71
5.70
5.70
5.69
4.93
4.91
4.89
4.02
4.00
3.99
3.98
3.97
3.96
3.95
3.93
3.91
3.89
3.88
3.87
3.86
3.85
3.84
3.82
2.49
2.45
2.41
1.93
1.90
1.90
1.89
1.88
1.87
1.86
1.85
1.85
1.82
O
O
OH
O
3i
1H-NMR, 200 MHz, CDCl3
1.00
0.98
0.96
4.05
2.10
2.03
f1 (ppm)

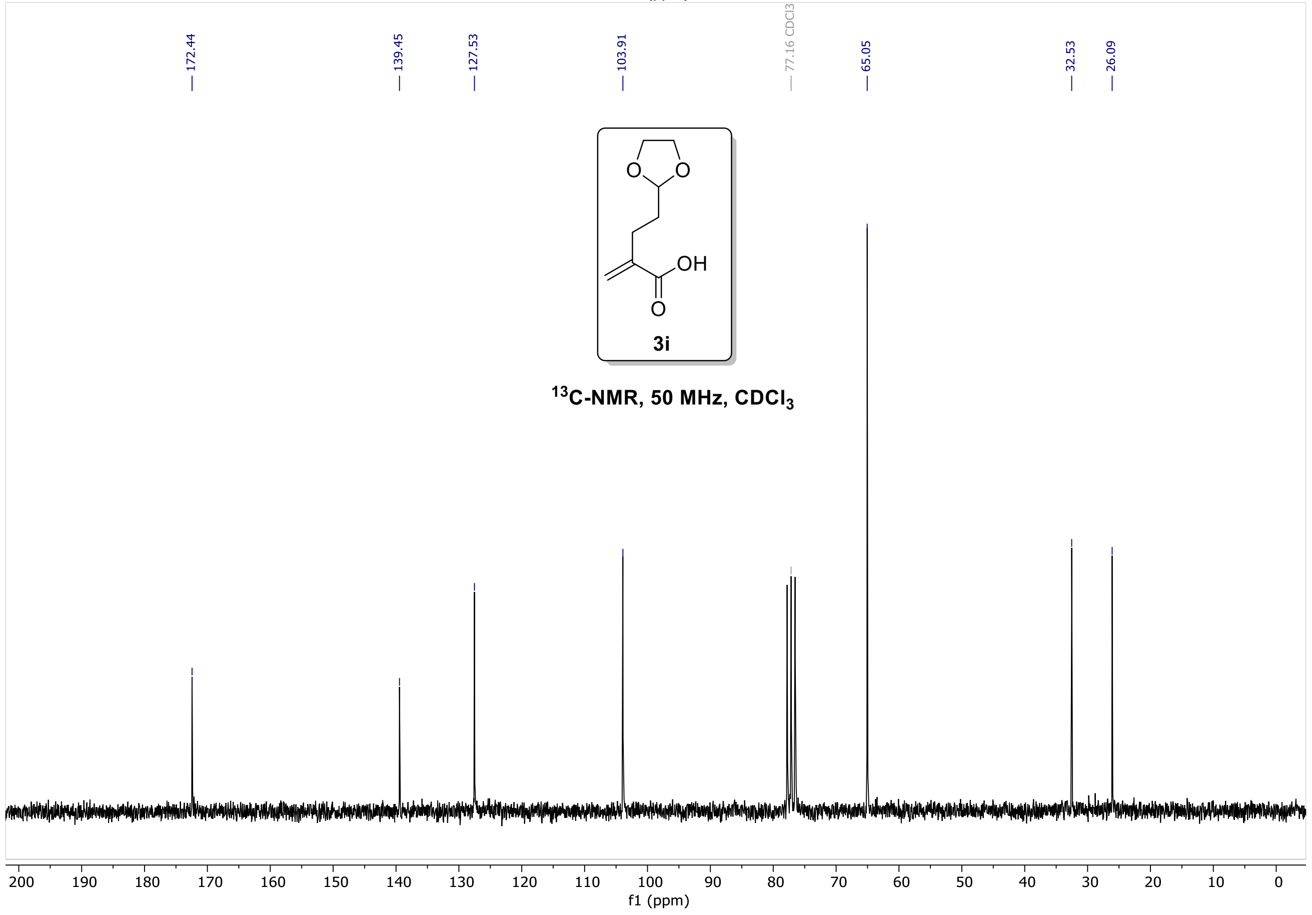
172.44
139.45
127.53
103.91
77.16 CDCl3
65.05
32.53
26.09
O
O
OH
O
3i
13C-NMR, 50 MHz, CDCl3
f1 (ppm)

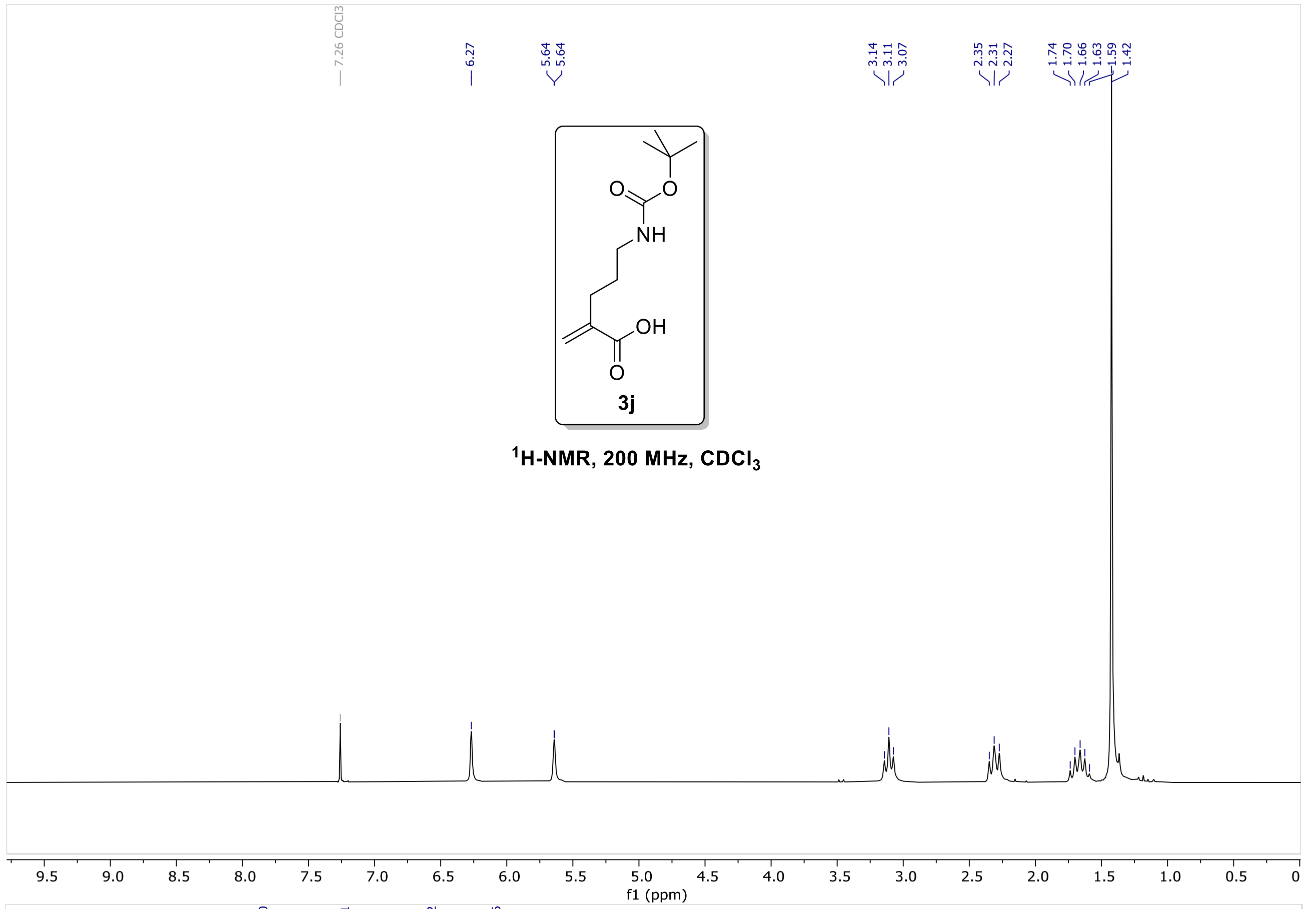
7.26 CDCl3
6.27
5.64
5.64
3.14
3.11
3.07
2.35
2.31
2.27
1.74
1.70
1.66
1.63
1.59
1.42
3j
^{1}H-NMR, 200 MHz, CDCl$_3$
f1 (ppm)

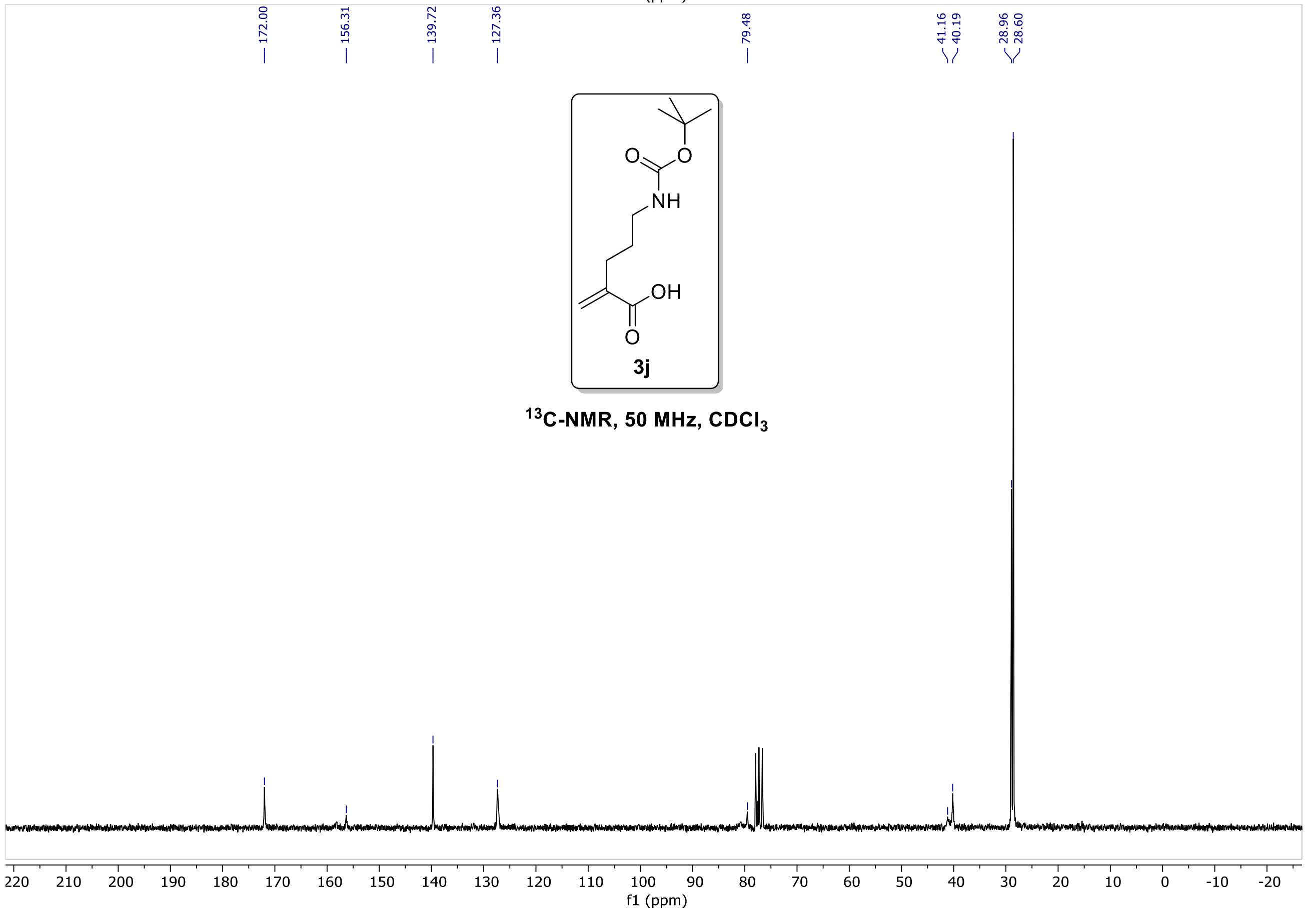
172.00
156.31
139.72
127.36
79.48
41.16
40.19
28.96
28.60
3j
^{13}C-NMR, 50 MHz, CDCl$_3$
f1 (ppm)

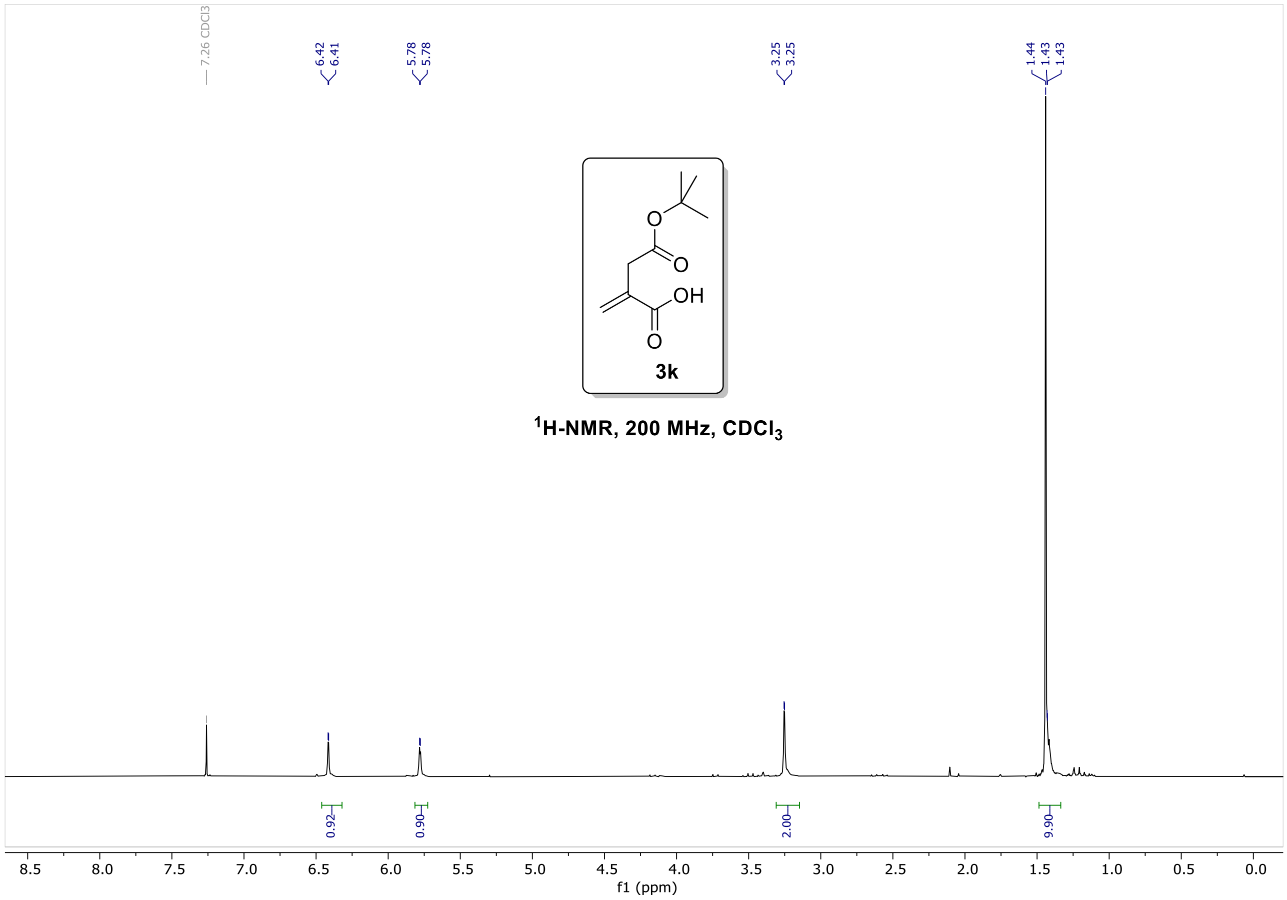
7.26 CDCl3
6.42
6.41
5.78
5.78
3.25
3.25
1.44
1.43
1.43
OH
3k
1H-NMR, 200 MHz, CDCl3
0.92
0.90
2.00
9.90
f1 (ppm)

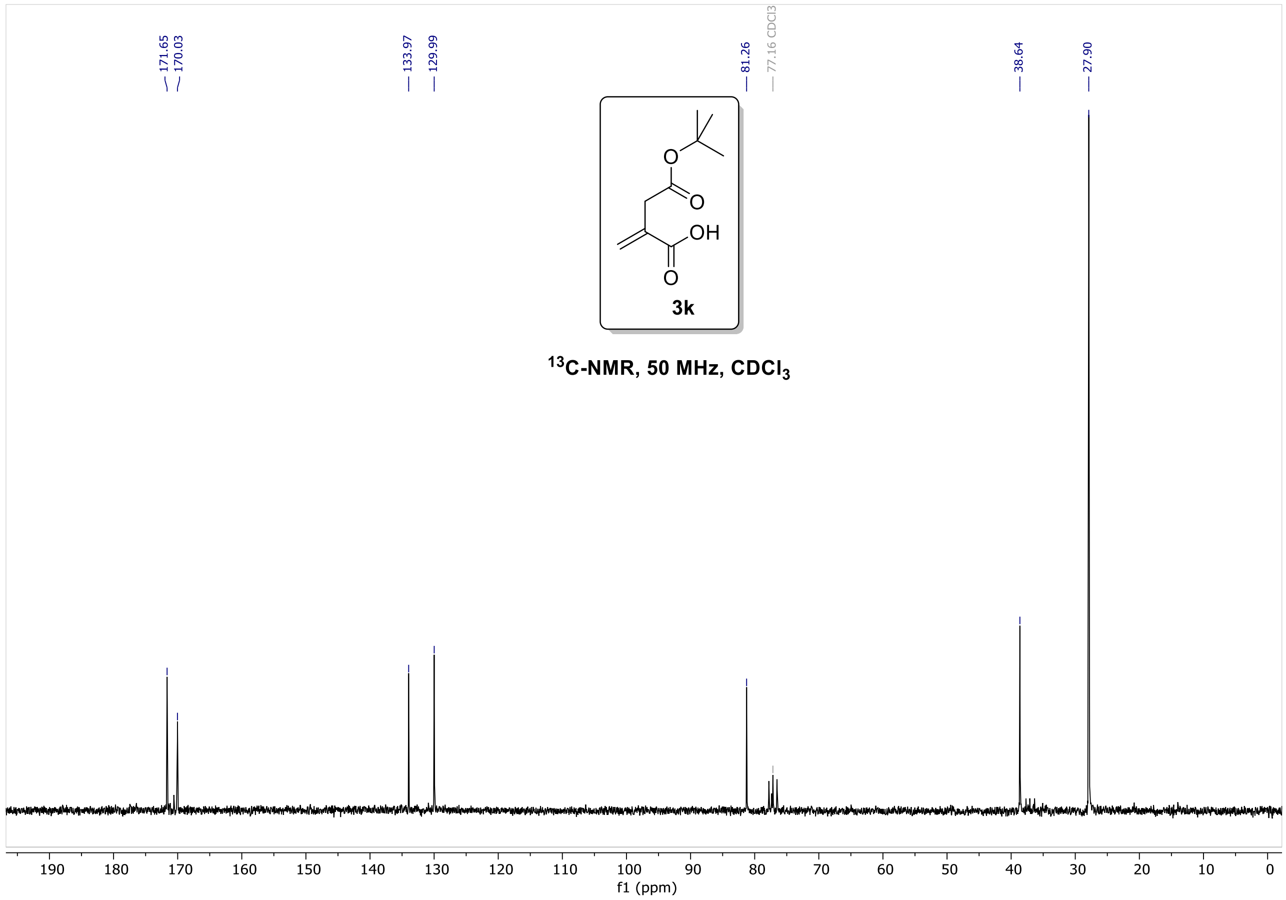
171.65
170.03
133.97
129.99
81.26
77.16 CDCl3
38.64
27.90
OH
3k
13C-NMR, 50 MHz, CDCl3
f1 (ppm)

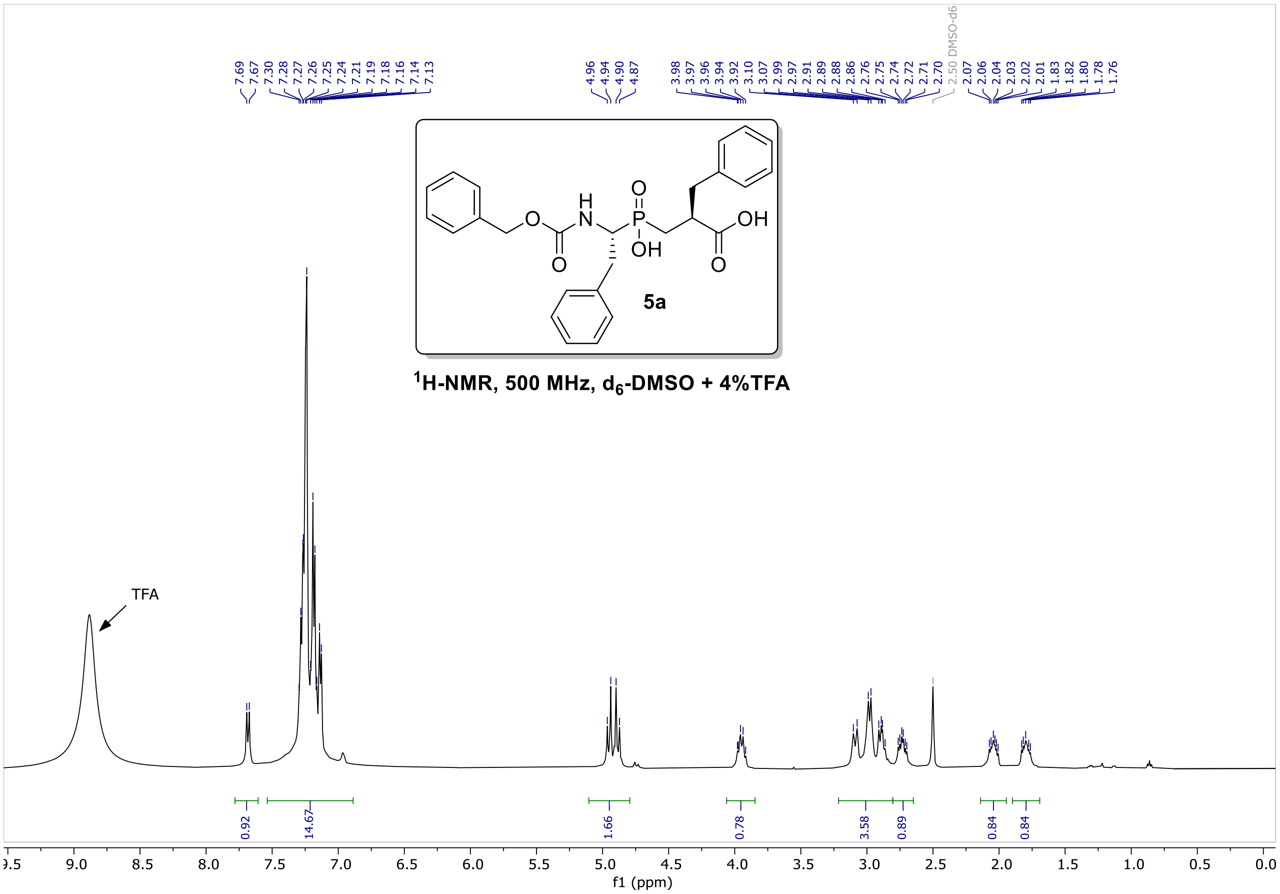
7.69 7.67 7.30 7.28 7.27 7.26 7.25 7.24 7.21 7.19 7.18 7.16 7.14 7.13
4.96 4.94 4.90 4.87
3.98 3.97 3.96 3.94 3.92 3.10 3.07 2.99 2.97 2.91 2.89 2.88 2.86 2.76 2.75 2.74 2.72 2.71 2.70
2.50 DMSO-d6
2.07 2.06 2.04 2.03 2.02 2.01 1.83 1.82 1.80 1.78 1.76
5a
^{1}H-NMR, 500 MHz, d_6-DMSO + 4%TFA
TFA
0.92 14.67 1.66 0.78 3.58 0.89 0.84 0.84
f1 (ppm)

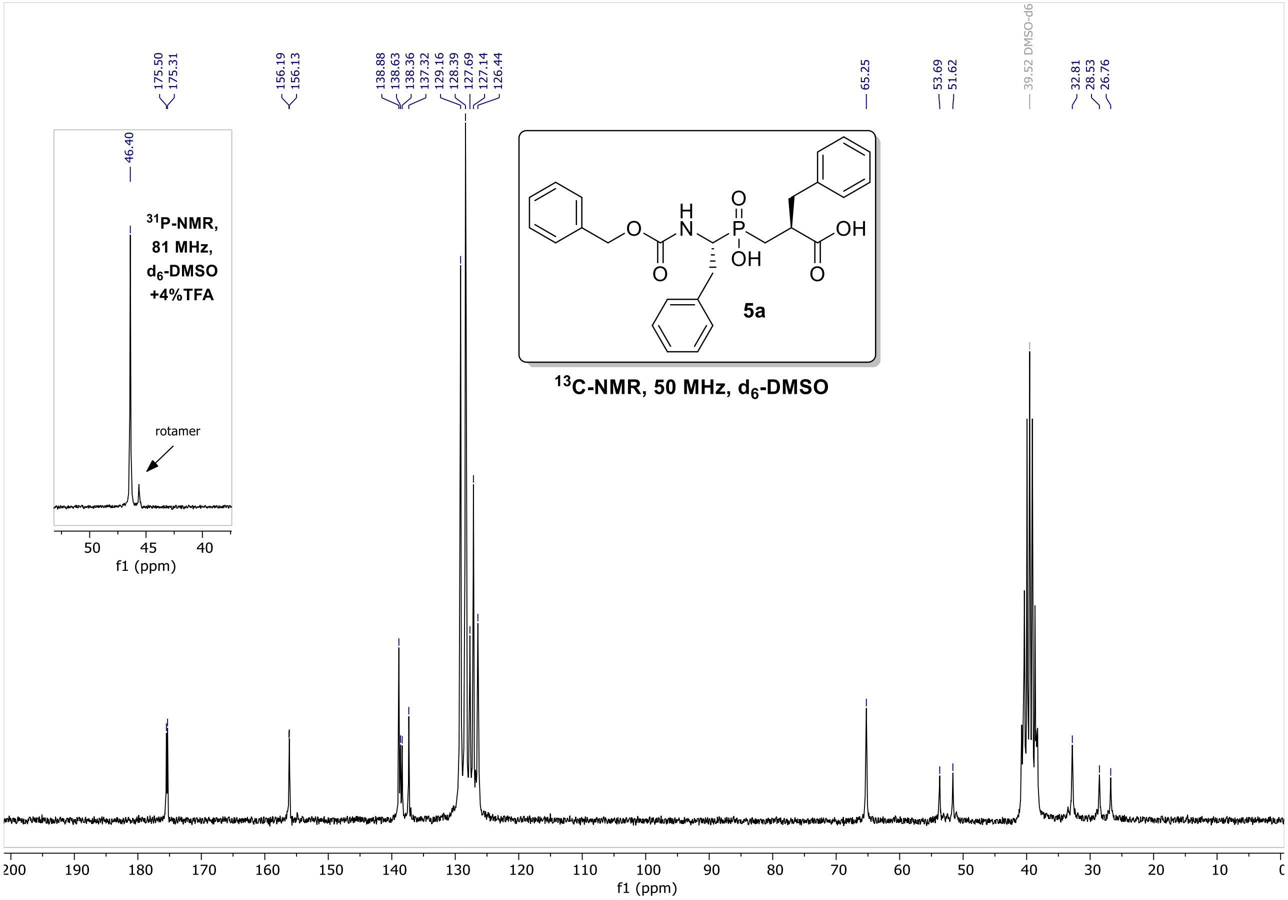
175.50 175.31
156.19 156.13
138.88 138.63 138.36 137.32 129.16 128.39 127.69 127.14 126.44
65.25
53.69 51.62
39.52 DMSO-d6
32.81 28.53 26.76
46.40
^{31}P-NMR, 81 MHz, d_6-DMSO +4%TFA
rotamer
f1 (ppm)
5a
^{13}C-NMR, 50 MHz, d_6-DMSO
f1 (ppm)

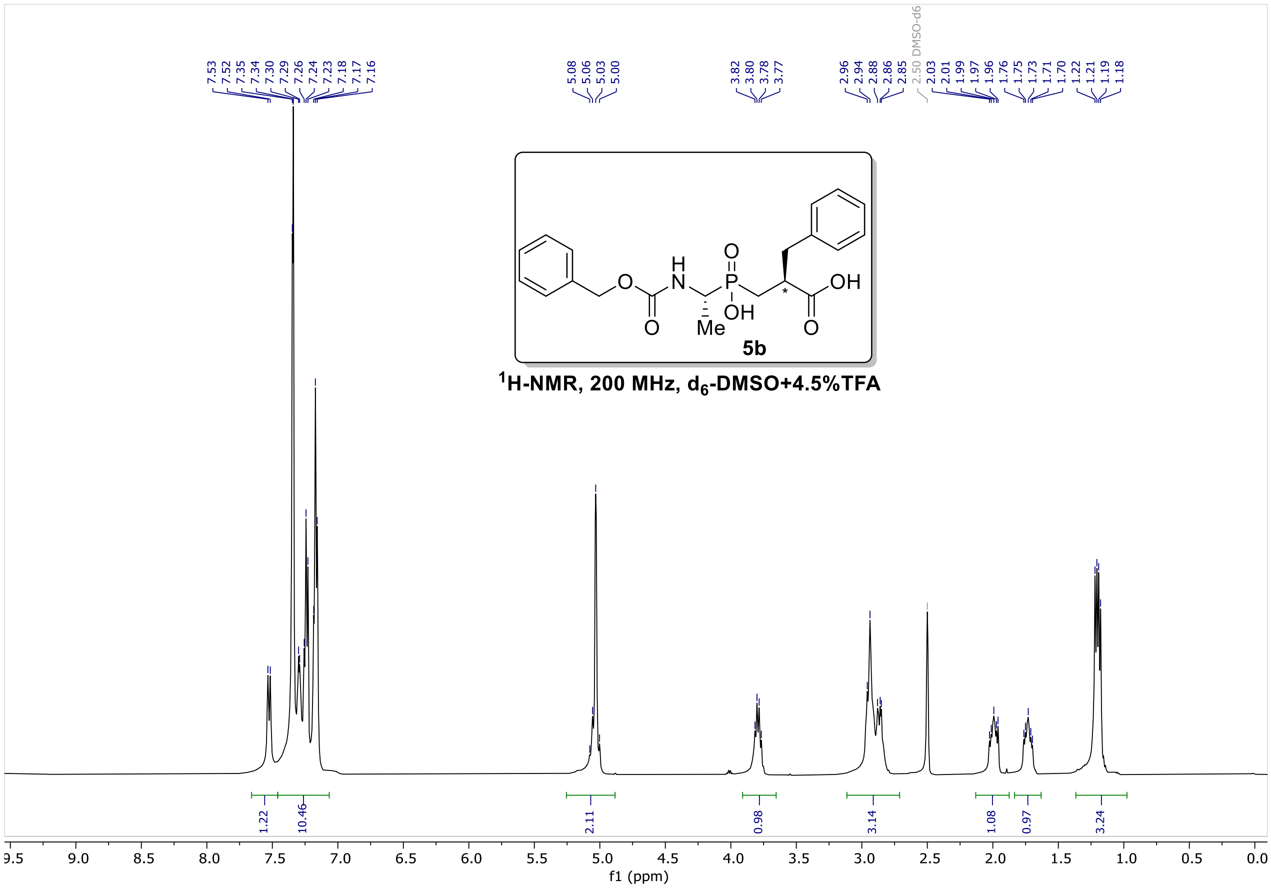

^{1}H-NMR, 200 MHz, d_6-DMSO+4.5%TFA

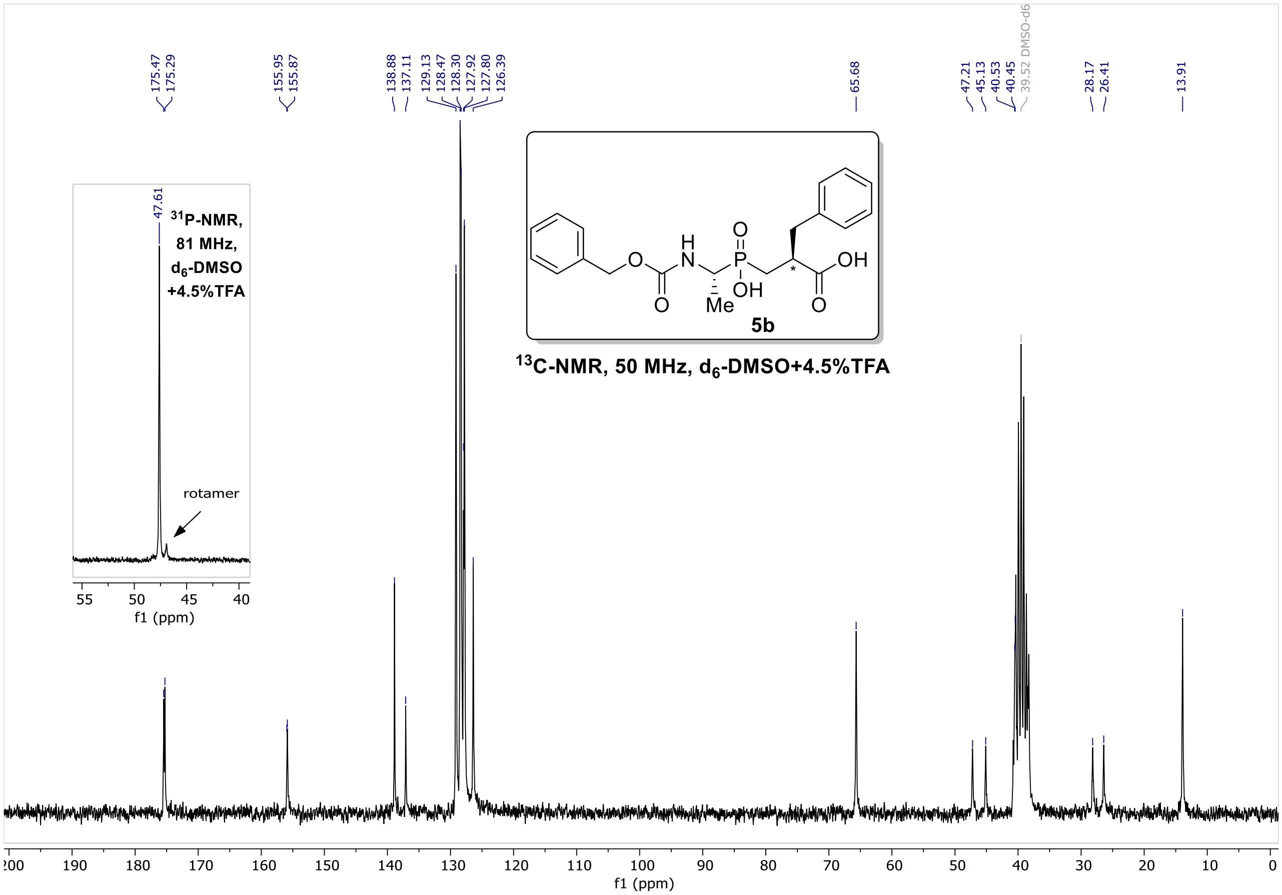

^{13}C-NMR, 50 MHz, d_6-DMSO+4.5%TFA

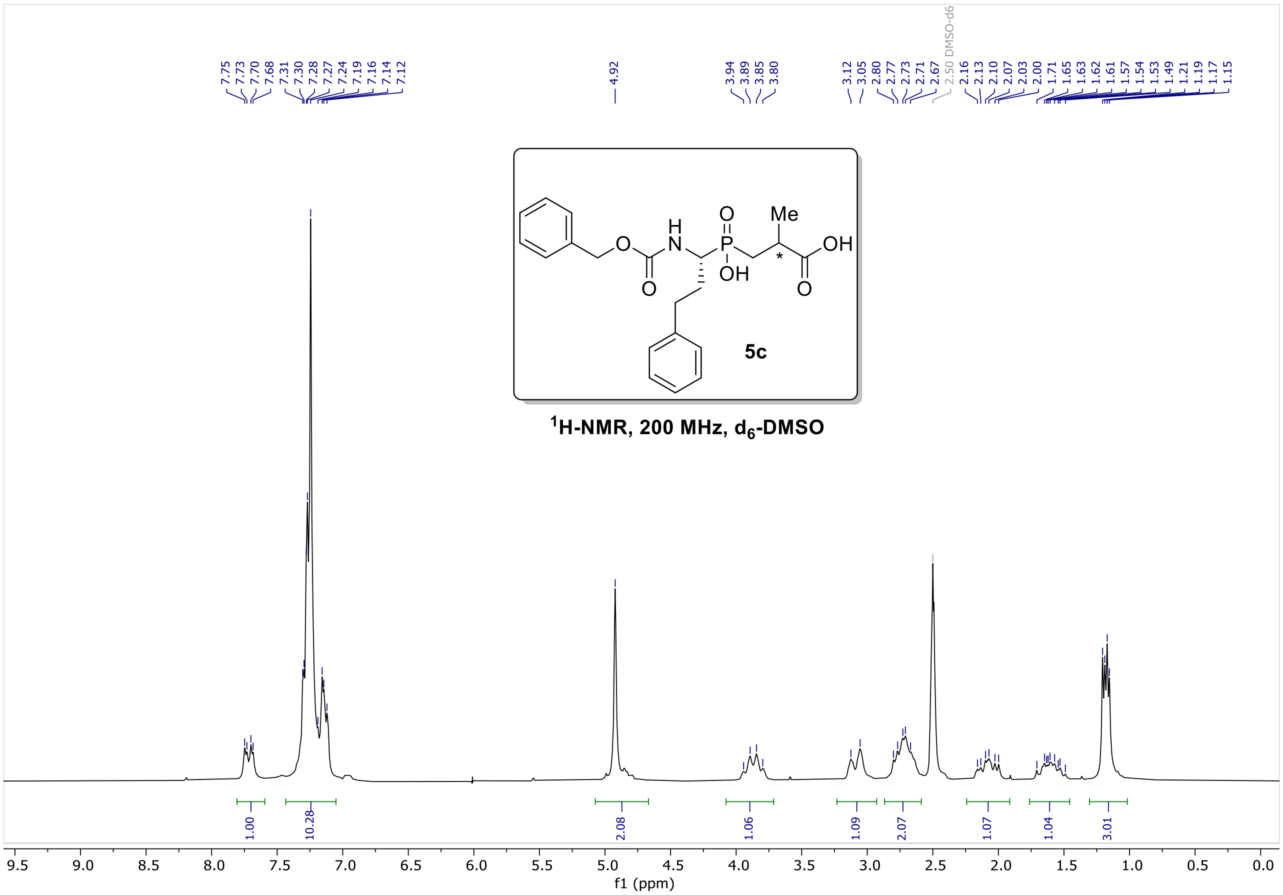
7.75 7.73 7.70 7.68 7.31 7.30 7.28 7.27 7.24 7.19 7.16 7.14 7.12
4.92
3.94 3.89 3.85 3.80
3.12 3.05 2.80 2.77 2.73 2.71 2.67 2.50 DMSO-d6 2.16 2.13 2.10 2.07 2.03 2.00 1.71 1.65 1.63 1.62 1.61 1.57 1.54 1.53 1.49 1.21 1.19 1.17 1.15
Me
O
H
N
P
OH
O
OH
O
O
*
5c
1H-NMR, 200 MHz, d6-DMSO
1.00
10.28
2.08
1.06
1.09
2.07
1.07
1.04
3.01
9.5 9.0 8.5 8.0 7.5 7.0 6.5 6.0 5.5 5.0 4.5 4.0 3.5 3.0 2.5 2.0 1.5 1.0 0.5 0.0
f1 (ppm)

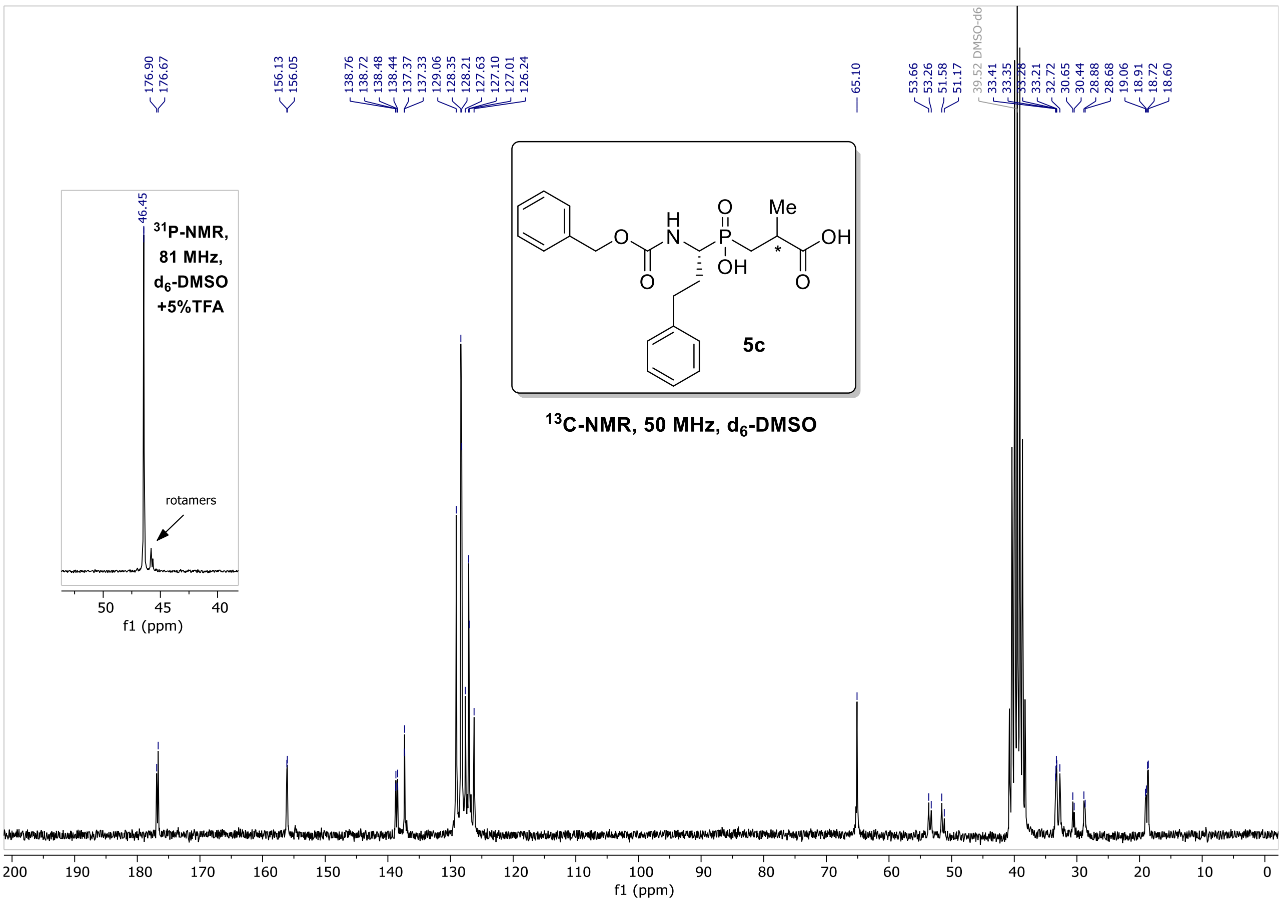
176.90 176.67
156.13 156.05
138.76 138.72 138.48 138.44 137.37 137.33 129.06 128.35 128.21 127.63 127.10 127.01 126.24
65.10
53.66 53.26 51.58 51.17 39.52 DMSO-d6 33.41 33.35 33.28 33.21 32.72 30.65 30.44 28.88 28.68 19.06 18.91 18.72 18.60
46.45
31P-NMR,
81 MHz,
d6-DMSO
+5%TFA
rotamers
50 45 40
f1 (ppm)
Me
O
H
N
P
OH
O
OH
O
O
*
5c
13C-NMR, 50 MHz, d6-DMSO
200 190 180 170 160 150 140 130 120 110 100 90 80 70 60 50 40 30 20 10 0
f1 (ppm)

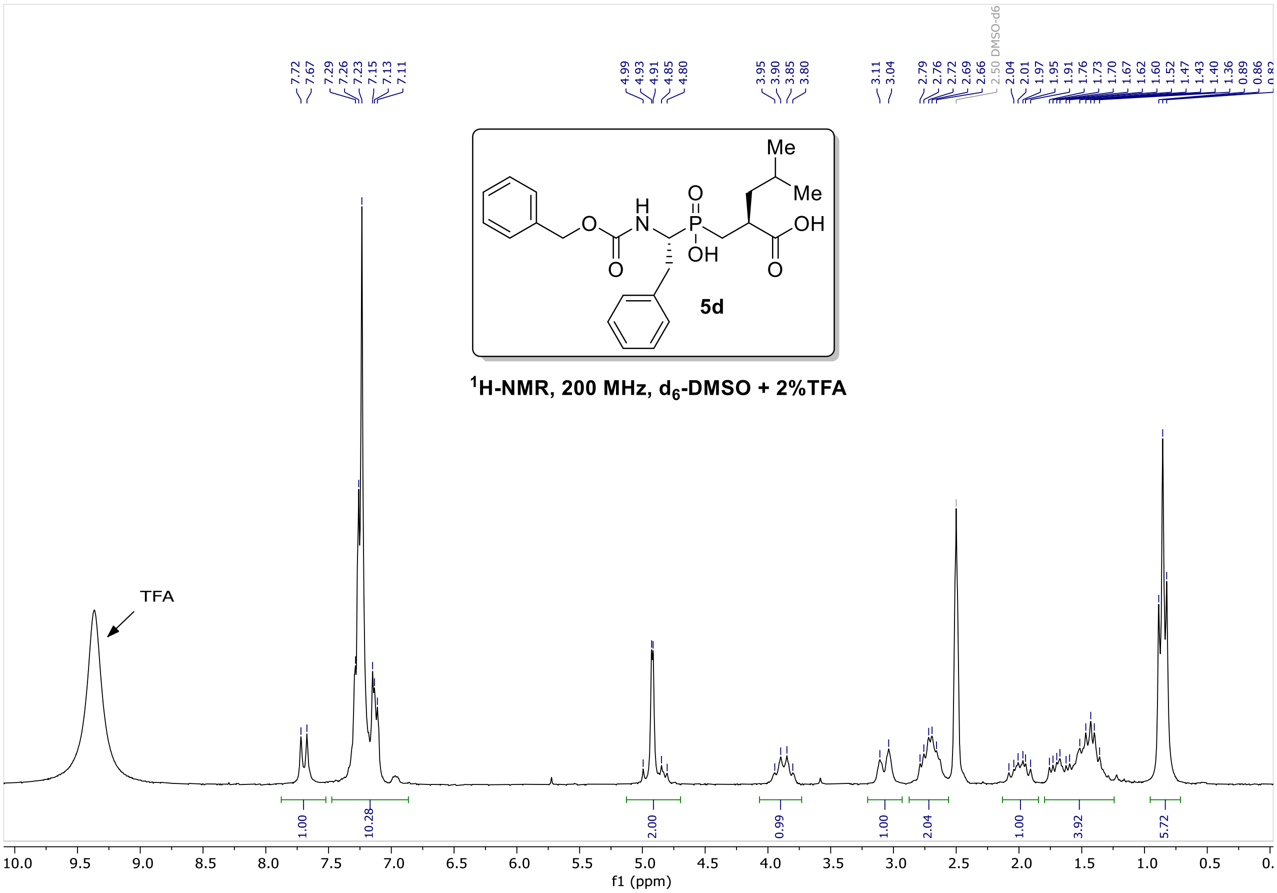
7.72 7.67 7.29 7.26 7.23 7.15 7.13 7.11
4.99 4.93 4.91 4.85 4.80
3.95 3.90 3.85 3.80
3.11 3.04
2.79 2.76 2.72 2.69 2.66
2.50 DMSO-d6
2.04 2.01 1.97 1.95 1.91 1.76 1.73 1.70 1.67 1.62 1.60 1.52 1.47 1.43 1.40 1.36 0.89 0.86
Me
Me
H
N
O
P
OH
O
OH
O
O
5d
^{1}H-NMR, 200 MHz, d_6-DMSO + 2%TFA
TFA
1.00
10.28
2.00
0.99
1.00
2.04
1.00
3.92
5.72
f1 (ppm)

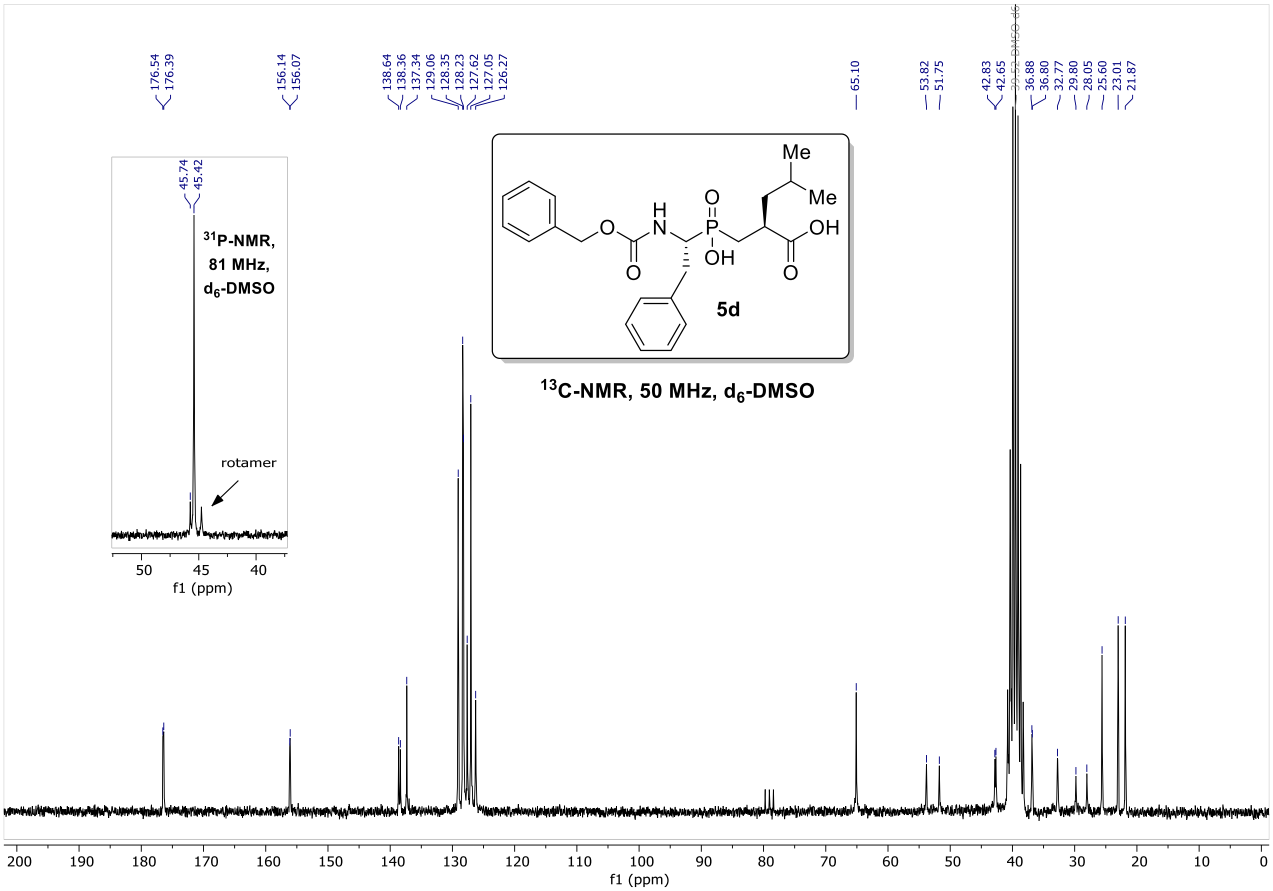
176.54 176.39
156.14 156.07
138.64 138.36 137.34 129.06 128.35 128.23 127.62 127.05 126.27
65.10
53.82 51.75
42.83 42.65
39.52 DMSO-d6
36.88 36.80 32.77 29.80 28.05 25.60 23.01 21.87
45.74 45.42
^{31}P-NMR,
81 MHz,
d_6-DMSO
rotamer
f1 (ppm)
Me
Me
H
N
O
P
OH
O
OH
O
O
5d
^{13}C-NMR, 50 MHz, d_6-DMSO
f1 (ppm)

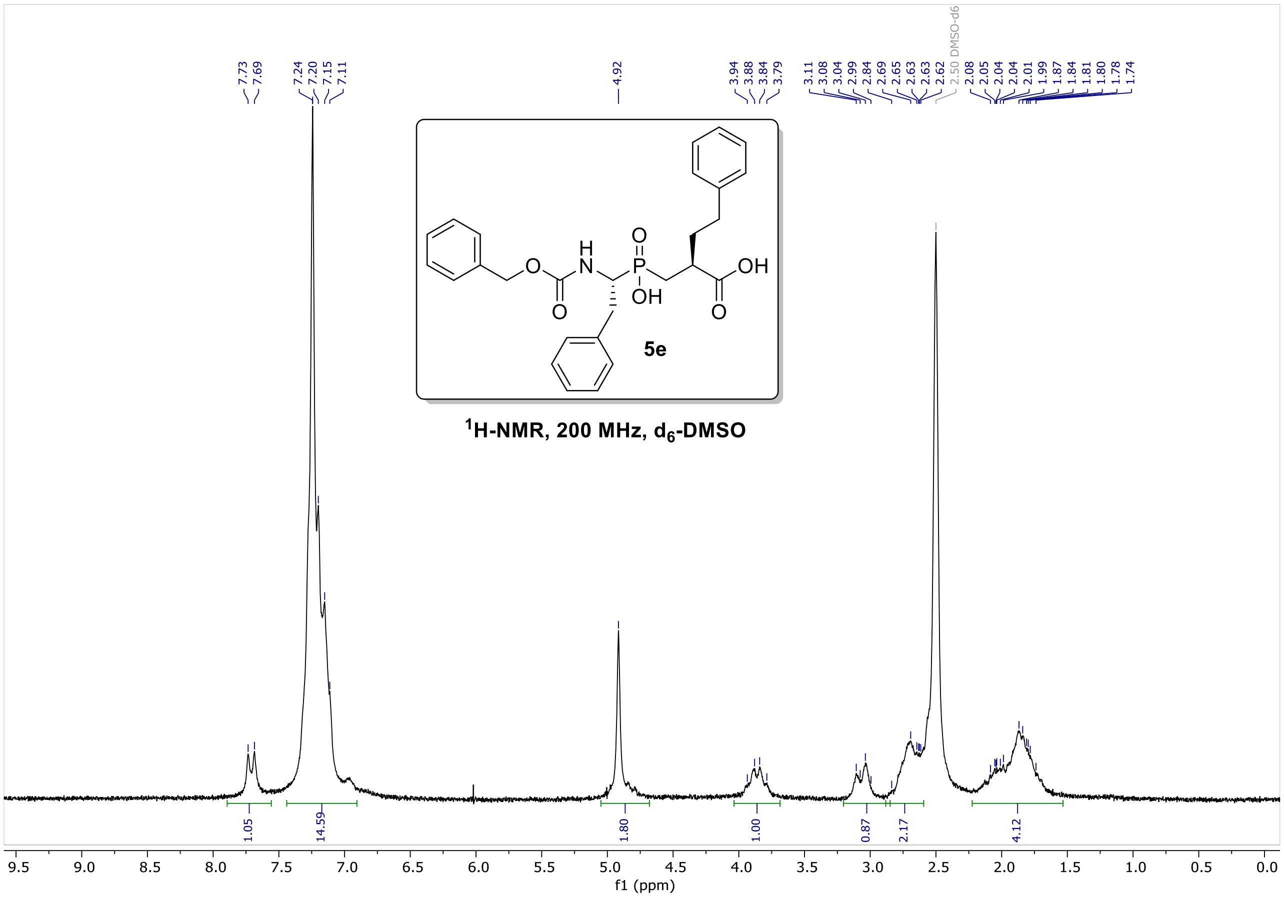
7.73
7.69
7.24
7.20
7.15
7.11
4.92
3.94
3.88
3.84
3.79
3.11
3.08
3.04
2.99
2.84
2.69
2.65
2.63
2.63
2.62
2.50 DMSO-d6
2.08
2.05
2.04
2.04
2.01
1.99
1.87
1.84
1.81
1.80
1.78
1.74
5e
1H-NMR, 200 MHz, d6-DMSO
1.05
14.59
1.80
1.00
0.87
2.17
4.12
f1 (ppm)

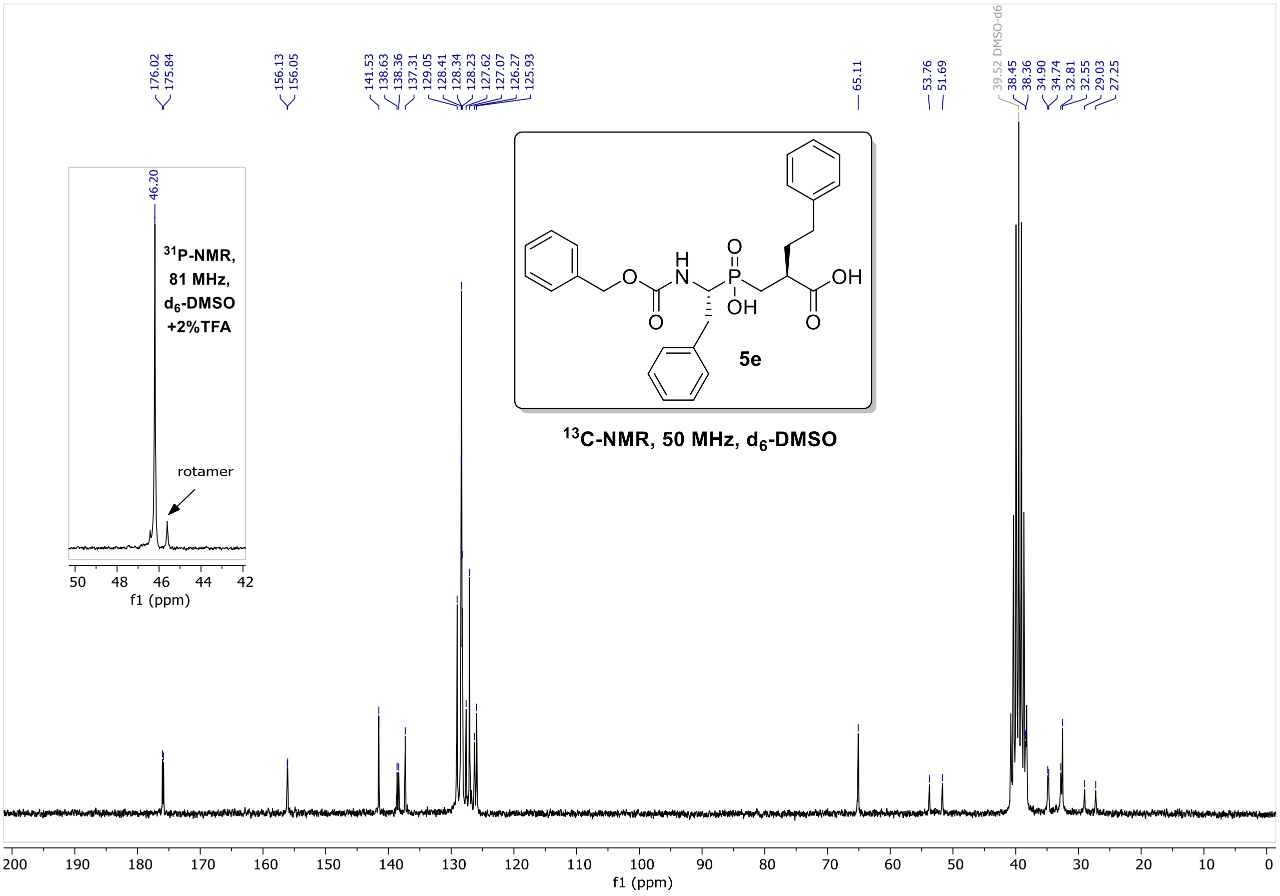
176.02
175.84
156.13
156.05
141.53
138.63
138.36
137.31
129.05
128.41
128.34
128.23
127.62
127.07
126.27
125.93
65.11
53.76
51.69
39.52 DMSO-d6
38.45
38.36
34.90
34.74
32.81
32.55
29.03
27.25
46.20
31P-NMR, 81 MHz, d6-DMSO +2%TFA
rotamer
5e
13C-NMR, 50 MHz, d6-DMSO
f1 (ppm)

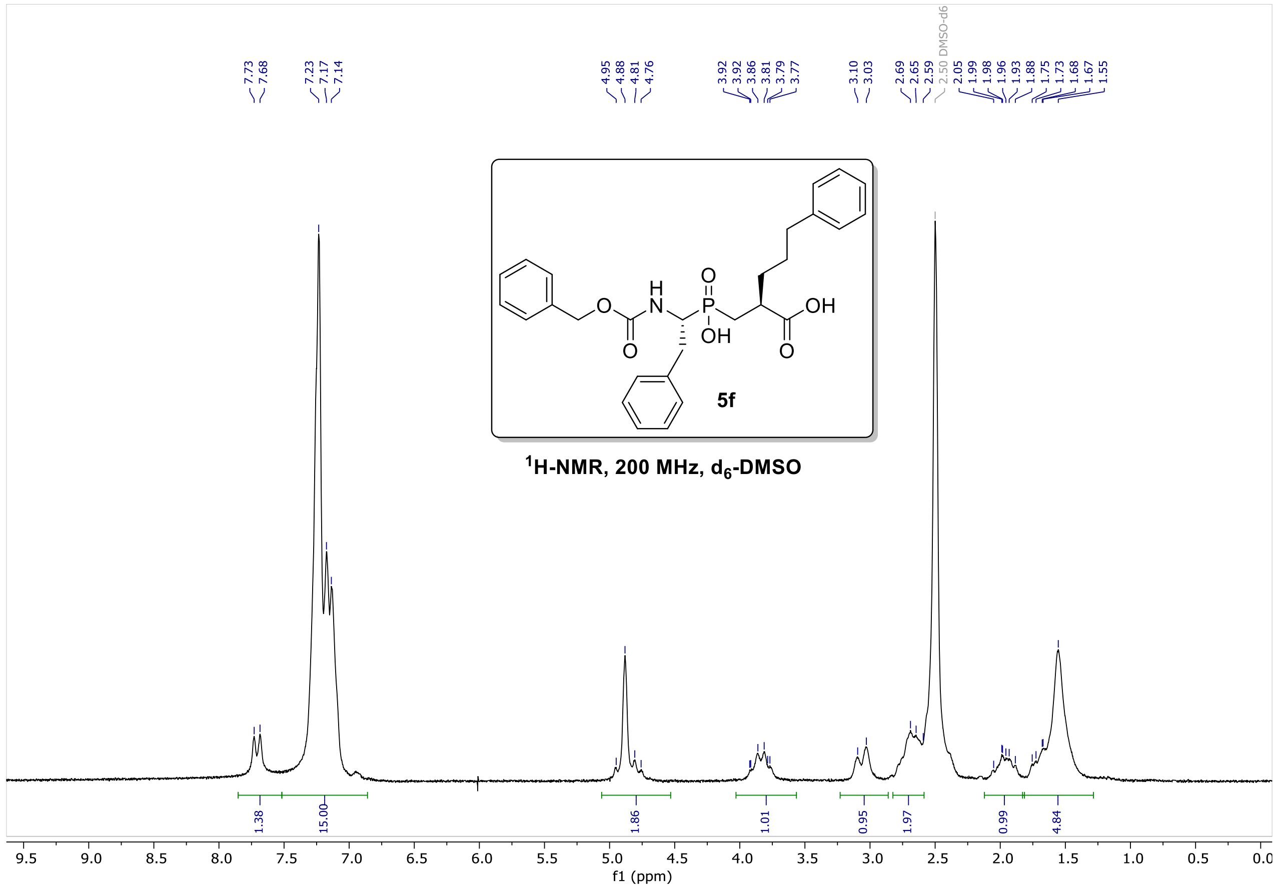
5f
1H-NMR, 200 MHz, d6-DMSO
f1 (ppm)

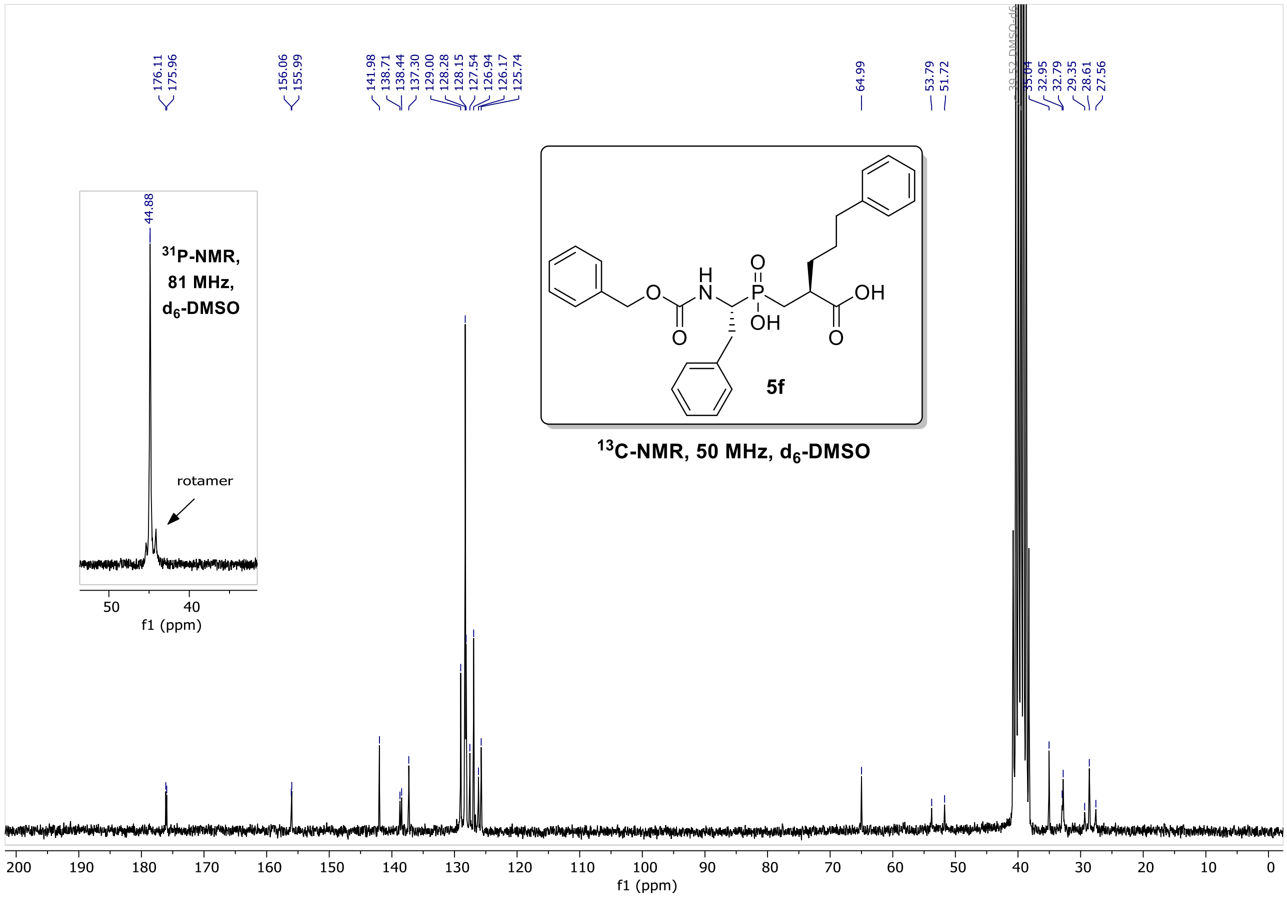
31P-NMR, 81 MHz, d6-DMSO
rotamer
5f
13C-NMR, 50 MHz, d6-DMSO
f1 (ppm)

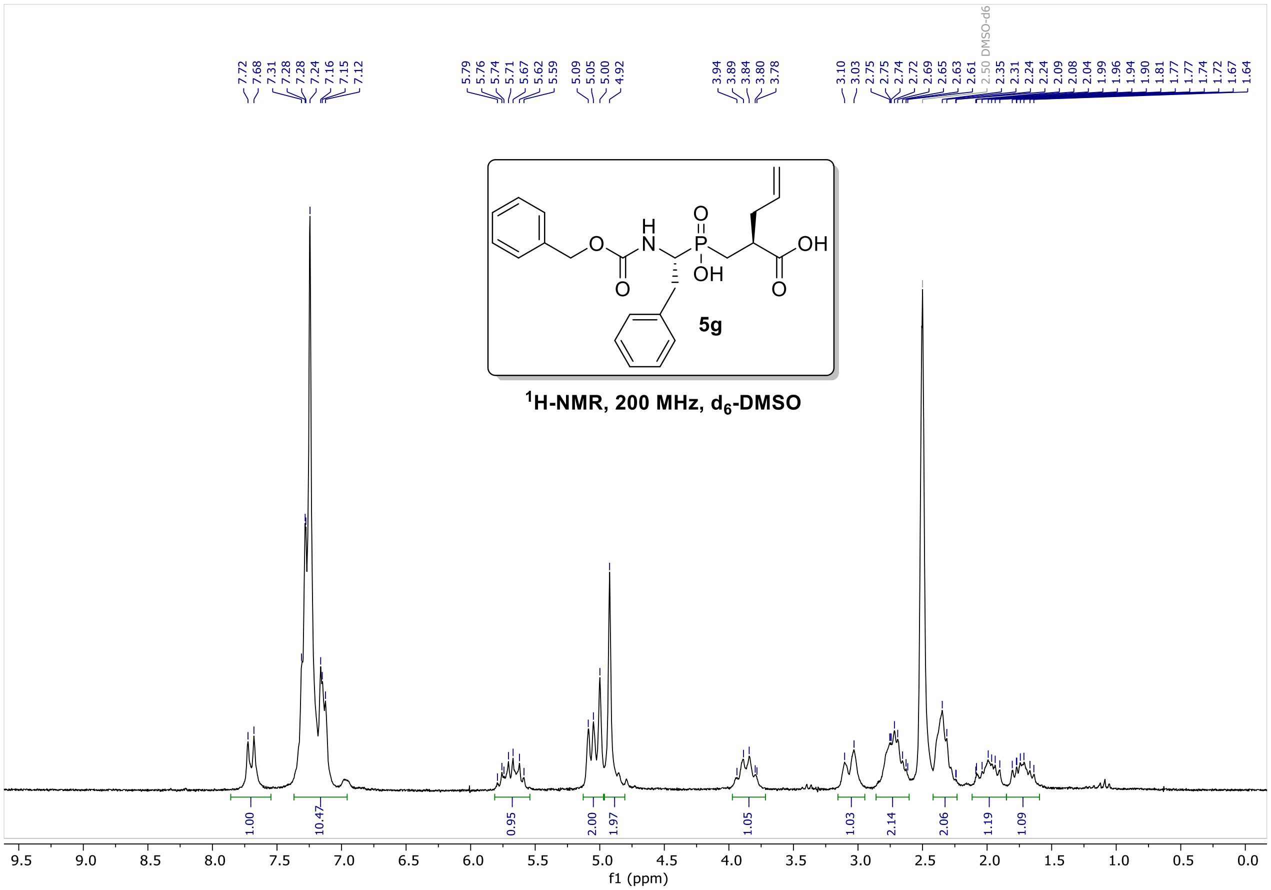
5g
^{1}H-NMR, 200 MHz, d$_6$-DMSO
f1 (ppm)

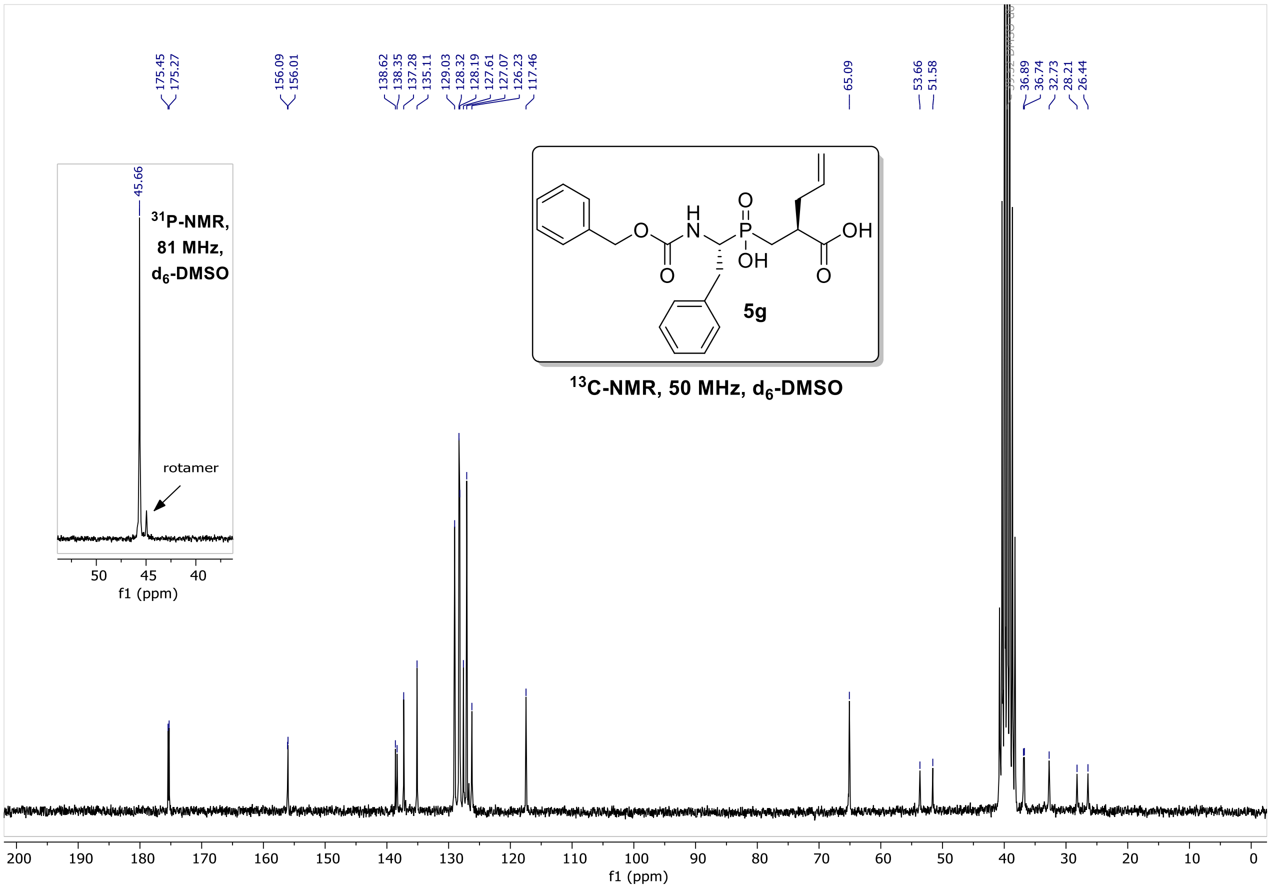
5g
^{13}C-NMR, 50 MHz, d$_6$-DMSO
^{31}P-NMR, 81 MHz, d$_6$-DMSO
rotamer
f1 (ppm)

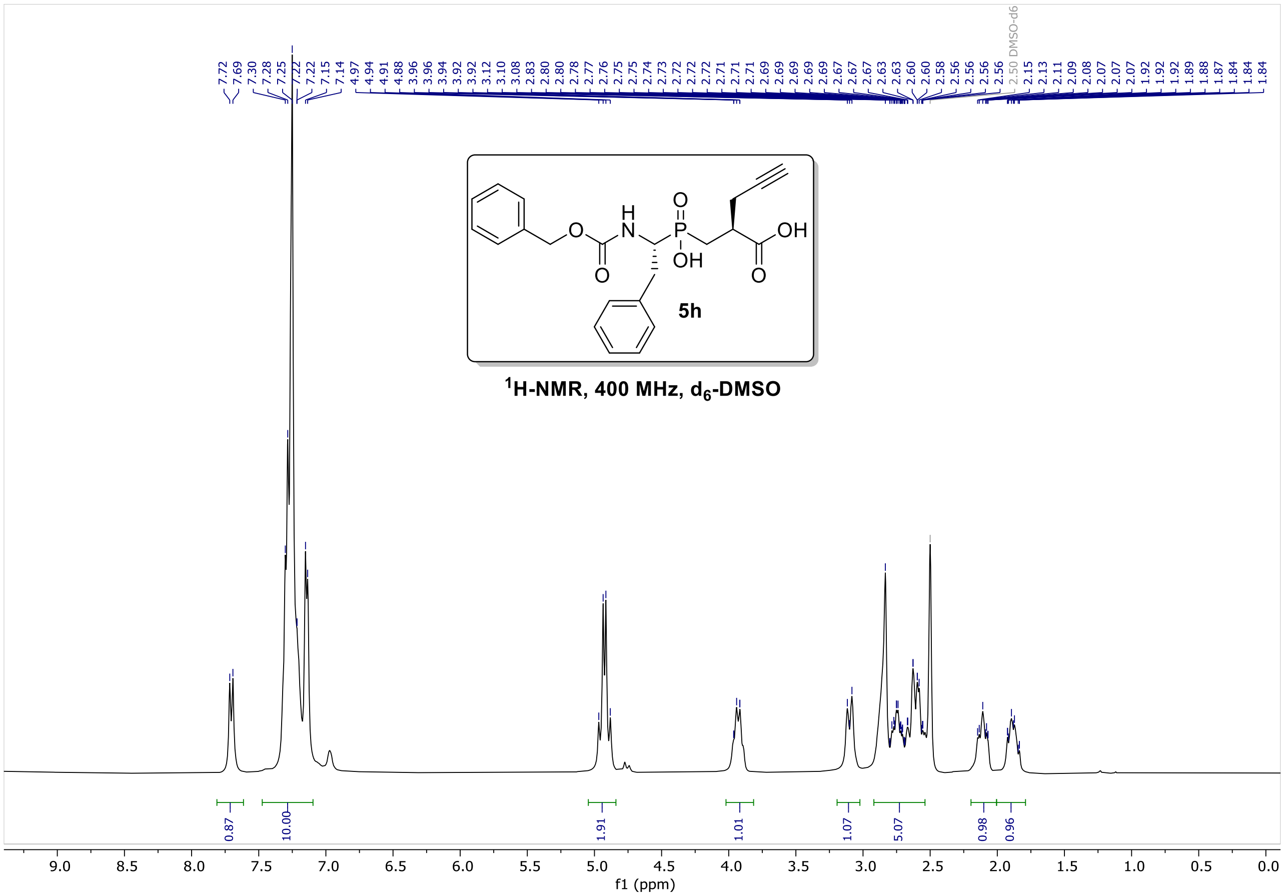
5h
1H-NMR, 400 MHz, d6-DMSO
f1 (ppm)

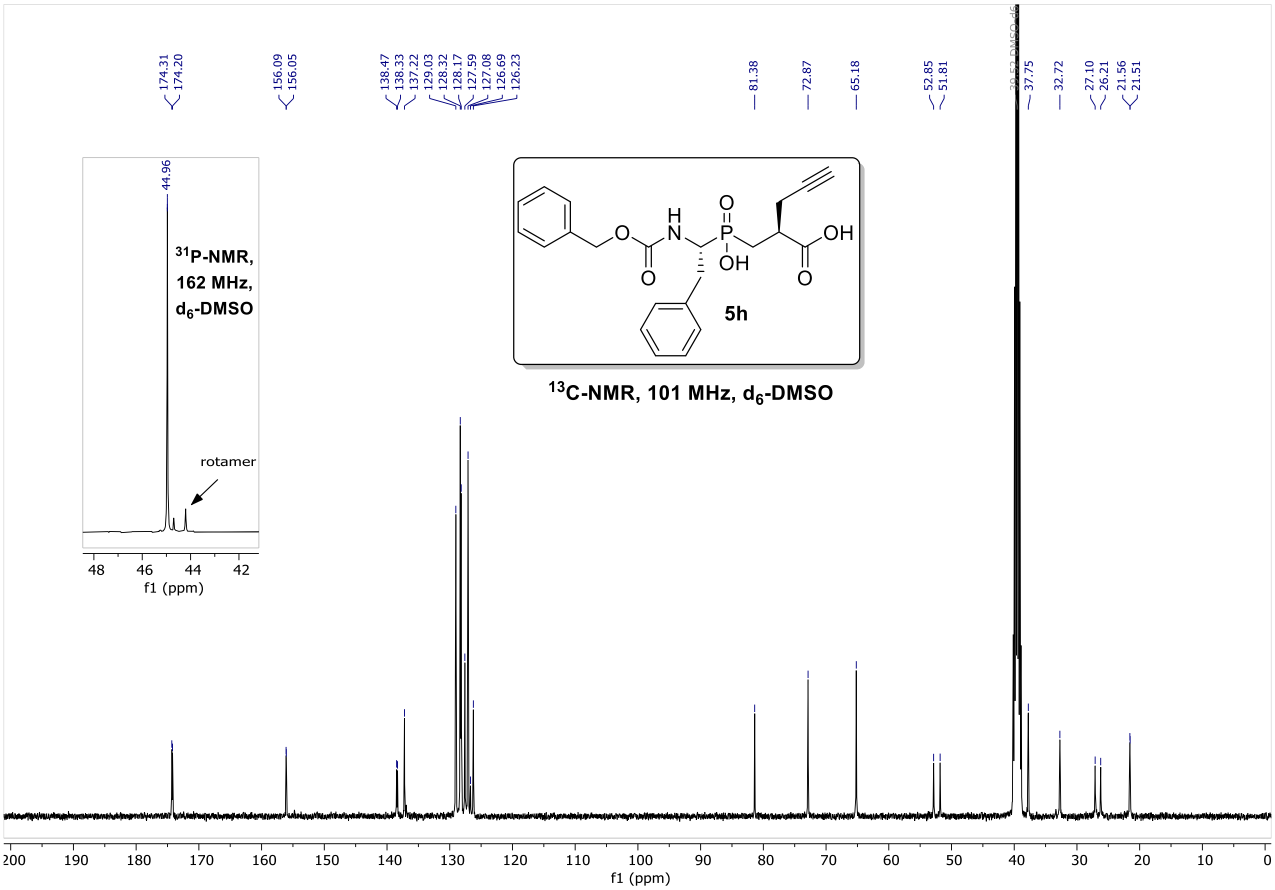
5h
13C-NMR, 101 MHz, d6-DMSO
31P-NMR, 162 MHz, d6-DMSO
rotamer
f1 (ppm)

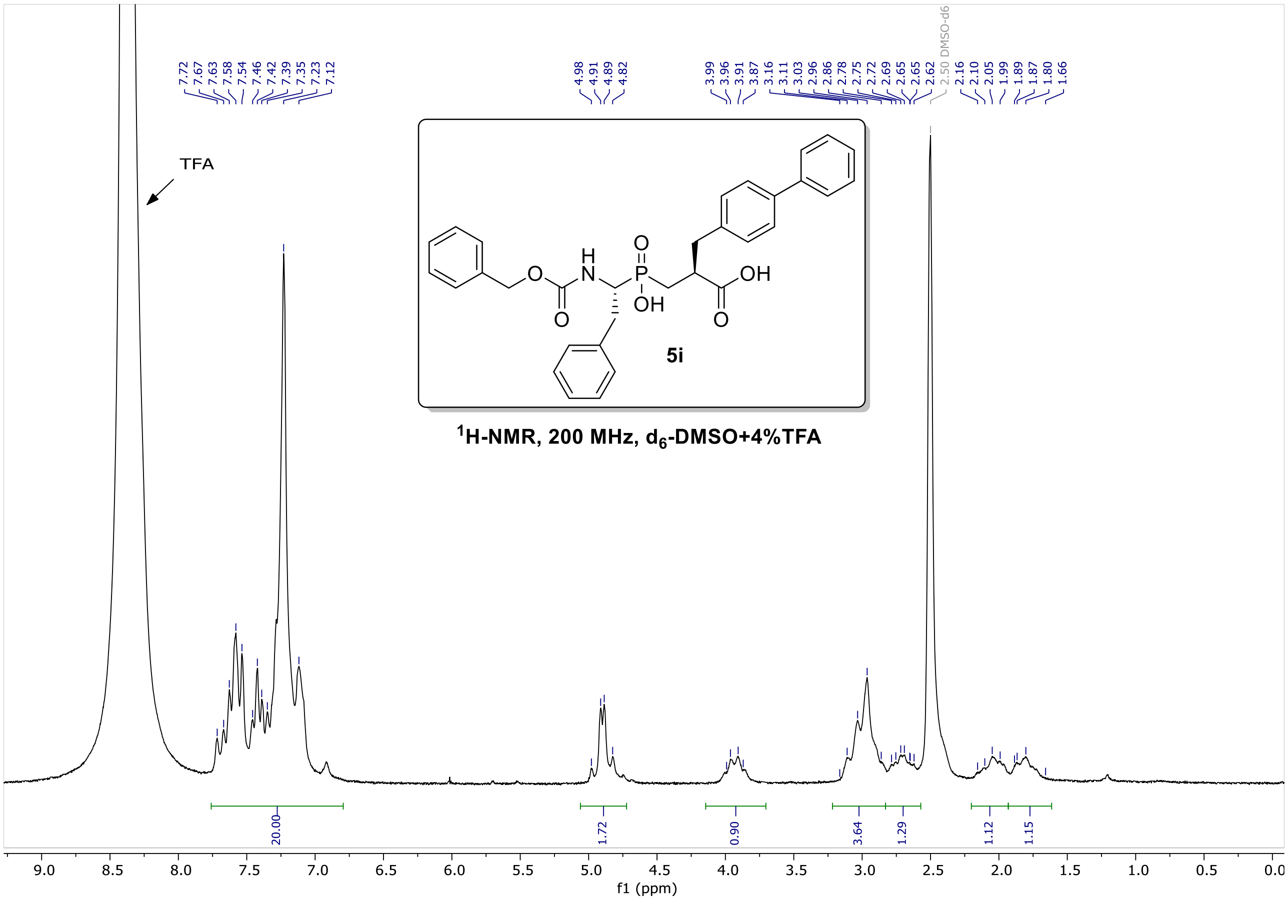
7.72 7.67 7.63 7.58 7.54 7.46 7.42 7.39 7.35 7.23 7.12
4.98 4.91 4.89 4.82
3.99 3.96 3.91 3.87 3.16 3.11 3.03 2.96 2.86 2.78 2.75 2.72 2.69 2.65 2.65 2.62
2.50 DMSO-d6
2.16 2.10 2.05 1.99 1.89 1.87 1.80 1.66
TFA
H
N
O
P
OH
5i
¹H-NMR, 200 MHz, d₆-DMSO+4%TFA
20.00
1.72
0.90
3.64
1.29
1.12
1.15
9.0 8.5 8.0 7.5 7.0 6.5 6.0 5.5 5.0 4.5 4.0 3.5 3.0 2.5 2.0 1.5 1.0 0.5 0.0
f1 (ppm)

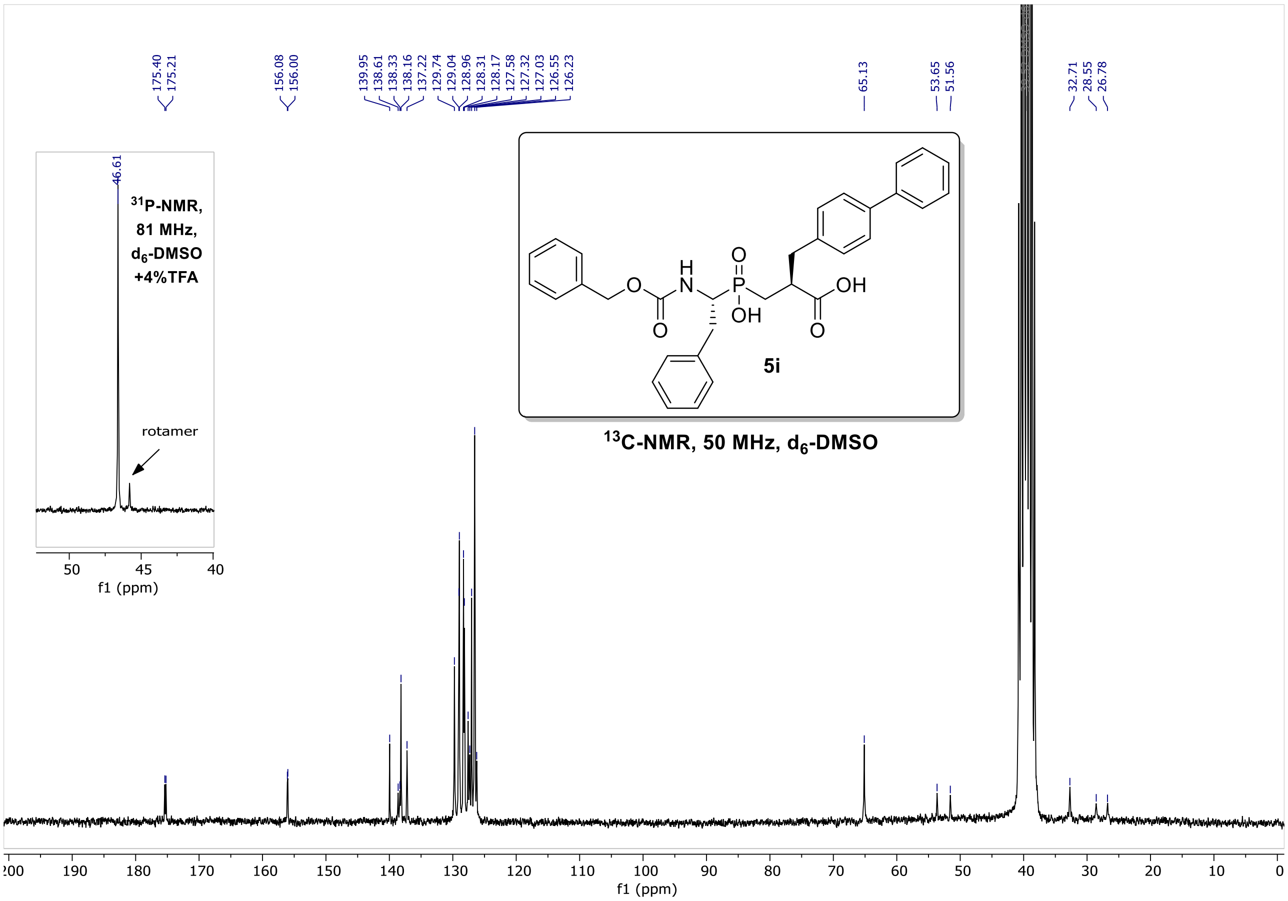
175.40 175.21
156.08 156.00
139.95 138.61 138.33 138.16 137.22 129.74 129.04 128.96 128.31 128.17 127.58 127.32 127.03 126.55 126.23
65.13
53.65 51.56
32.71 28.55 26.78
46.61
³¹P-NMR, 81 MHz, d₆-DMSO +4%TFA
rotamer
50 45 40
f1 (ppm)
H
N
O
P
OH
5i
¹³C-NMR, 50 MHz, d₆-DMSO
200 190 180 170 160 150 140 130 120 110 100 90 80 70 60 50 40 30 20 10 0
f1 (ppm)

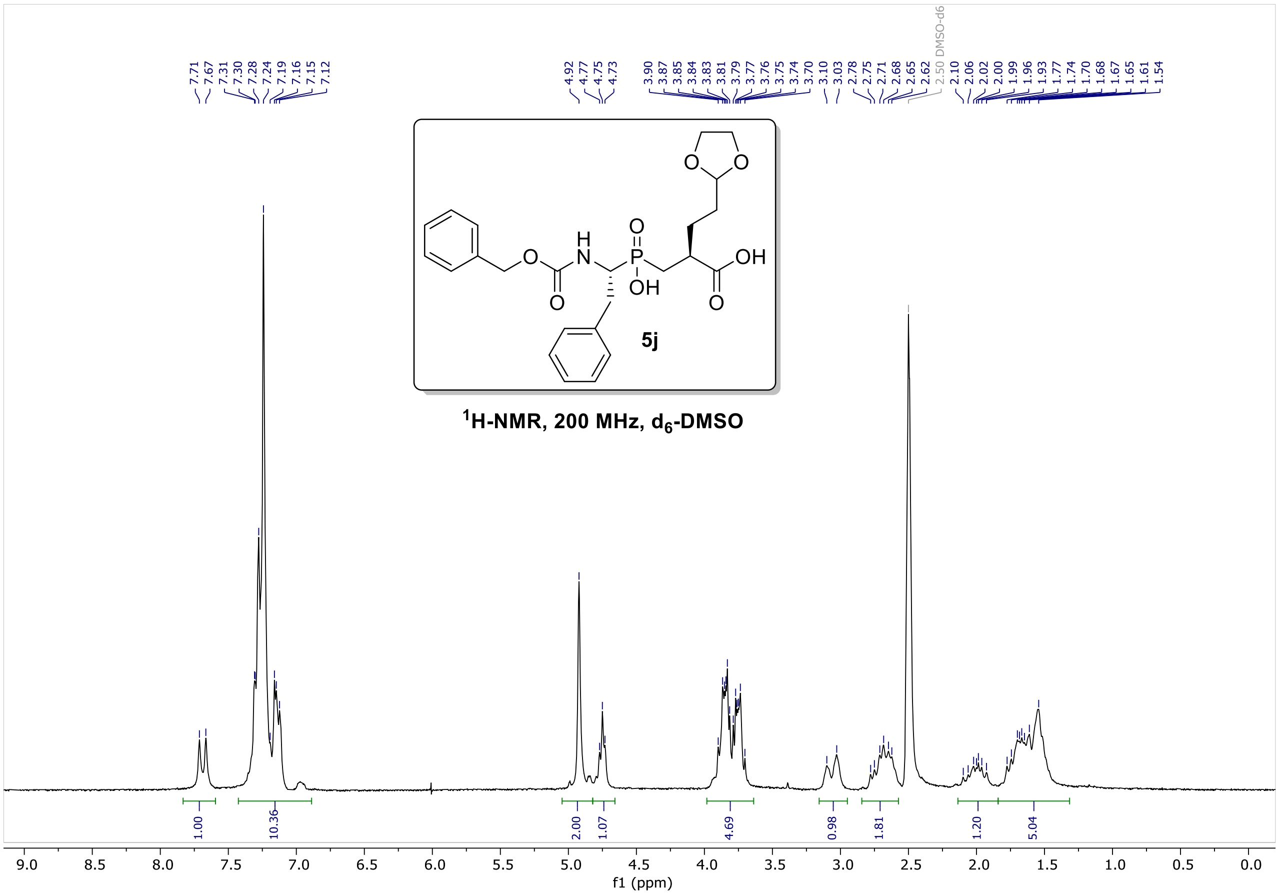

^{1}H-NMR, 200 MHz, d_6-DMSO

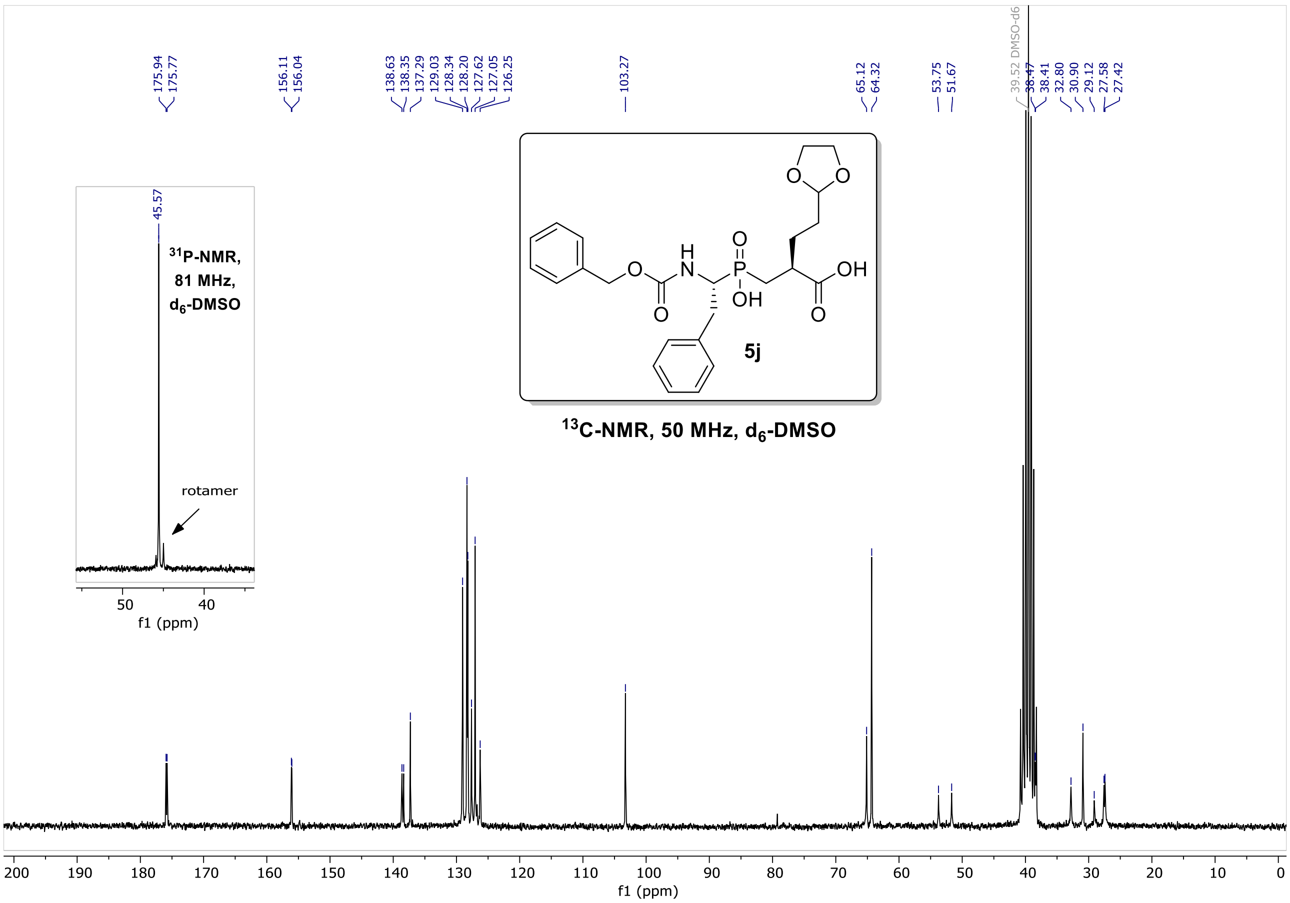

^{13}C-NMR, 50 MHz, d_6-DMSO

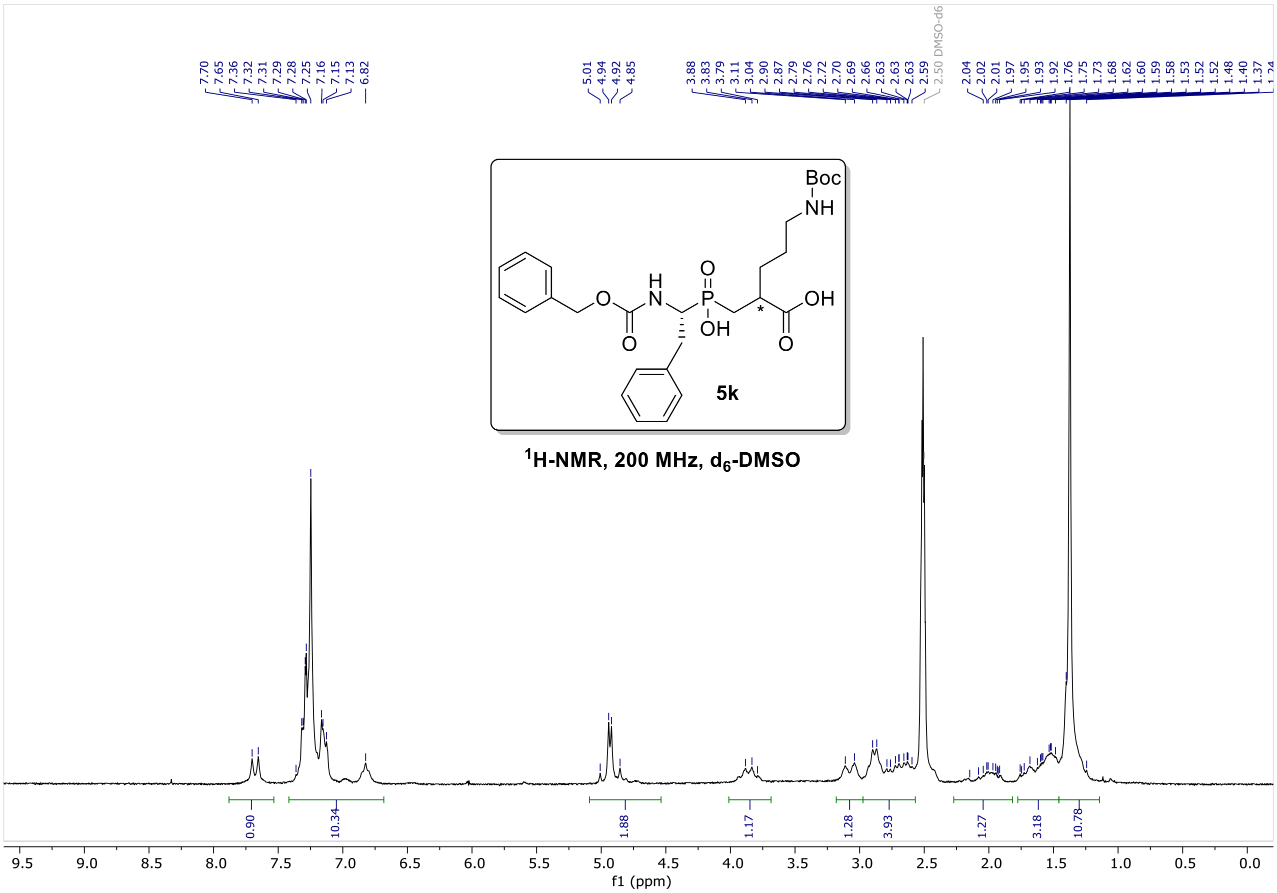
7.70 7.65 7.36 7.32 7.31 7.29 7.28 7.25 7.16 7.15 7.13 6.82
5.01 4.94 4.92 4.85
3.88 3.83 3.79 3.11 3.04 2.90 2.87 2.79 2.76 2.72 2.70 2.69 2.66 2.63 2.63 2.63 2.59
2.50 DMSO-d6
2.04 2.02 2.01 1.97 1.95 1.93 1.92 1.76 1.75 1.73 1.68 1.62 1.60 1.59 1.58 1.53 1.52 1.52 1.48 1.40 1.37
Boc
NH
O
H
N
P
OH
O
OH
O
*
5k
^{1}H-NMR, 200 MHz, d_6-DMSO
0.90
10.34
1.88
1.17
1.28
3.93
1.27
3.18
10.78
f1 (ppm)

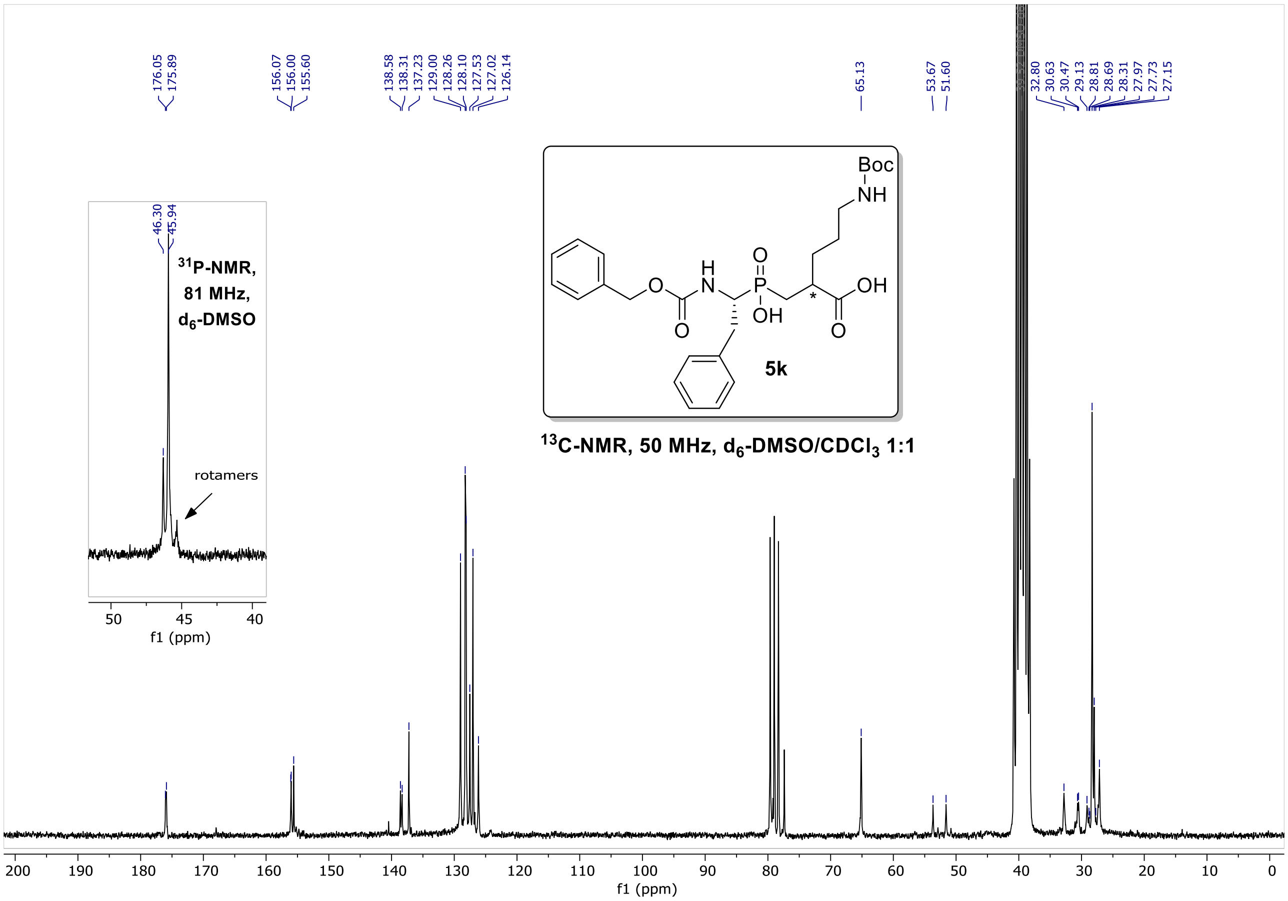
176.05 175.89
156.07 156.00 155.60
138.58 138.31 137.23 129.00 128.26 128.10 127.53 127.02 126.14
65.13
53.67 51.60
32.80 30.63 30.47 29.13 28.81 28.69 28.31 27.97 27.73 27.15
46.30 45.94
^{31}P-NMR,
81 MHz,
d_6-DMSO
rotamers
f1 (ppm)
Boc
NH
O
H
N
P
OH
O
OH
O
*
5k
^{13}C-NMR, 50 MHz, d_6-DMSO/$CDCl_3$ 1:1
f1 (ppm)

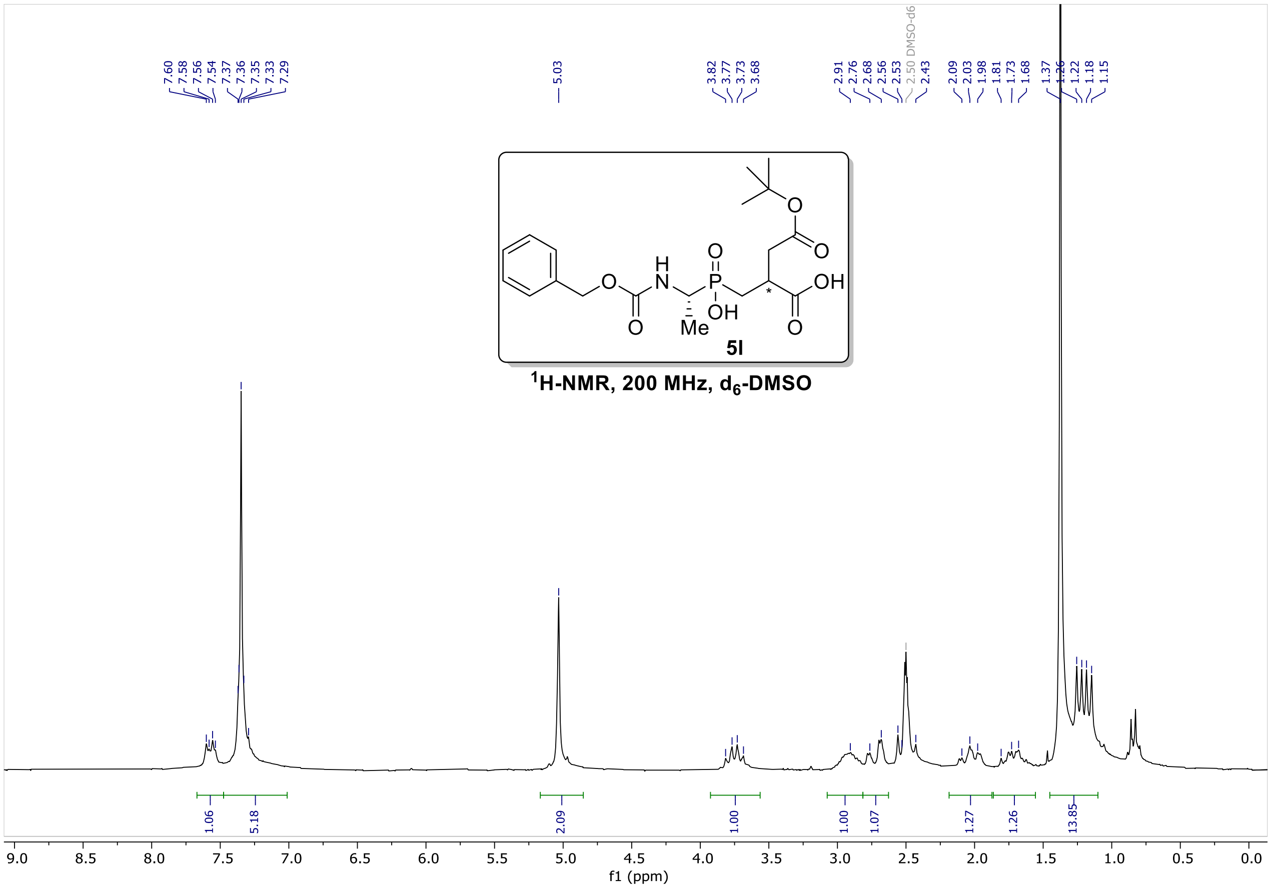

7.60 7.58 7.56 7.54 7.37 7.36 7.35 7.33 7.29
5.03
3.82 3.77 3.73 3.68
2.91 2.76 2.68 2.56 2.53 2.50 DMSO-d6 2.43
2.09 2.03 1.98 1.81 1.73 1.68
1.37 1.26 1.22 1.18 1.15
5l
^{1}H-NMR, 200 MHz, d_6-DMSO
1.06 5.18 2.09 1.00 1.00 1.07 1.27 1.26 13.85
f1 (ppm)

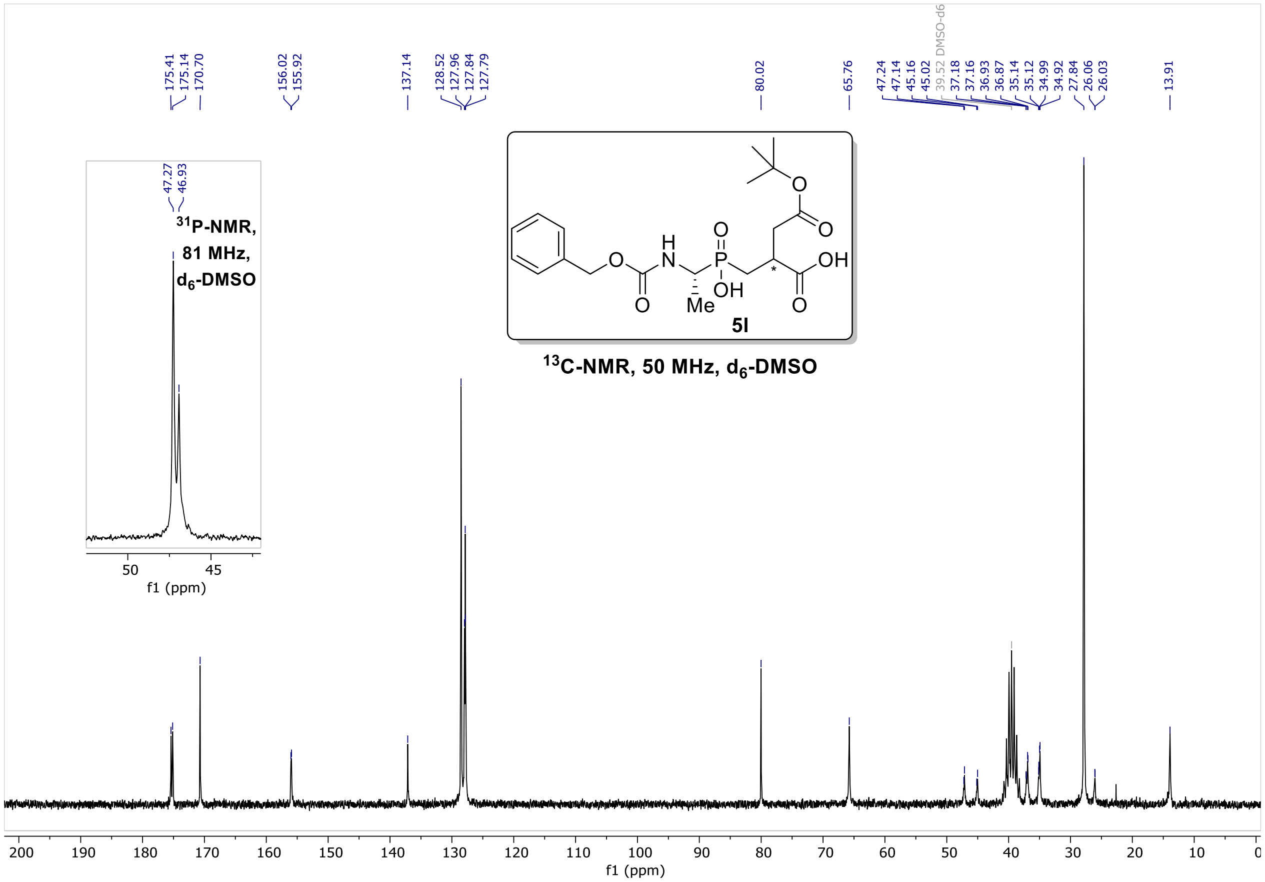

175.41 175.14 170.70
156.02 155.92
137.14
128.52 127.96 127.84 127.79
80.02
65.76
47.24 47.14 45.16 45.02 39.52 DMSO-d6 37.18 37.16 36.93 36.87 35.14 35.12 34.99 34.92
27.84 26.06 26.03
13.91
47.27 46.93
^{31}P-NMR, 81 MHz, d_6-DMSO
5l
^{13}C-NMR, 50 MHz, d_6-DMSO
f1 (ppm)

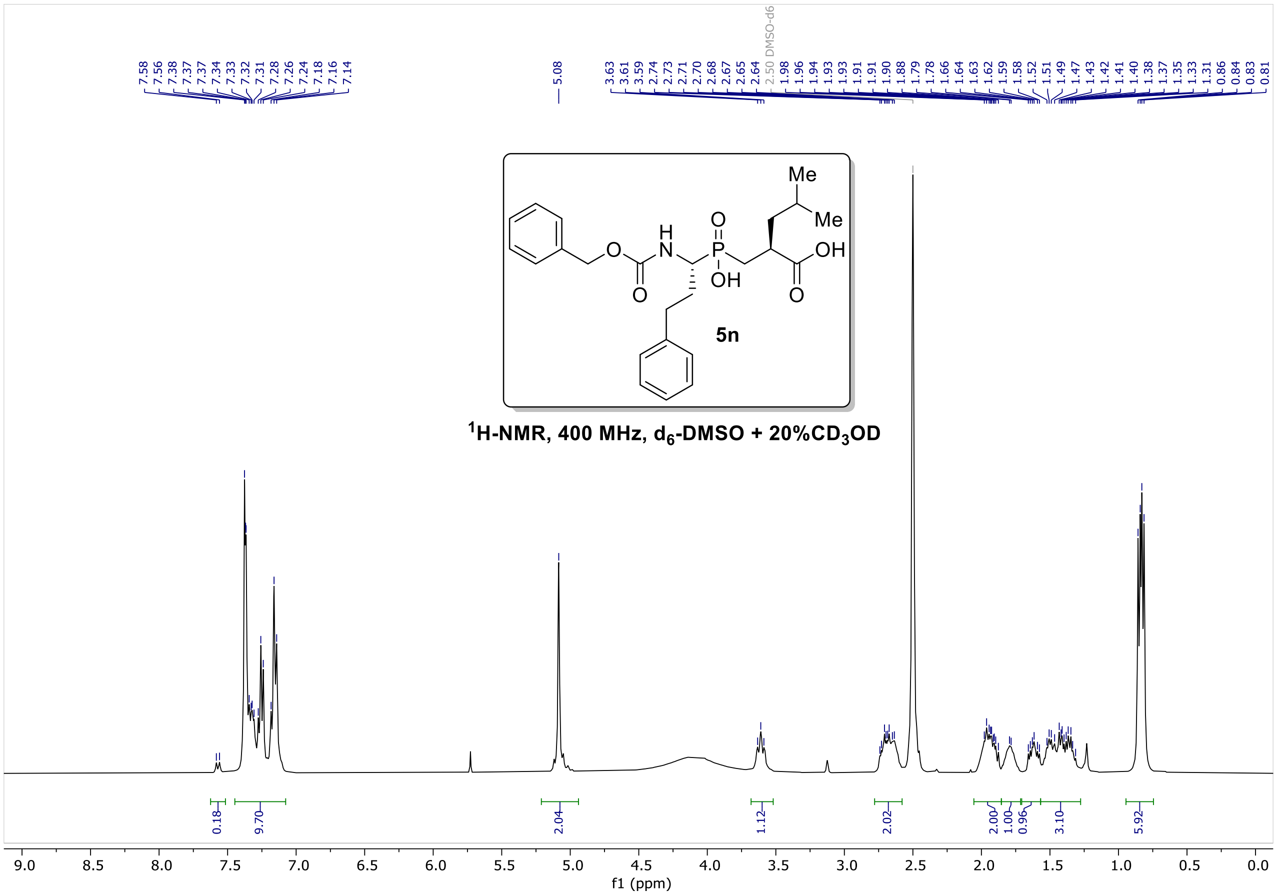

5n
^{1}H-NMR, 400 MHz, d_6-DMSO + 20%CD_3OD
f1 (ppm)

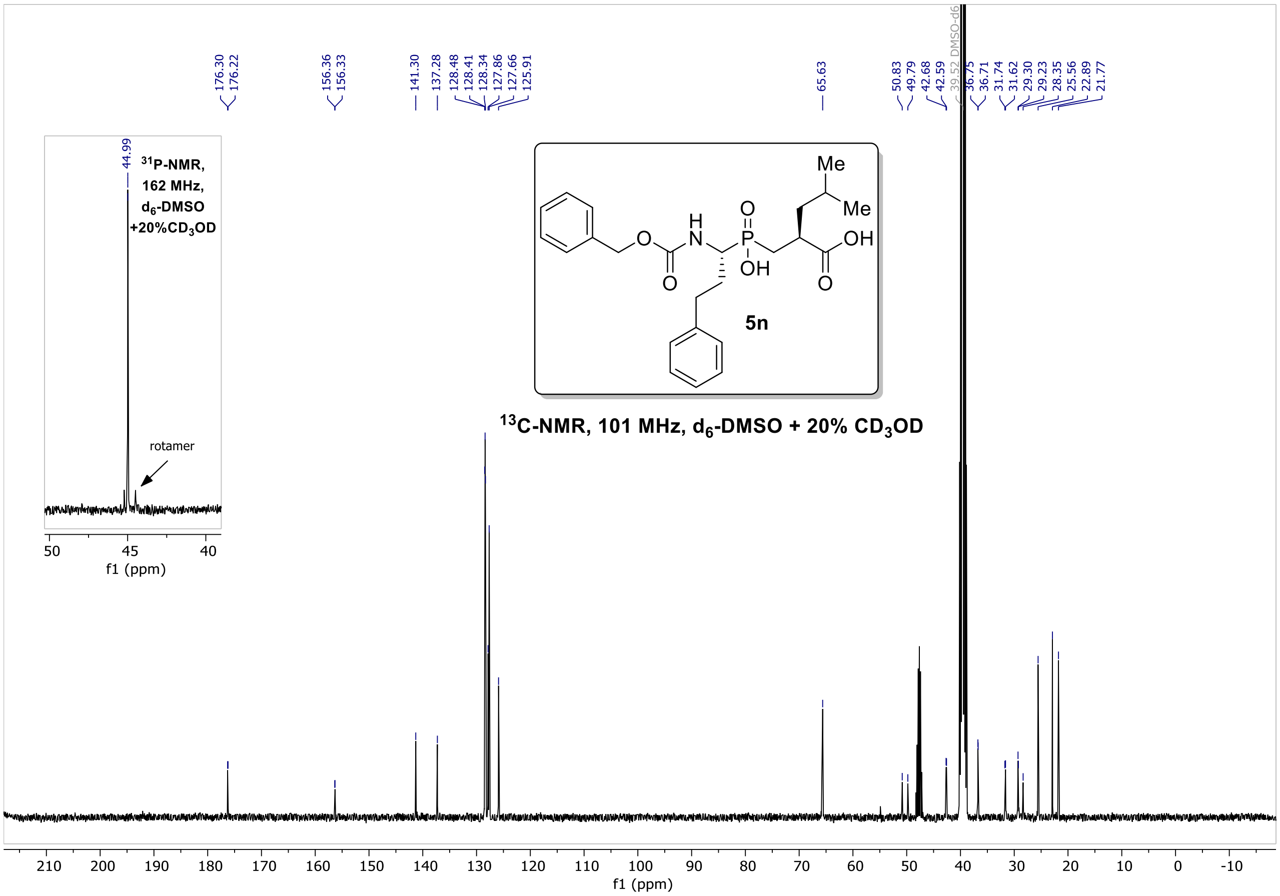

^{31}P-NMR, 162 MHz, d_6-DMSO +20%CD_3OD
rotamer
5n
^{13}C-NMR, 101 MHz, d_6-DMSO + 20% CD_3OD
f1 (ppm)

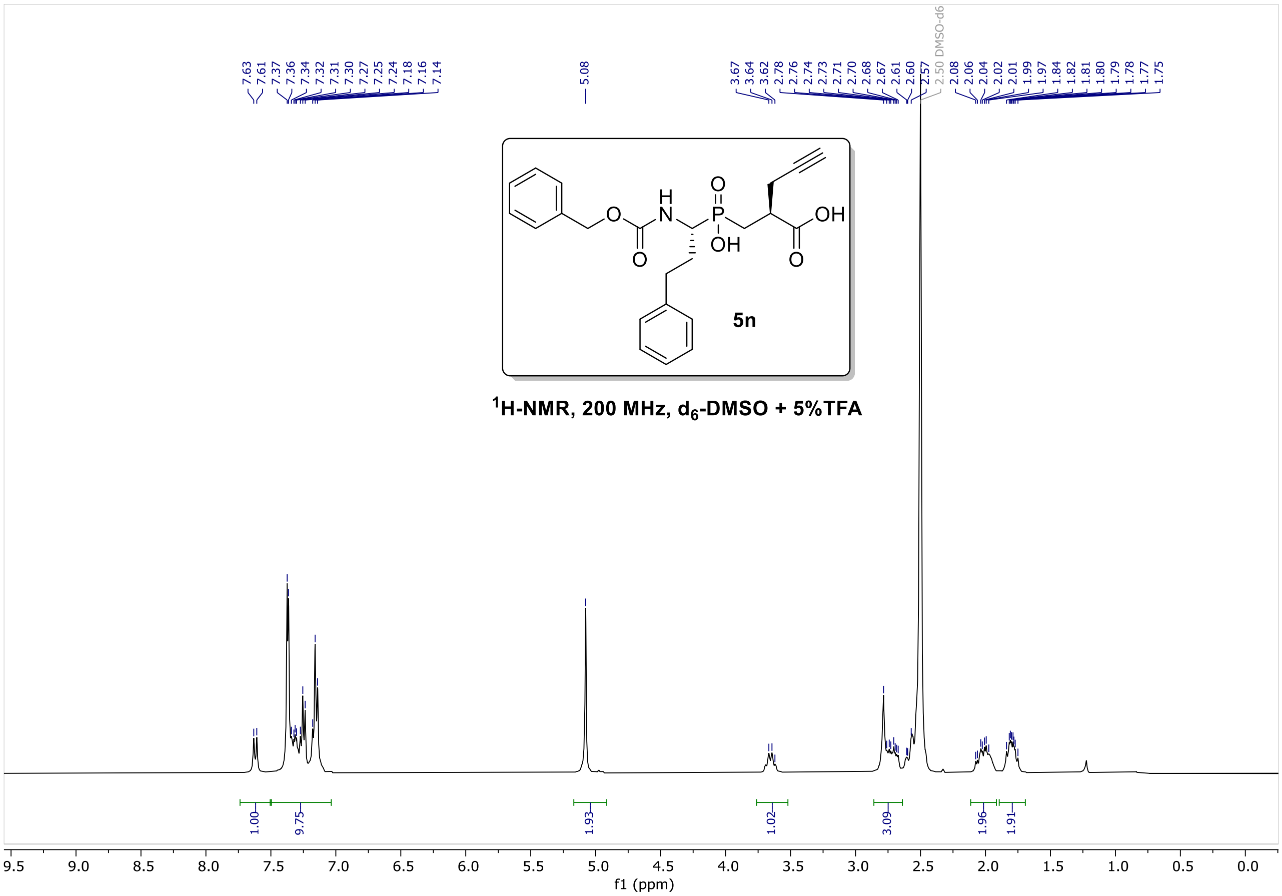
5n
^{1}H-NMR, 200 MHz, d_6-DMSO + 5%TFA
f1 (ppm)

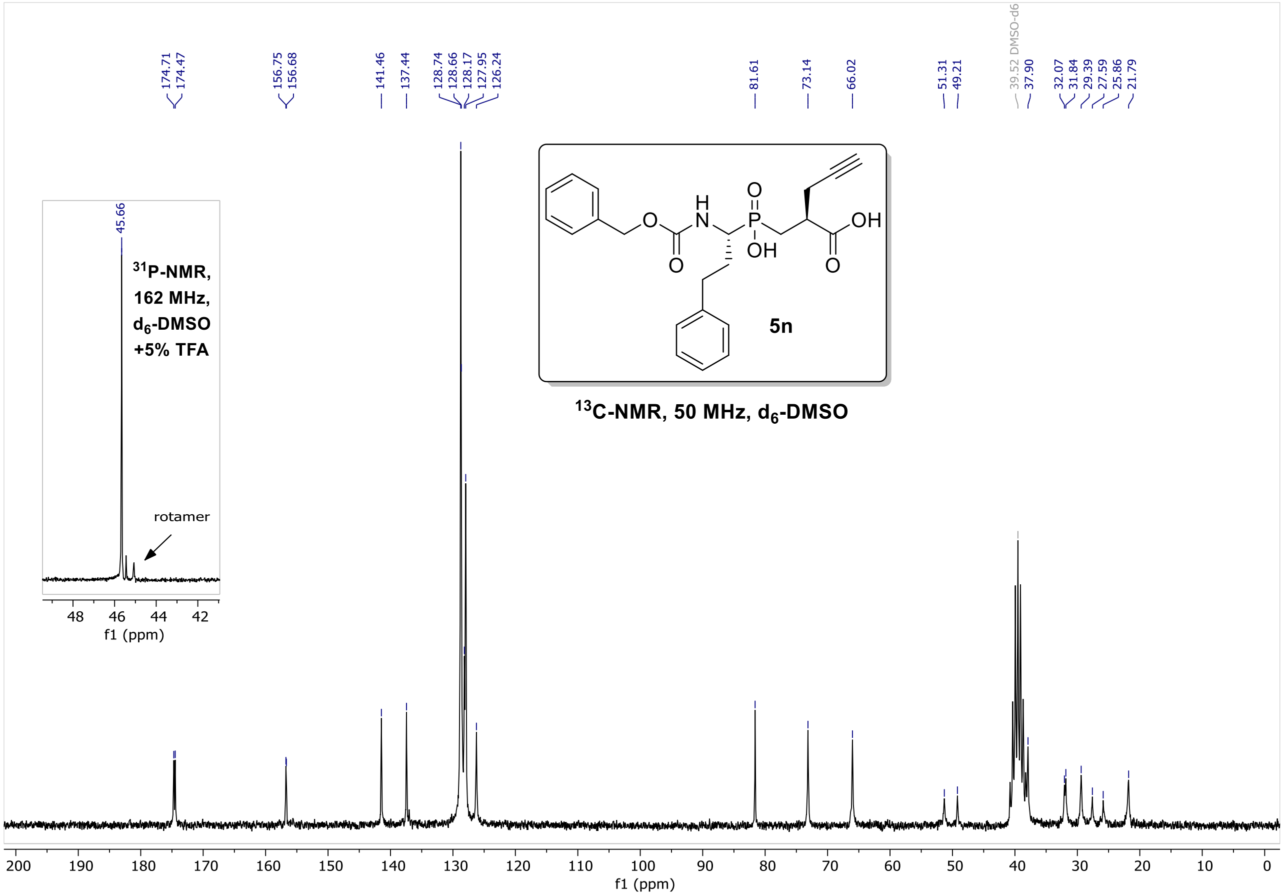
^{31}P-NMR,
162 MHz,
d_6-DMSO
+5% TFA
rotamer
5n
^{13}C-NMR, 50 MHz, d_6-DMSO
f1 (ppm)

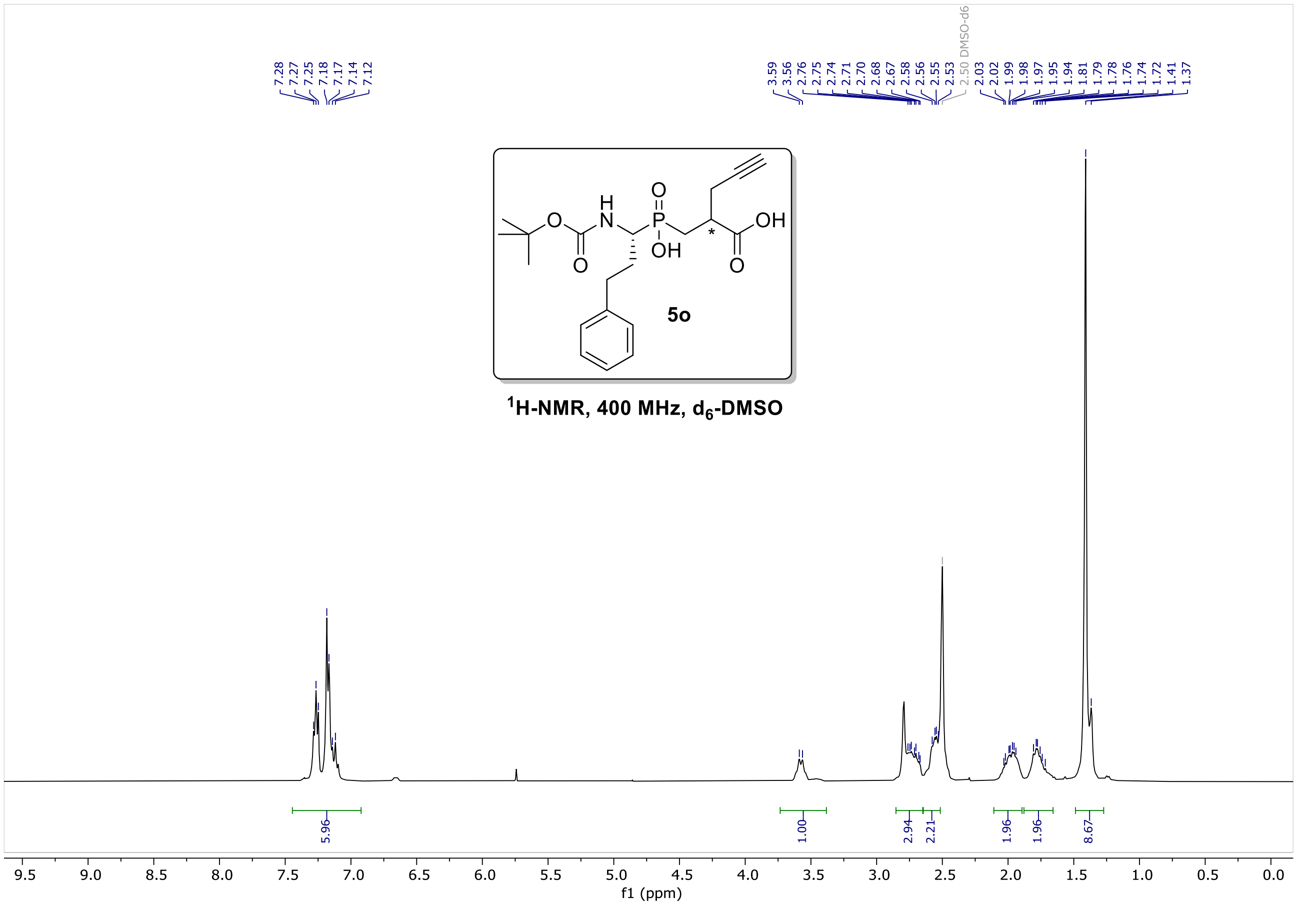

7.28
7.27
7.25
7.18
7.17
7.14
7.12
3.59
3.56
2.76
2.75
2.74
2.71
2.70
2.68
2.67
2.58
2.56
2.55
2.53
2.50 DMSO-d6
2.03
2.02
1.99
1.98
1.97
1.95
1.94
1.81
1.79
1.78
1.76
1.74
1.72
1.41
1.37
5o
¹H-NMR, 400 MHz, d6-DMSO
5.96
1.00
2.94
2.21
1.96
1.96
8.67
f1 (ppm)

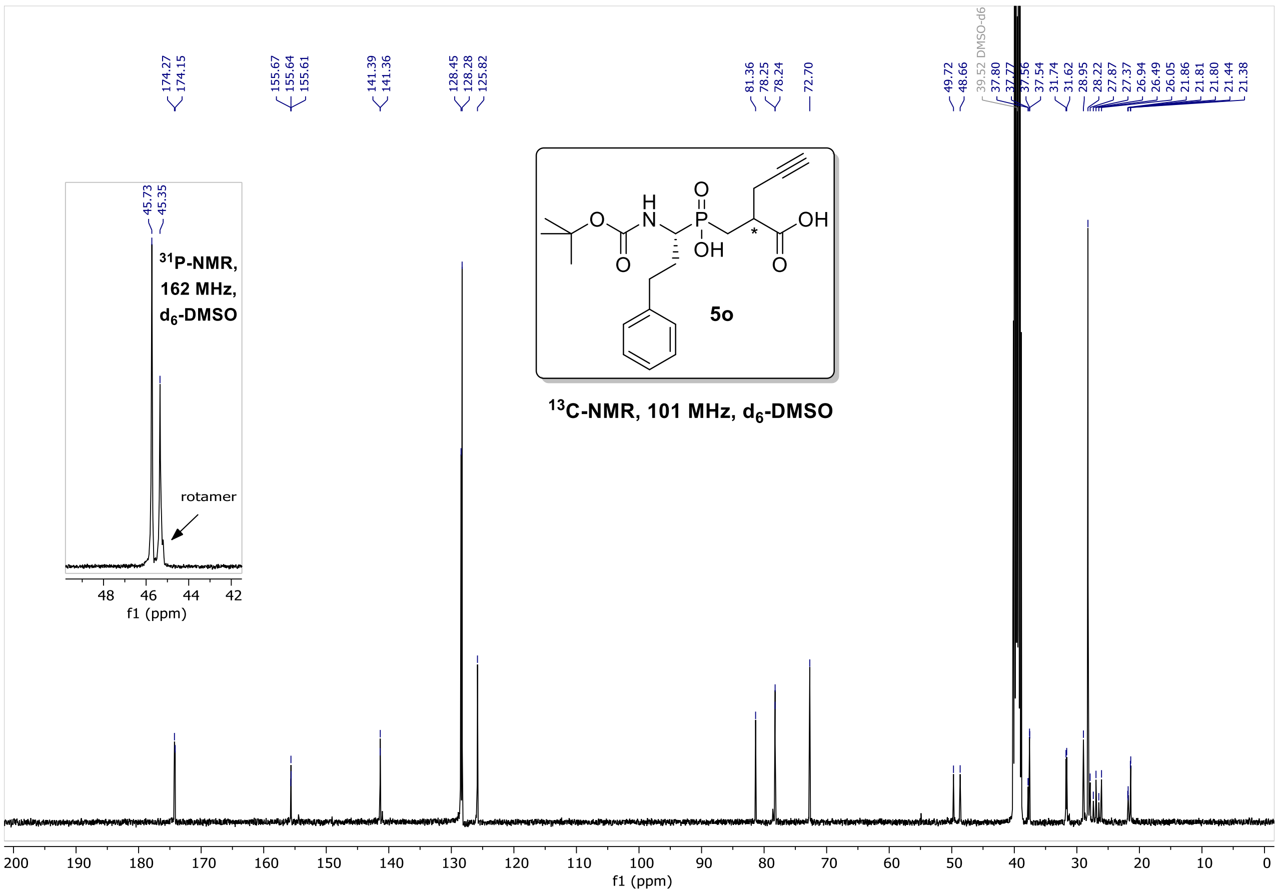

174.27
174.15
155.67
155.64
155.61
141.39
141.36
128.45
128.28
125.82
81.36
78.25
78.24
72.70
49.72
48.66
39.52 DMSO-d6
37.80
37.77
37.56
37.54
31.74
31.62
28.95
28.22
27.87
27.37
26.94
26.49
26.05
21.86
21.81
21.80
21.44
21.38
45.73
45.35
³¹P-NMR, 162 MHz, d6-DMSO
rotamer
f1 (ppm)
5o
¹³C-NMR, 101 MHz, d6-DMSO
f1 (ppm)

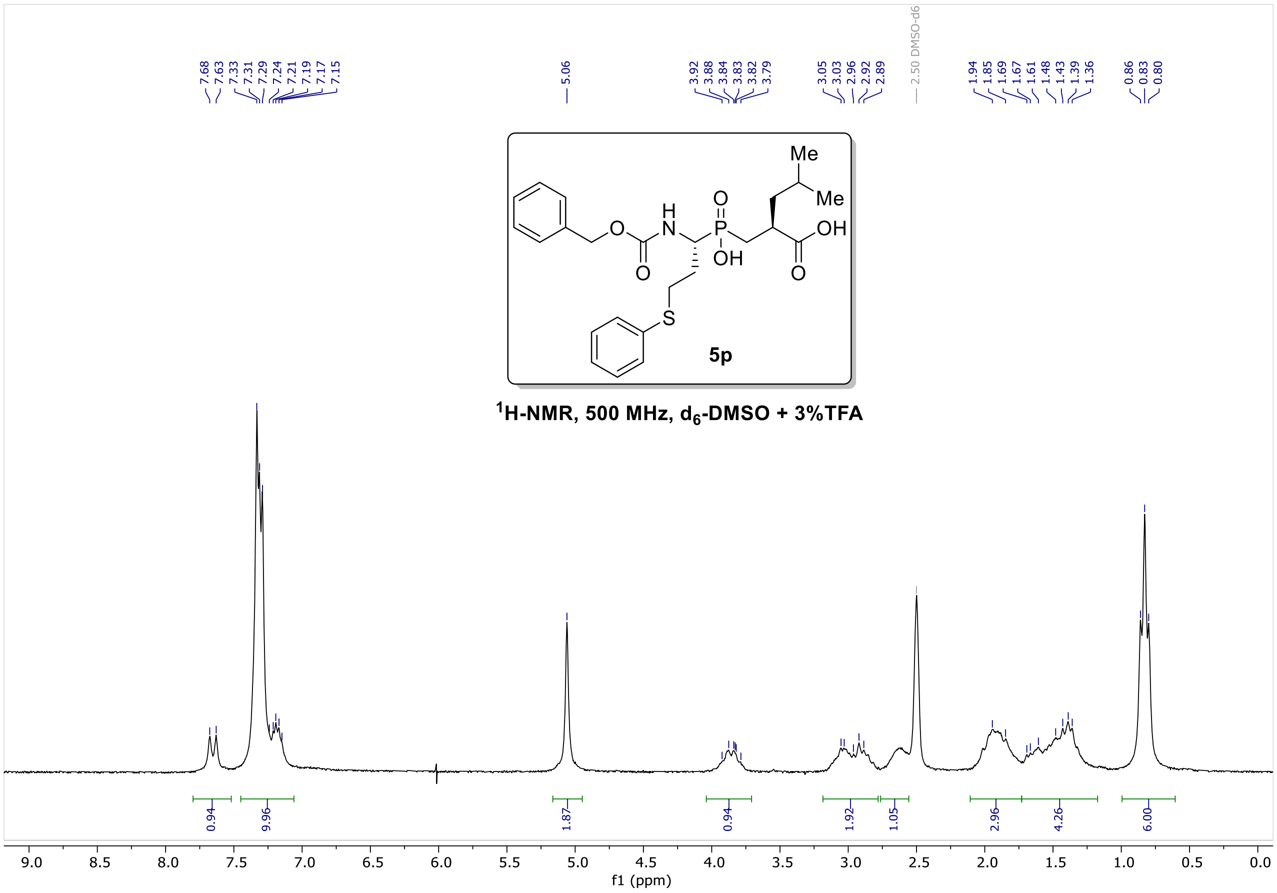
7.68 7.63 7.33 7.31 7.29 7.24 7.21 7.19 7.17 7.15
5.06
3.92 3.88 3.84 3.83 3.82 3.79
3.05 3.03 2.96 2.92 2.89
2.50 DMSO-d6
1.94 1.85 1.69 1.67 1.61 1.48 1.43 1.39 1.36
0.86 0.83 0.80
Me
Me
H
N
O
P
O
OH
OH
O
O
S
5p
^{1}H-NMR, 500 MHz, d_6-DMSO + 3%TFA
0.94 9.96 1.87 0.94 1.92 1.05 2.96 4.26 6.00
f1 (ppm)

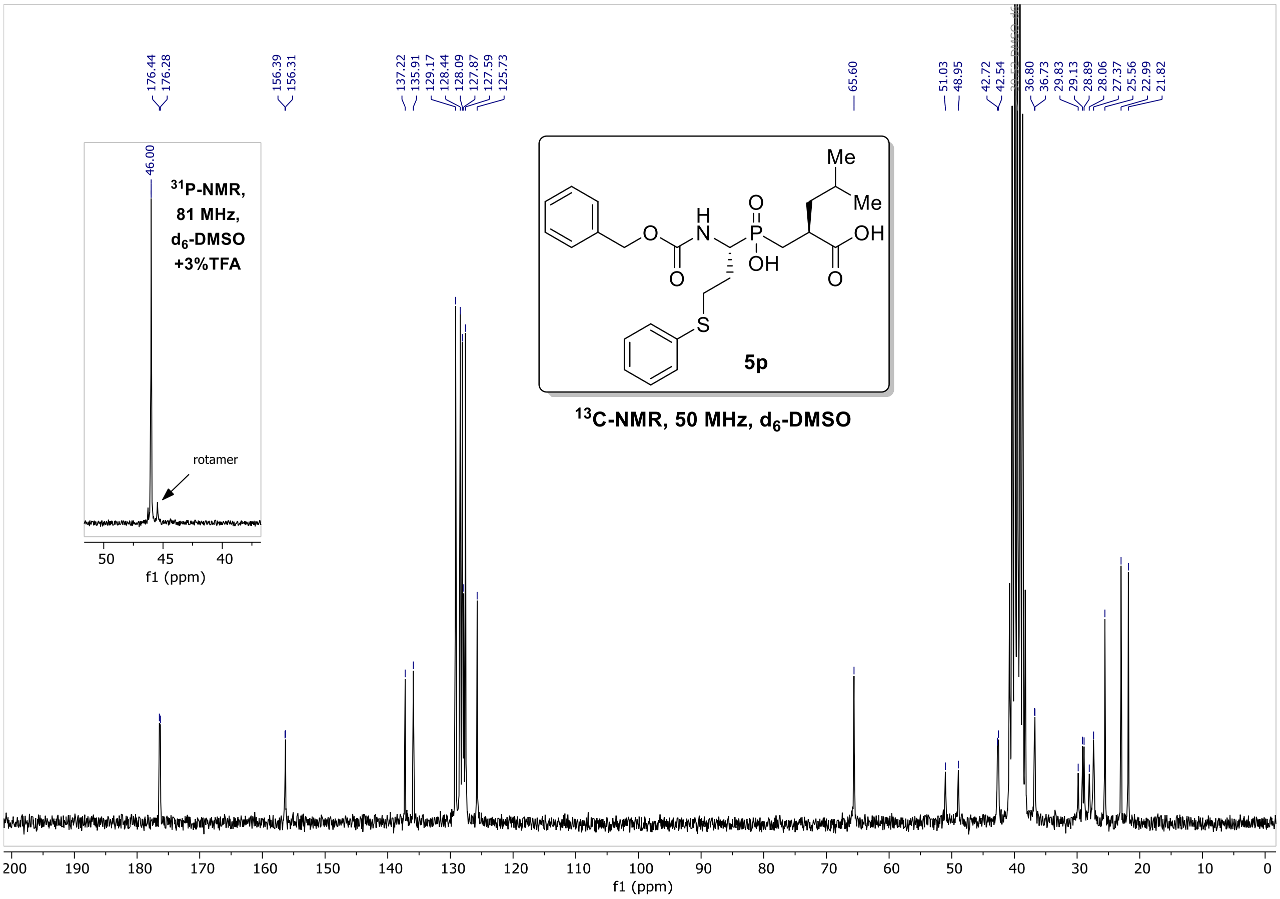
176.44 176.28
156.39 156.31
137.22 135.91 129.17 128.44 128.09 127.87 127.59 125.73
65.60
51.03 48.95
42.72 42.54
36.80 36.73 29.83 29.13 28.89 28.06 27.37 25.56 22.99 21.82
46.00
^{31}P-NMR, 81 MHz, d_6-DMSO +3%TFA
rotamer
f1 (ppm)
Me
Me
H
N
O
P
O
OH
OH
O
O
S
5p
^{13}C-NMR, 50 MHz, d_6-DMSO
f1 (ppm)

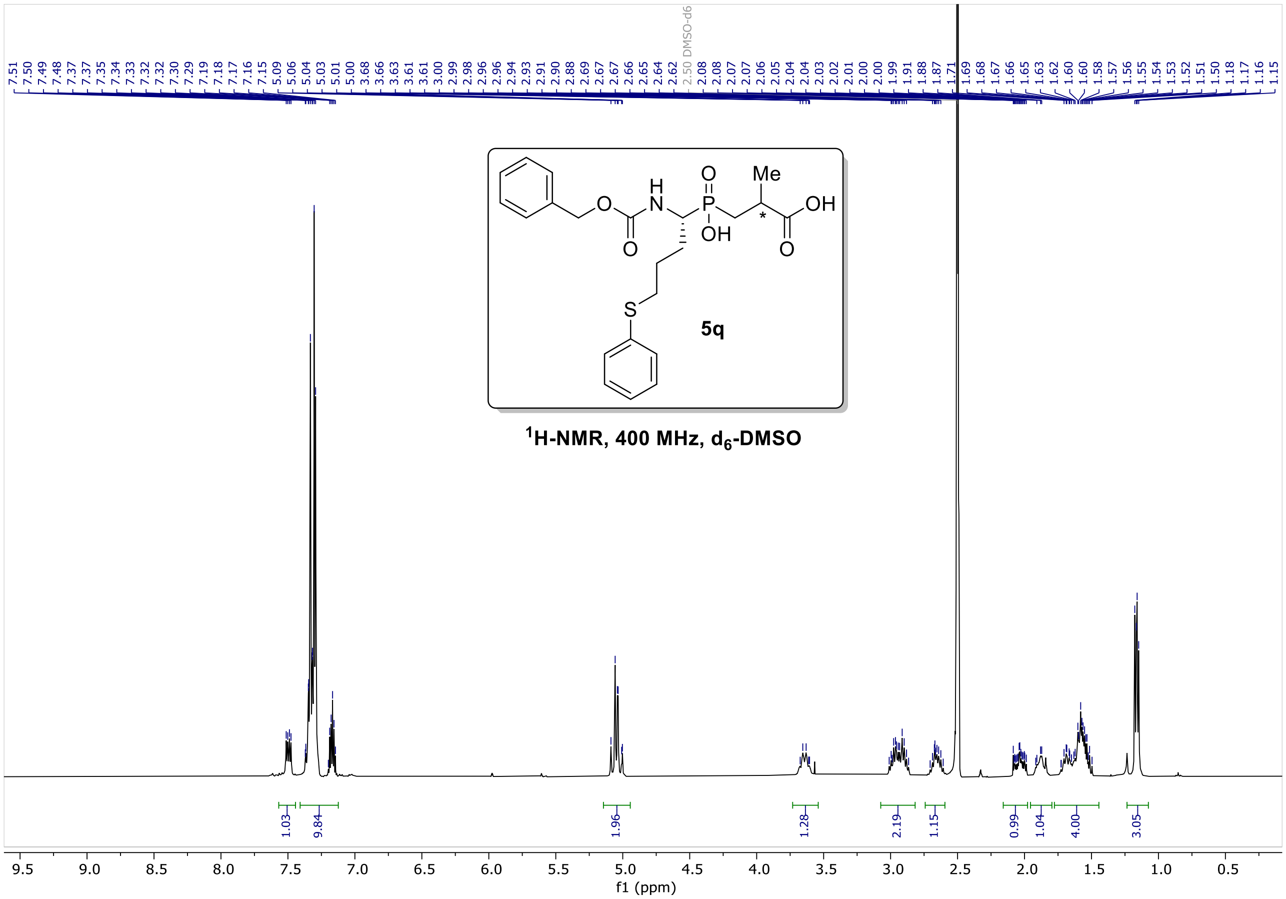
5q
1H-NMR, 400 MHz, d6-DMSO
f1 (ppm)

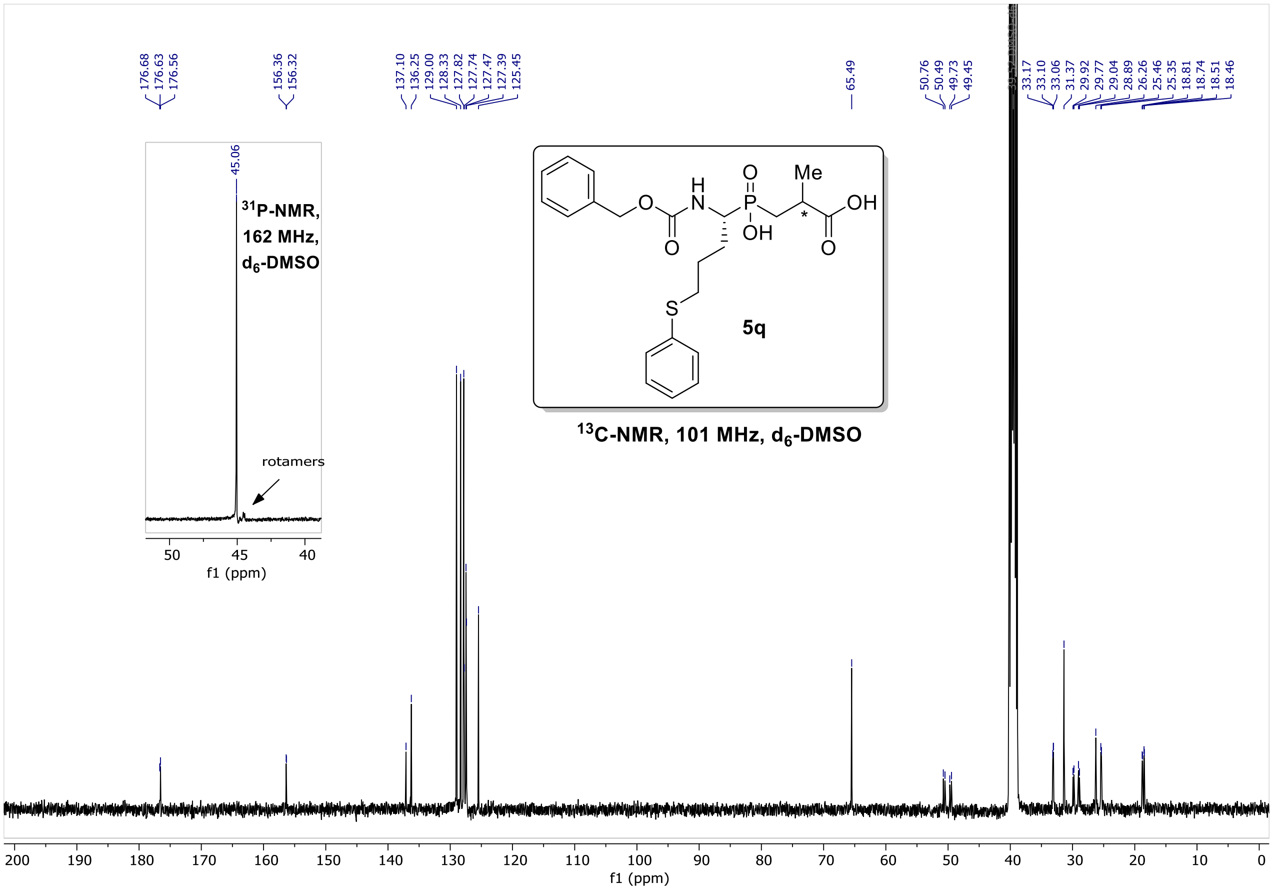
31P-NMR, 162 MHz, d6-DMSO
rotamers
5q
13C-NMR, 101 MHz, d6-DMSO
f1 (ppm)

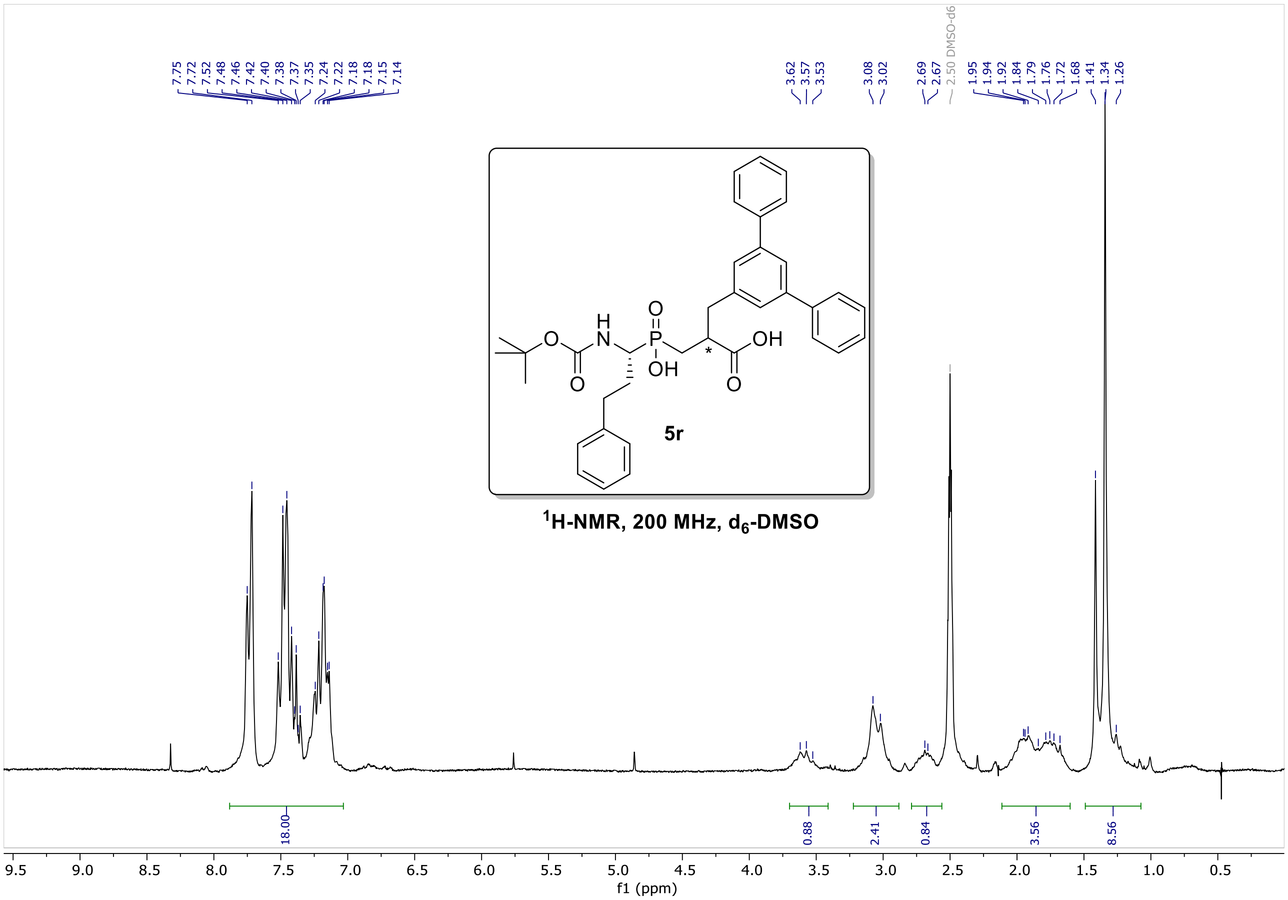
7.75 7.72 7.52 7.48 7.46 7.42 7.40 7.38 7.37 7.35 7.24 7.22 7.18 7.18 7.15 7.14
3.62 3.57 3.53
3.08 3.02
2.69 2.67
2.50 DMSO-d6
1.95 1.94 1.92 1.84 1.79 1.76 1.72 1.68 1.41 1.34 1.26
H
O
P
OH
O
OH
O
O
N
*
5r
^{1}H-NMR, 200 MHz, d_6-DMSO
18.00
0.88
2.41
0.84
3.56
8.56
9.5 9.0 8.5 8.0 7.5 7.0 6.5 6.0 5.5 5.0 4.5 4.0 3.5 3.0 2.5 2.0 1.5 1.0 0.5
f1 (ppm)

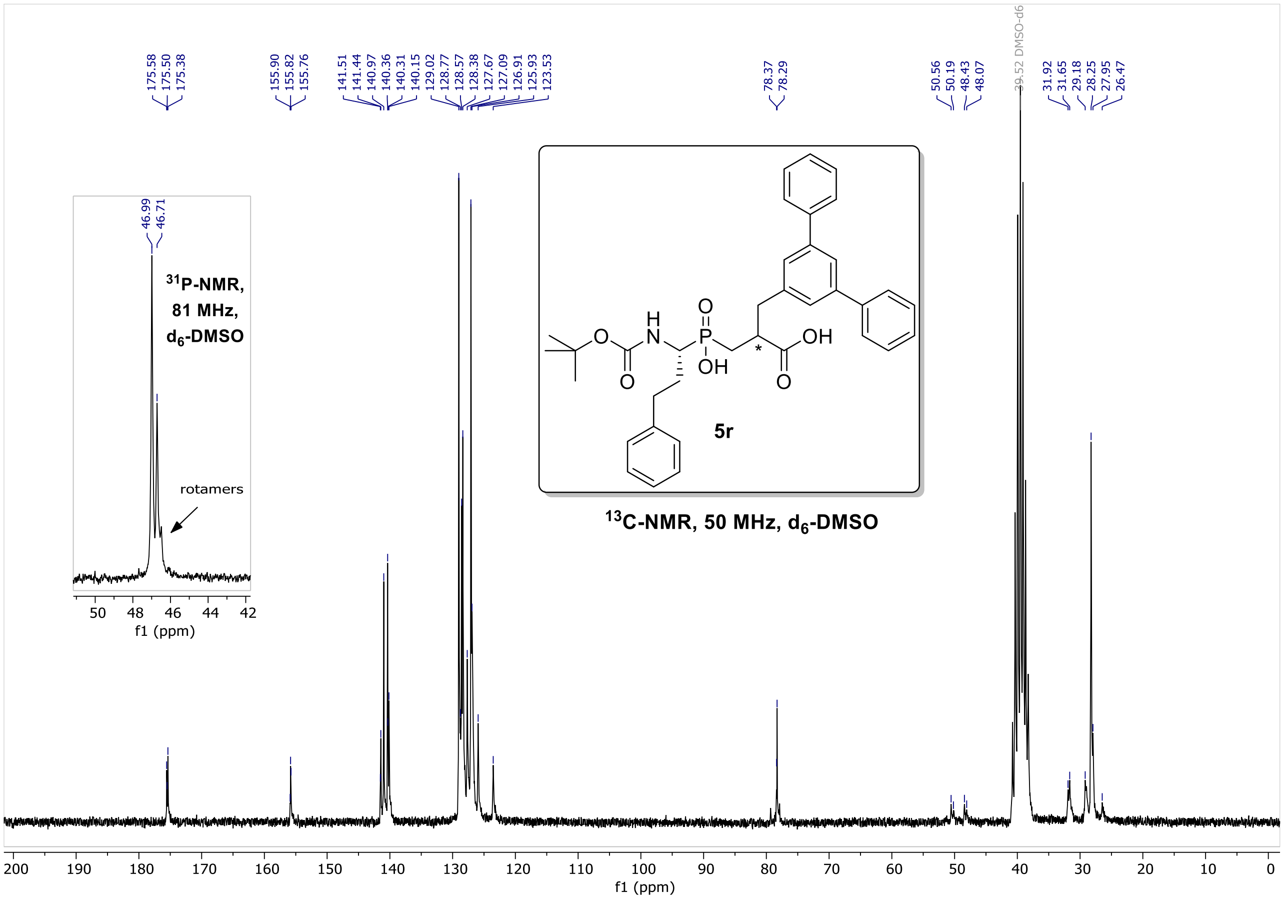
175.58 175.50 175.38
155.90 155.82 155.76
141.51 141.44 140.97 140.36 140.31 140.15 129.02 128.77 128.57 128.38 127.67 127.09 126.91 125.93 123.53
78.37 78.29
50.56 50.19 48.43 48.07
39.52 DMSO-d6
31.92 31.65 29.18 28.25 27.95 26.47
46.99 46.71
^{31}P-NMR, 81 MHz, d_6-DMSO
rotamers
50 48 46 44 42
f1 (ppm)
H
O
P
OH
O
OH
O
O
N
*
5r
^{13}C-NMR, 50 MHz, d_6-DMSO
200 190 180 170 160 150 140 130 120 110 100 90 80 70 60 50 40 30 20 10 0
f1 (ppm)

References

1. Goh, K. K. K.; Kim, S.; Zard, S. Z., Free-Radical Variant for the Synthesis of Functionalized 1,5-Diketones. *Org. Lett.* **2013,** 15, (18), 4818-4821.
